# Discovery of a Novel Chromone Enantiomer and the Precursors of Nonactic Acid from the Coral-Reef-Derived *Streptomyces* sp. SCSIO 66814

**DOI:** 10.3390/md22040181

**Published:** 2024-04-17

**Authors:** Wenping Ding, Yanqun Li, Xingyu Li, Jiajia Yin, Songbiao Shi, Xinpeng Tian, Si Zhang, Hao Yin

**Affiliations:** 1 CAS Key Laboratory of Tropical Marine Bio-Resources and Ecology, South China Sea Institute of Oceanology, Chinese Academy of Sciences, Guangzhou 510301, China; dingwenping19@mails.ucas.ac.cn (W.D.); liyanqun20@mails.ucas.ac.cn (Y.L.); bsy_lixingyu@126.com (X.L.); yinjiajia22@mails.ucas.ac.cn (J.Y.); shisong0615@163.com (S.S.); xinpengtian@scsio.ac.cn (X.T.); 2 Southern Marine Science and Engineering Guangdong Laboratory (Guangzhou), Guangzhou 511458, China

**Keywords:** *Streptomyces* sp. SCSIO 66814, metabolites, enantiomer, structural elucidation, biosynthesis of nonactic acid

## Abstract

Three pairs of enantiomers (**1**–**3**)—the new 12*R*-aloesol (**1a**) and two new fatty acids (**2** and **3**)—and one new natural product (**4**) together three known compounds (**5**–**7**) were isolated from a coral-reef-derived *Streptomyces* sp. SCSIO 66814. Their structures were determined through extensive spectroscopic analysis, chiral analysis, and single-crystal X-ray diffraction data. Compounds **2** and **3** were presumed to be intermediates for further generating homononactic acid (**5**) and nonactic acid, and the latter two molecules were able to act as precursors to form macrotetrolides with remarkable biological activity. The isolation of related precursors, compounds **2**–**5**, provided more evidence to support the proposal of a plausible biosynthetic pathway for nonactic acid and its homologs. Additionally, (+)-**1** exhibited a weak activity against DPPH radicals.

## 1. Introduction

**Figure 1:**
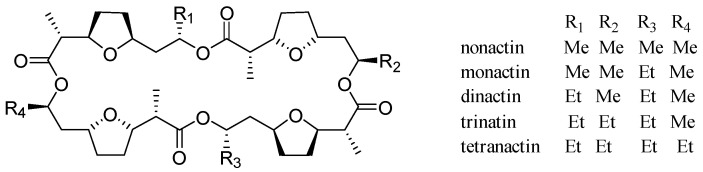
Chemical structures of nonactin and its homologs.

Nonactin and its homologs (Figure 1) are a class of ionophore antibiotics known as the macrotetrolides [1], which are composed of four monomers, the latter being either nonactic acid or homononactic acid. These antibiotics are mainly synthesized by *Streptomyces* strains, including *Streptomyces griseus* subsp. *griseus* ETH A7796. Nonactin exhibits significant antibacterial activity and has been shown to be effective in combating tumors and inhibiting the P-glycoprotein-mediated drug resistance in cancer cells [2]. The biosynthetic pathway for nonactin was originally established by stable isotope feeding experiments with primary metabolites and more advanced precursors [3]. However, some steps of the pathway remain unconfirmed, mainly due to a lack of direct isolation of intermediates in the pathway. The two important precursors, nonactic acid and homononactic acid, are assembled from acetate, propionate, and succinate through a pathway based on polyketide biosynthesis [4]. Nevertheless, the precursors for nonactic acid and homononactic acid are not directly isolated from microorganisms to the best of our knowledge, instead relying mainly on chemical synthesis of substances such as (6*R*, 8*R*)- and (6*S*, 8*S*)-2-methyl-6, 8-dihydroxynon-2*E*-enoic acids [5] and 4, 6-diketoheptanoate derivatives [3]. The discovery of intermediates plays an important role in establishing the complete biosynthetic process for nonactin and its analogs.

**Figure 2:**
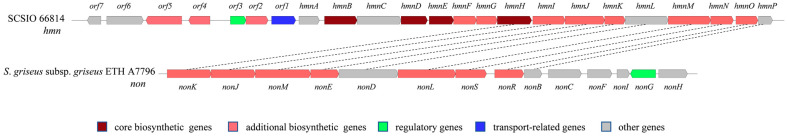
Comparative analysis of *hmn* biosynthetic gene cluster (BGC) in *Streptomyces* sp. SCSIO 66814 with reported nonactin BGC from *S*. *griseus* subsp. *griseus* ETH A7796. Homologous genes are connected by black dashed lines.

**Figure 3:**
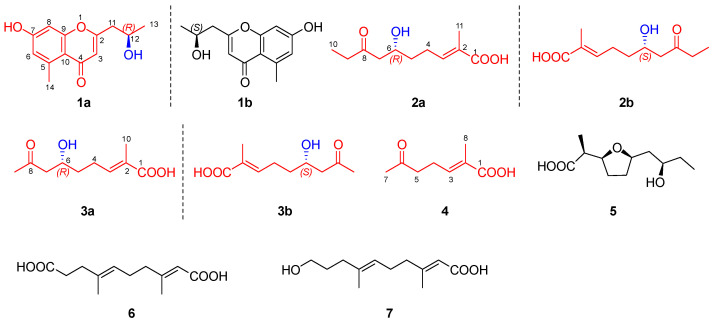
Chemical structures of compounds **1**–**7**.

As part of a continuous exploration in search of new metabolites of biological significance from marine microorganisms, we found a coral-reef-derived actinomycete *Streptomyces* sp. SCSIO66814. This strain possessed 57 biosynthetic gene clusters (BCGs), as determined using antiSMASH with a “loose” detection strictness setting, including a potential nonactin BGC (namely *hmn*) (Figure 2, Supplementary Materials: Tables S1 and S2). Interestingly, the LC-MS/MS analysis, coupled with molecular networking (MN), revealed the potential production of nonactin, monactin, and trinatin by the strain (Supplementary Materials, Table S4, Figure S5), aligning with the *hmn* BGC. The result suggested that the MRA medium could activate the *hmn* BGC. Chemical investigation of the strain led to the isolation of three pairs of enantiomers (**1**–**3**)—(±)-aloesol (**1**) and two fatty acids (**2** and **3**)—and one new natural product (**4**), along with four known compounds (**5**–**7**) (Figure 3). Their structures were established by a combination of extensive spectroscopic analysis, chiral analysis, and single-crystal X-ray diffraction data. Herein, we report the isolation, structural elucidation, and biologic activity of these compounds in addition to the plausible biosynthetic pathway of compounds **2**–**5**.

## 2. Results and Discussion

### 2.1. Analysis of Molecular Networking

**Figure 4:**
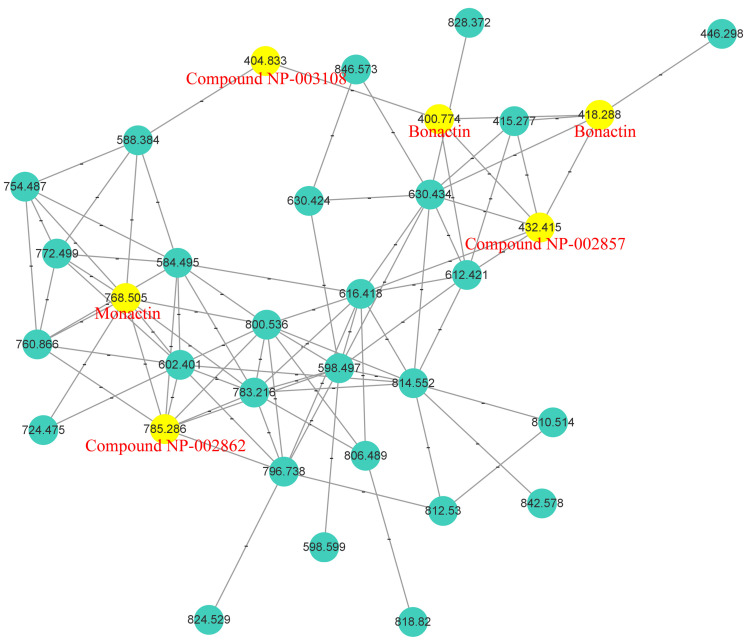
Partially enlarged cluster-node graph of molecular networking. The numbers on nodes represent parent ions, and the nodes highlighted in yellow were annotated by GNPS.

The MN displayed a large cluster presumably associated with nonactin and its homologs, comprising 33 nodes (Figure 4). Within this cluster, six nodes corresponding to parent ions at *m*/*z* 785.3, 768.5, 432.4, 418.3 (and 400.8), and 404.8 were annotated by GNPS as compound NP-002862, monactin, compound NP-002857, bonactin, and compound NP-003108, respectively. These compounds are nonactin derivatives, corresponding to the *hmn* BGC. Furthermore, three key ions were detected in our total ion chromatogram (TIC) at 37.8, 39.4, and 42.2 min, respectively (Supplementary Materials, Figure S5). Detailed MS/MS analysis showed that the parent ion at *m*/*z* 754.4755 was nonactin, based on its four key fragments at *m*/*z* 553.3369, 369.2274, 185.1177, and 167.1058 (Supplementary Materials, Figure S6). Similarly, the parent ions at *m*/*z* 768.4909 and 796.5213 were identified as monactin and trinatin, respectively, based on MS/MS fragments at *m*/*z* 567.3557, 383.2415, 369.2279, 199.1350, 185.1188, and 167.1067, and at *m*/*z* 581.3705, 397.2600, 383.2450, 199.1329, 185.1180, and 181.1231, respectively (Supplementary Materials, Figure S5).

### 2.2. Structural Elucidation

**Figure 5:**
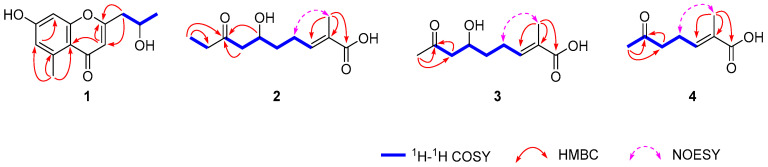
Key ^1^H–^1^H COSY, HMBC, and NOESY correlations of **1**–**4**.

**Figure 6:**
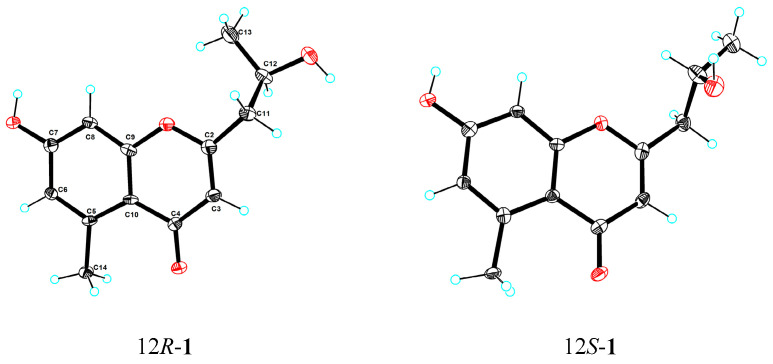
ORTEP diagram for the crystal structures of 12*R*-**1** (**1a**) and 12*S*-**1** (**1b**).

Compound **1** was isolated as a colorless crystal (MeOH-H_2_O). It was assigned the molecular formula C_13_H_14_O_4_ based on its HRESIMS (*m*/*z* 235.0968 [M + H]^+^, calcd for C_13_H_15_O_4_ 235.0965), suggesting seven degrees of unsaturation. The ^1^H NMR spectrum (Table 1) showed three olefinic proton signals at *δ*_H_ 6.65 (H-8, d, 2.4), *δ*_H_ 6.63 (H-6, d, 2.4), and *δ*_H_ 6.06 (H-3, s), one oxygenated methine proton signal at *δ*_H_ 4.18 (H-12, dqd, 7.9, 6.2, 4.9), and two methyl proton signals at *δ*_H_ 2.72 (H-14, s) and *δ*_H_ 1.27 (H-13, d, 6.2). The ^13^C and DEPT spectra displayed thirteen carbon signals, comprising two methyl carbons, one methylene carbon, four methine carbons, and six quaternary carbons. The NMR data were similar with those of aloesol [2-(2′-hydroxypropyl)-5-methyl-7-hydroxychromone] [6], and the same planar structure was confirmed by using 2D NMR spectra (Figure 5). The opposite sign for the specific rotation of ${\left[\alpha \right]}_{D}^{25}$ − 11.4 (c 0.1, MeOH), in comparison to that of ${\left[\alpha \right]}_{D}^{21}$ + 38.4 (c 0.89, MeOH) reported in the literature, suggested that compound **1** may be the enantiomer of aloesol (12*S*), with the configuration of C-12 to be *R*. Interestingly, the space group of crystals of compound **1** was determined to be triclinic/P1, and one unit cell displayed four molecules containing three 12*R*-aloesol and one 12*S*-aloesol, indicating that compound **1** was a mixture (Figure 6). The chiral HPLC analysis (Supplementary Materials, Figure S1) revealed the ratio of 12*R*-aloesol and 12*S*-aloesol to be 3:1, in accordance with the results of single-crystal X-ray diffraction. The pair of enantiomers was further separated by a chiral column, and the novel 12*R*-aloesol (**1a**) exhibited specific rotation of ${\left[\alpha \right]}_{D}^{25}$ − 15.5 (c 0.06, MeOH).

Compound **2** was obtained as a yellow oil and possessed the molecular formula C_11_H_18_O_4_ with three degrees of unsaturation, as deduced by its HRESIMS data (*m*/*z* 213.1136 [M − H]^−^, calcd for C_11_H_17_O_4_ 213.1132). The ^1^H NMR spectrum (Table 1) displayed one olefinic proton signal at *δ*_H_ 6.75 (H-3, tq, 7.6, 1.6), one oxygenated methine proton signal at *δ*_H_ 4.04 (H-6, tt, 8.4, 4.4), and two methyl proton signals at *δ*_H_ 1.82 (H-11, br s) and *δ*_H_ 1.01 (H-10, t, 7.3). The ^13^C and DEPT spectra exhibited eleven carbon signals, including two methyl carbons, four methylene carbon, two methine carbons, and three quaternary carbons. The two structural fragments of C3-C4-C5-C6-C7 and C9-C10 were uncovered by ^1^H–^1^H COSY correlations (Figure 5). The planar structure of **2** was constructed by the key HMBC correlations from H-11 to C-1 (*δ*_C_ 172.4), C-2 (*δ*_C_ 130.0), and C-3 (*δ*_C_ 142.5), and from H-6, H-7, and H-10 to C-8 (*δ*_C_ 212.9) (Figure 5). The *E*-configuration of the double bond between C-2 and C-3 was determined by the key NOE correlation of H-4 with H-11 (Figure 5). The structure of **2** was similar to (5*R*)-(2*E*)-5-hydroxy-6-keto-2-methyl-2-heptenoic acid [7], with the only difference being the presence of three additional methylenes in **2**. The specific rotation for **2** was measured to be ${\left[\alpha \right]}_{D}^{25}$ + 0.4 (c 0.1, MeOH), suggesting that **2** was a racemate. The profiles of chiral HPLC analysis (Supplementary Materials, Figure S2) further supported the above result. The mixture was further separated by a chiral column, resulting in 6*R*-**2** (**2a**) with a specific rotation of ${\left[\alpha \right]}_{D}^{25}$ − 9.3 (c 0.04, MeOH)and 6*S*-**2** (**2b**) with a specific rotation of ${\left[\alpha \right]}_{D}^{25}$ + 7.8 (c 0.03, MeOH). In comparison, (5*R*)-(2*E*)-5-hydroxy-6-keto-2-methyl-2-heptenoic acid exhibited a specific rotation of ${\left[\alpha \right]}_{D}^{20}$ − 26.4 (c 0.03, MeOH) [7]. Additionally, to obtain more evidence, the specific rotations of **2a** and **2b** were calculated. The result showed a value of −15.7 for **2a** and +27.9 for **2b**, further supporting the aforementioned conclusion.

Compound **3** was isolated as a yellow oil and possessed the molecular formula C_10_H_16_O_4_ with three degrees of unsaturation based on its HRESIMS data (*m*/*z* 223.0945 [M + Na]^+^, calcd for C_10_H_16_O_4_Na 223.0941). The ^1^H NMR spectrum (Table 2) displayed one olefinic proton signal at *δ*_H_ 6.72 (H-3, tq, 7.5, 1.5), one oxygenated methine proton signal at *δ*_H_ 4.04 (H-6, m), and two methyl proton signals at *δ*_H_ 2.17 (H-9, s) and *δ*_H_ 1.82 (H-11, br s). The ^13^C and DEPT spectra exhibited ten carbon signals, including two methyl carbons, three methylene carbon, two methine carbons, and three quaternary carbons. The detailed NMR analysis revealed that the NMR data of **3** were similar to those of **2**, with the only difference being the absence of one methylene in **3** compared to **2**. The result was further supported by 2D NMR spectra (Figure 5). The key NOE correlation of H-4 with H-11 revealed the configuration of the double bond between C-2 and C-3 to be *E*. Similar to compound **2**, the specific rotation of ${\left[\alpha \right]}_{D}^{25}$ + 3.7 (c 0.1, MeOH) in combination with the result for the chiral HPLC profile (Supplementary Materials, Figure S3) showed that compound **3** was also a racemate. Additionally, compound **3** was identified as a new natural product. Small quantities of it had been previously detected through the conversion of (6*R*, 8*R*)- and (6*S*, 8*S*)-2-methyl-6, 8-dihydroxynon-2*E*-enoic acids in feeding experiments with shake cultures of *S*. *griseus* ETHA7796 [5]. However, the original paper only provided the planar structure without detailed spectroscopic data. Furthermore, the mixture was separated by a chiral column, resulting in 6*R*-**3** (**3a**) with a specific rotation of ${\left[\alpha \right]}_{D}^{25}$ − 7.2 (c 0.04, MeOH), and 6*S*-**3** (**3b**) with a specific rotation of ${\left[\alpha \right]}_{D}^{25}$ + 9.5 (c 0.06, MeOH).

Compound **4** was obtained as a yellow oil and assigned the molecular formula C_8_H_12_O_3_ with three degrees of unsaturation, as deduced by its HRESIMS data (*m*/*z* 155.0707 [M − H]^−^, calcd for C_8_H_11_O_3_ 155.0714). The ^13^C and DEPT spectra (Table 2) exhibited eight carbon signals, including two methyl carbons, two methylene carbon, one methine carbon, and three quaternary carbons. Its NMR data were similar to those of compounds **2** and **3**, showing **4** to be an unsaturated fatty acid. Its planar structure was established by 2D NMR spectra (Figure 5), and the *E*-configuration of the double bond was confirmed. The structure of **4** closely resembled (5*R*)-(2*E*)-5-hydroxy-6-keto-2-methyl-2-heptenoic acid [7], differing only with the absence of one hydroxyl in **4.** Compound **4**, namely (*E*)-2-methyl-6-oxohept-2-enoic acid [8], was a new natural product, previously synthesized as an intermediate to generate (*Z*)-2-methyl-6-oxohept-2-enoic acid.

Compounds **5**–**7** were assigned as homononactic acid (**5**) [9], *trans*, *trans*-3, 7-dimethyl-2, 6-decadien-1, 10-dioic acid (**6**) [10], and *trans*, *trans*-10-hydroxy-3, 7-dimethyl-2, 6-decadienoic acid (**7**) [11], respectively, through a comparison of their NMR data with those reported in the literature. Interestingly, compounds **6** and **7** were reported to be components of the pheromonal secretion of the male monarch butterfly. It was originally believed that the monarch synthesized these molecules. However, besides our work, compound **6** and its derivative have been discovered from a marine-derived *Streptomyces* sp. [12], suggesting that the two molecules are likely synthesized by symbiotic microorganisms of the monarch butterfly. Additionally, compound **5** was considered as a racemate owing to its lower specific rotation of ${\left[\alpha \right]}_{D}^{25}$ + 3.9 (c 0.1, MeOH) and its racemic precursor (compound **2**) together with a comparison with references [9].

### 2.3. Putative Biosynthetic Pathway for Homononactic Acid and Nonactic Acid

**Figure 7:**
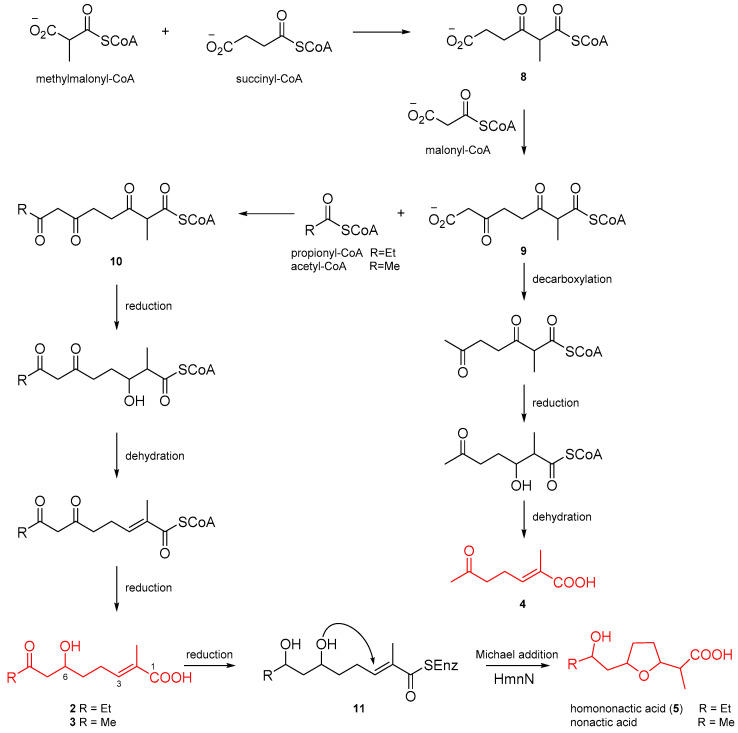
Proposed biosynthetic pathway for homononactic acid and nonactic acid.

The enantiomeric nonactic acid and its homologs have been confirmed to be of polyketide origin by isotope labeling experiments, and biochemical investigations have shown that NonS in a cell-free preparation can convert 2-methyl-6, 8-dihydroxynon-2*E*-enoic acid into nonactic acid [5,13]. However, the lack of isolation of intermediates makes it difficult to establish the biosynthetic process for nonactic acid and its homologs. Based on the isolated intermediates (**2**–**5**) from *Streptomyces* sp. SCSIO 66814, we proposed a putative biosynthetic pathway for homononactic acid and nonactic acid (Figure 7). The three putative substrates, methylmalonyl-CoA, succinyl-CoA, and malonyl-CoA, undergoes successive condensation reactions to synthesize intermediate **9**. The intermediate **9** sequentially undergoes decarboxylation, ketone reduction, and dehydration reactions to generate compound **4**. Additionally, the formation of intermediate **10** occurs through a condensation reaction of **9** with either acetyl-CoA or propionyl-CoA. Similar to the formation of compound **4**, compounds **2** and **3** are derived from intermediate **10** through ketone reduction and dehydration reactions. Subsequently, compounds **2** and **3** are subjected to a reductive reaction, converting the ketone at C-8 to a hydroxyl group, yielding intermediate **11**. The formation of the furan ring in nonactic acid and its homolog are putatively catalyzed by HmnN, a NonS homolog enzyme (90% identity), wherein the substrate, a thioester analog of intermediate **11** instead of free acid, undergoes an intramolecular Michael addition reaction [14]. Ultimately, the enzyme complex is hydrolyzed, releasing homononactic acid (**5**) and nonactic acid.

### 2.4. Biological Activities

All isolated compounds were tested for antibacterial activity against two Gram-positive and two Gram-negative bacteria (*Bacillus subtilis*, *Staphylococcus aureus*, *Vibrio alginolyticus*, and *Escherichia coli*), and none of them presented any obvious antibacterial activity. Furthermore, the free radical scavenging activities of all compounds against DPPH radicals were also evaluated. The results (Table 3) revealed that only (+)-**1** (**1b**) displayed a weak activity against DPPH radicals, with a scavenging rate of 34.42% at a concentration of 200 µg/mL.

## 3. Materials and Methods

### 3.1. General Experimental Procedures

The same general procedures were used as those described previously [15].

### 3.2. Microorganism and Growth Conditions

SCSIO 66814 was obtained from stony coral collected from the South China Sea. The 16S rRNA sequence of SCSIO 66814 revealed that the strain belonged to *Streptomyces* sp. and exhibited 100% identity with *Streptomyces cavourensis* NBRC 13026(T) (GenBank accession number: AB184264.1). A 40 L scale fermentation was carried out in liquid medium (soluble starch 20 g/L, glucose 10 g/L, malt extract 10 g/L, maltose 10 g/L, corn steep liquor 5 g/L, CaCO_3_ 2 g/L, sea salt 30 g/L) using Erlenmeyer flasks at 28 °C for 7 days with a shaking rate of 180 rpm.

### 3.3. Bioinformatic Analysis

The complete genome of the strain SCSIO 66814 was sequenced using a combination of PacBio RS and Illumina sequencing platforms at Shanghai Biozeron Biotechnology Co., Ltd. (Shanghai, China). The *hmn* BCG predicted by the antiSMASH platform was deposited in GenBank under accession number PP098209. The detailed bioinformatic analysis of the *hmn* BCG was performed using the BLAST tool (Supplementary Materials, Table S2).

### 3.4. Extraction and Isolation

The fermentation broth (40 L) was subjected to three rounds of extraction with an equal volume of ethyl acetate (EtOAc) at room temperature. The resulting EtOAc layer was separated from the aqueous phase, and then evaporated in vacuo to obtain a dry EtOAc extract weighing 14.5 g. The EtOAc extract was initially separated on silica gel CC eluted with CH_2_Cl_2_/MeOH from 1:0 to 0:1 to afford seven subfractions, Fr.1–Fr.7. Fr.2 (7.8 g) was subjected to MPLC C-18 with gradient MeOH/H_2_O to yield Fr.2.1–Fr.2.11. Fr.2.4 (1.1 g) was chromatographed on a Sephadex LH-20 column eluted with MeOH to afford five subfractions, Fr.2.4.1–Fr.2.4.5. Subsequently, Fr.2.4.3 (201 mg) was separated on silica gel CC with gradient CH_2_Cl_2_/MeOH and further purified by semi-preparative HPLC to yield **1** (1.1 mg). Fr.2.4.2 (746 mg) was purified by semi-preparative HPLC to yield **5** (248 mg). Fr.2.3 (980 mg) was subjected to the Sephadex LH-20 column eluted with MeOH to afford four subfractions, Fr.2.3.1–Fr.2.3.4. Fr.2.3.2 (181 mg) was further purified by semi-preparative HPLC to afford **2** (0.8 mg) and **4** (5.9 mg). Fr.2.2 (440 mg) was chromatographed on the Sephadex LH-20 column eluted with MeOH to afford three subfractions, Fr.2.2.1–Fr.2.2.3. Fr.2.2.1 (35 mg) was further purified by semi-preparative HPLC to yield **3** (1.6 mg). Fr.2.6 (467 mg) was separated on the Sephadex LH-20 column eluted with MeOH to generate five subfractions, Fr.2.6.1–Fr.2.6.5. Fr.2.6.3 (102 mg) was chromatographed on silica gel CC eluted with gradient CH_2_Cl_2_/MeOH to afford six subfractions, Fr.2.6.3.1–Fr.2.6.3.6. Fr.2.6.3.3 (29 mg) was further purified by semi-preparative HPLC to afford **6** (3.1 mg) and **7** (1.6 mg). Additionally, the three pairs of enantiomers (**1**–**3**) were further separated on a chiral column (Lux^®^ 5 μm Cellulose-2, 250 × 4.6 mm, phenomenex) eluted with *n*-hexane/isopropanol to provide their monomers.

Compound **1**, white powder, HRESIMS *m*/*z* 235.0968 [M + H]^+^ (calcd for C_13_H_15_O_4_ 235.0965); UV (MeOH) *λ*_max_ (log *ε*) 212 (3.93) nm, 242 (3.86) nm, 250 (3.88) nm, and 290 (3.69) nm; IR (film) *ν*_max_ 3323, 2945, 2833, 2362, 1647, 1456, 1112, 1020, and 667 cm^−1^; ^1^H NMR (CD_3_OD, 700 MHz) and ^13^C NMR (CD_3_OD, 176 MHz), see Table 1 and Figures S8–S12 (Supplementary Materials); (−)-aloesol (12*R*-**1**, **1a**), ${\left[\alpha \right]}_{D}^{25}$ − 15.5 (c 0.06, MeOH); (+)-aloesol (12*S*-**1**, **1b**), ${\left[\alpha \right]}_{D}^{25}$ + 14.0 (c 0.02, MeOH); CD (MeOH) of **1a**: 223 nm (*Δε* = −1.13), 281 nm (Δ*ε* = −0.36); CD (MeOH) of **1b**: 223 nm (Δ*ε* = 0.79), 279 nm (Δ*ε* = 0.21).

Compound **2**, yellow oil, HRESIMS *m*/*z* 213.1136 [M − H]^−^ (calcd for C_11_H_17_O_4_ 213.1132); UV (MeOH) *λ*_max_ (log *ε*) 215 (4.15) nm; IR (film) *ν*_max_ 3334, 2945, 2833, 2362, 1697, 1647, 1417, 1112, 1020, and 669 cm^−1^; ^1^H NMR (CD_3_OD, 700 MHz) and ^13^C NMR (CD_3_OD, 176 MHz), see Table 1 and Figures S18–S23 (Supplementary Materials); (−)-6-hydroxy-2-methyl-8-oxodec-2-enoic acid (6*R*-**2**, **2a**), ${\left[\alpha \right]}_{D}^{25}$ − 9.3 (c 0.04, MeOH); (+)-6-hydroxy-2-methyl-8-oxodec-2*E*-enoic acid (6*S*-**2**, **2b**), ${\left[\alpha \right]}_{D}^{25}$ + 7.8 (c 0.03, MeOH); CD (MeOH) of **2a**: 215 nm (Δ*ε* = −0.1), 272 nm (Δ*ε* = −0.07).

Compound **3**, yellow oil, HRESIMS *m*/*z* 223.0945 [M **+** Na]**^+^** (calcd for C_10_H_16_O_4_Na 223.0941); UV (MeOH) *λ*_max_ (log *ε*) 215 (4.04) nm; IR (film) *ν*_max_ 3329, 2947, 2833, 2362, 1697, 1653, 1456, 1417, 1271, 1111, 1018, and 667 cm^−1^; ^1^H NMR (CD_3_OD, 700 MHz) and ^13^C NMR (CD_3_OD, 176 MHz), see Table 2 and Figures S28–S33 Supplementary Materials); (−)-6-hydroxy-2-methyl-8-oxonon-2*E*-enoic acid (6*R*-**3**, **3a**), ${\left[\alpha \right]}_{D}^{25}$ − 7.2 (c 0.04, MeOH); (+)-6-hydroxy-2-methyl-8-oxonon-2*E*-enoic acid (6*S*-**3**, **3b**), ${\left[\alpha \right]}_{D}^{25}$ + 9.5 (c 0.06, MeOH); CD (MeOH) of **3a**: 224 nm (Δ*ε* = −0.07), 284 nm (Δ*ε* = −0.04); CD (MeOH) of **3b**: 228 nm (Δ*ε* = 0.28), 281 nm (Δ*ε* = 0.23).

2-methyl-6-oxohept-2*E*-enoic acid (**4**), yellow oil, HRESIMS *m*/*z* 155.0707 [M − H]**^−^** (calcd for C_8_H_11_O_3_ 155.0714); UV (MeOH) *λ*_max_ (log *ε*) 215 (4.26) nm; IR (film) *ν*_max_ 3336, 2945, 2835, 2358, 1697, 1653, 1417, 1259, 1172, 1016, and 667 cm^−1^; ^1^H NMR (CD_3_OD, 500 MHz) and ^13^C NMR (CD_3_OD, 126 MHz), see Table 2 and Figures S39–S44 (Supplementary Materials).

Homononactic acid (**5**), white powder, ESIMS *m*/*z* 217.5 [M **+** H]**^+^**, C_11_H_20_O_4_, ${\left[\alpha \right]}_{D}^{25}$ + 3.9 (c 0.1, MeOH); CD (MeOH), 211 nm (Δ*ε* = 0.11); ^1^H NMR (CDCl_3_, 500 MHz) and ^13^C NMR (CDCl_3_, 126 MHz), see Figures S48–S49 (Supplementary Materials).

*trans*, *trans*-3, 7-dimethyl-2, 6-decadien-1, 10-dioic acid (**6**), white powder, ESIMS *m*/*z* 225.3 [M − H]**^−^**, C_12_H_18_O_4_, ^1^H NMR (CD_3_OD, 700 MHz) and ^13^C NMR (CD_3_OD, 176 MHz), see Figures S52 and S53 (Supplementary Materials).

*trans*, *trans*-10-hydroxy-3, 7-dimethyl-2, 6-decadienoic acid (**7**), white powder, ESIMS *m*/*z* 213.4 [M **+** H]**^+^**, C_12_H_20_O_3_, ^1^H NMR (CD_3_OD, 700 MHz) and ^13^C NMR (CD_3_OD, 176 MHz), see Figures S55 and S56 (Supplementary Materials).

Crystallographic data for the mixture of **1** were deposited in the Cambridge Crystallographic Data Centre (deposition number CCDC 2323933). Copies of the data can be obtained free of charge from the CCDC via www.ccdc.cam.ac.uk (accessed on 8 January 2024).

Crystal data for compound **1**: C_26_H_32_O_10_ (*M* = 504.51 g/mol), triclinic, space group P1 (no. 1), *a* = 8.5490(2) Å, *b* = 12.2335(3) Å, *c* = 12.7276(4) Å, *α* = 109.381(2)°, *β* = 90.219(2)°, *γ* = 93.556(2)°, *V* = 1252.84(6) Å^3^, *Z* = 2, *T* = 100.00(10) K, μ(Cu Kα) = 0.862 mm^−1^, *Dcalc* = 1.337 g/cm^3^, 26347 reflections measured (7.366° ≤ 2Θ ≤ 149.148°), 9427 unique (*R*_int_ = 0.0610, R_sigma_ = 0.0594), which were used in all calculations. The final *R*_1_ was 0.0671 (*I* > 2*σ*(*I*)) and *wR*_2_ was 0.1881 (all data). The flack parameter was 0.01(18).

### 3.5. Antibacterial Assay

The four bacteria (*Bacillus subtilis*, *Staphylococcus aureus*, *Vibrio alginolyticus*, *and Escherichia coli*) were used to measure antibacterial activity. The experimental procedure was the same as in our previous paper [16]. To each sterile filter paper disk instead of agar well, we added 5 μL of test compounds at a 1 mg/mL concentration and the solvent methanol as a negative control. Ciprofloxacin was used as a positive control.

### 3.6. DPPH Free Radical Scavenging Assay

The DPPH (2, 2-diphenyl-1-picrylhydrazyl) antioxidant assay was conducted with minor modifications of a previously reported method [17]. All tested compounds were diluted in MeOH to a concentration of 400 µg/mL. We added 100 µL of DPPH methanol solution (200 µM, final concentration = 100 µM) to the wells of 96-well plates containing 100 µL of the solution of the tested compounds. The mixtures were shaken and then kept in the dark at room temperature. After 30 min, the absorbance values were recorded at 517 nm using a K3 Plus microplate reader (BIO-DL), and the free radical scavenging ability was calculated as [1 − (*A*_sample_ − *A*_control_)/*A*_blank_] × 100%, where *A*_sample_, *A*_control_, and *A*_blank_ were the absorbance values for the wells containing test compounds + DPPH, test compounds + methanol, and methanol + DPPH, respectively. A mixture of ascorbic acid–methanol solution (100 µL, 120 µM) and DPPH solution (100 µL, 200 µM) was used as the positive control. The experiments were performed in triplicate, and the results were averaged.

### 3.7. LC-MS/MS Assay and Molecular Networking

A 30 μL EtOAc extract (25 mg/mL, dissolved in MeOH) was introduced into the LC-MS/MS system and eluted at a flow rate of 1 mL/min using a gradient program of CH_3_CN/H_2_O (with 0.1% ammonium hydroxide modifier): starting from 10% to 100% in 30 min, followed by 100% isocratic elution to 40 min, and then returning to 10% isocratic elution to 45 min. The separation was conducted on a YMC-Pack ODS-A analytical column (250 × 4.6 mm, S-5 μm), with a mass spectrometric detection set used to acquire data in the positive HRESI mode over *m*/*z* 50–1500, accompanied by automated, fully dependent MS/MS scans. Molecular networking was performed using the same method reported in our previous publication [16].

## 4. Conclusions

In conclusion, a detailed chemical investigation resulted in the isolation of seven compounds from the strain SCSIO 66814, including a new (−)-aloesol (**1a**), two pairs of enantiomers of fatty acid (**2** and **3**), and one new natural product (**4**). Compounds **2**–**5** were determined to be related precursors for nonactin and its homologs, and compound **5** is putatively formed through successive ketone reductive and intermolecular Michael addition reactions from compound **2**. Together with bioinformatic analysis, there findings led us to propose a plausible biosynthetic pathway for the formation of homononactic acid and nonactic acid. Additionally, biological assays showed that only (+)-**1** displayed a weak activity against DPPH radicals. While none of the isolated compounds exhibited obvious antibacterial activity, some of them presented a noncompetitive property according to the previously published literature. For example, (+)-aloesol (**1b**) displayed a QS *rnaIII-lacZ* inhibition with a fluorescence value of 11% at 32 μg/mL [18]. Compounds **6** and **7** were reported to be components of the pheromonal secretion of the male monarch butterfly. Furthermore, compound **5** inhibited BMI1 promoter activities with IC_50_ values of 240 μM [9], and some linear dimer ester and cyclic di- and trimers derived from **5** including bonactin [19] and di-/tri-lactones [20] displayed significant antimicrobial activity against both Gram-positive and Gram-negative bacteria as well as against fungi.

## Figures and Tables

**Table 1:** ^1^H and ^13^C NMR data of **1** and **2** in methanol-*d*_4_ (TMS, *δ* in ppm, *J* in Hz).

1 ^a^			2 ^a^		
Position	*δ*_H_	*δ*_C_	Position	*δ*_H_	*δ*_C_
2		167.1, s	1		172.4, s
3	6.06 (s)	112.5, d	2		130.0, s
4		182.0, s	3	6.75 (tq, 7.6, 1.6)	142.5, d
5		143.7, s	4	2.27 (m), 2.32 (m)	25.9, t
6	6.63 (d, 2.4)	118.2, d	5	1.56 (m)	37.2, t
7		163.5, s	6	4.04 (tt, 8.4, 4.4)	68.5, d
8	6.65 (d, 2.4)	101.8, d	7	2.58 (m)	50.7, t
9		161.6, s	8		212.9, s
10		115.8, s	9	2.51 (m)	37.6, t
11	2.65 (dd, 14.4, 7.9), 2.71 (dd, 14.4, 4.9)	44.3, t	10	1.01 (t, 7.3)	7.8, q
12	4.18 (dqd, 7.9, 6.2, 4.9)	66.4, d	11	1.82 (br s)	12.6, q
13	1.27 (d, 6.2)	23.2, q			
14	2.72 (s)	23.5, q			

^a 1^H and ^13^C NMR data were recorded at 700 MHz and 176 MHz, respectively.

**Table 2:** ^1^H and ^13^C NMR data of **3** and **4** in methanol-*d*_4_ (TMS, *δ* in ppm, *J* in Hz).

3 ^a^			4 ^b^		
Position	*δ*_H_	*δ*_C_	Position	*δ*_H_	*δ*_C_
1		173.0, s	1		171.6, s
2		130.5, s	2		129.8, s
3	6.72 (tq,7.5, 1.5)	142.0, d	3	6.74 (tq, 7.4, 1.5)	142.2, d
4	2.27 (m), 2.31 (m)	25.8, t	4	2.45 (m)	23.8, t
5,	1.56 (m)	37.2, t	5	2.68 (t, 7.2)	42.7, t
6	4.04 (m)	68.3, d	6		210.3, s
7	2.60 (d, 5.9)	51.8, t	7	2.18 (s)	29.8, q
8		210.6, s	8	1.86 (br s)	12.5, q
9	2.17 (s)	30.7, q			
10	1.82 (br s)	12.7, q			

^a 1^H and ^13^C NMR data were recorded at 700 MHz and 176 MHz, respectively; ^b 1^H and ^13^C NMR data were recorded at 500 MHz and 126 MHz, respectively.

**Table 3:** DPPH free radical scavenging activities of **1**–**7**.

Compounds ^a^	Scavenging Rate%	Compounds ^a^	Scavenging Rate%
(+)-**1**	34.42%	**4**	6.54%
(−)-**1**	16.04%	**5**	3.04%
(+)-**2**	7.01%	**6**	6.11%
(−)-**2**	16.20%	**7**	8.87%
(+)-**3**	10.51%	Ascorbic acid ^b^	96.89%
(−)-**3**	15.58%		

^a^ The final concentration of the tested compounds was 200 µg/mL; ^b^ the final concentration of ascorbic acid as the positive control was 10.6 µg/mL.

## Data Availability

The data presented in this study are available in this article and the Supplementary Materials, further inquiries can be directed to the corresponding author.

## Supplementary Materials

The following supporting information can be downloaded at https://www.mdpi.com/article/10.3390/md22040181/s1: General experimental procedure; calculated method, 1D/2D NMR, HRESIMS, UV, and IR spectra of **1**−**4**; 1D/2D NMR and ESIMS spectra of **5**–**8**; information of 57 biosynthetic gene clusters; annotations of *hmn* biosynthetic gene cluster; molecular networking; LC-MS/MS profiles; chiral HPLC analysis for **1**–**3**.
