# Supplementary material for: Discovery of a Novel Chromone Enantiomer and the Precursors of Nonactic Acid from the Coral-Reef-Derived Streptomyces sp. SCSIO 66814
Source: Mar Drugs. 2024 Apr 17;22(4):181. doi: 10.3390/md22040181 (PMC11051294; doi:10.3390/md22040181)
Supplement: Supplementary file 1 [file marinedrugs-22-00181-s001.zip › marinedrugs-2948086-supplementary.pdf]

Discovery of a Novel Chromone Enantiomer and the Precursors of Nonactic Acid from the Coral-Reef-Derived *Streptomyces* sp. SCSIO 66814

Wenping Ding [1,#], Yanqun Li [1,#], Xingyu Li [1], Jiajia Yin [1], Songbiao Shi [1], Xinpeng Tian [1], Si Zhang [1,2,*] and Hao Yin [1,2,*]

[1] *CAS Key Laboratory of Tropical Marine Bio-resources and Ecology, South China Sea Institute of Oceanology, Chinese Academy of Sciences, Guangzhou 510301, China*

[2] *Southern Marine Science and Engineering Guangdong Laboratory (Guangzhou), Guangzhou 511458, China*

[#] *These authors contributed equally to this work*

Corresponding authors

zhsimd@scsio.ac.cn (S.Z.)
yinhao@scsio.ac.cn (H.Y.)

Contents

General experimental procedure 3

Calculated method 3

Figure S1. Chiral HPLC analysis profiles of compound 1 at 240nm 4

Figure S2. Chiral HPLC analysis profiles of compound 2 at 230nm 5

Figure S3. Chiral HPLC analysis profiles of compound 3 at 230nm 5

Table S1. Predicted 57 biosynthetic gene clusters using antiSMASH with a “loose” detection strictness setting 6

Table S2. Predicted function of the open reading frames 7

Figure S4. Integrated cluster-node diagram of Molecular Networking 9

Table S3. The chemical structures being annotated by GNPS 9

Figure S5. LC-MS/MS profiles at 37.8 min, 39.4 min, and 42.2 min 10

Table S4. The three compounds being confirmed by LC-MS/MS 10

Figure S6. The MS/MS cleavage fragments from nonactin 11

Figure S7. HRESIMS spectrum of compound 1 11

Figure S8. ^{1}H NMR spectrum (CD_3OD, 700 MHz) of compound 1 12

Figure S9. ^{13}C NMR and DEPT spectra (CD_3OD, 176 MHz) of compound 1 14

Figure S10. HSQC spectrum of compound 1 14

Figure S11. HMBC spectrum of compound 1 15

Figure S12. ^1H–^1H COSY spectrum of compound 1 15
Figure S13. UV spectrum of compound 1 16
Figure S14. IR spectrum of compound 1 16
Figure S15. CD spectrum of compound 1a 17
Figure S16. CD spectrum of compound 1b 17
Figure S17. HRESIMS spectrum of compound 2 18
Figure S18. ^1H NMR spectrum (CD_3OD, 700 MHz) of compound 2 18
Figure S19. ^{13}C NMR and DEPT spectra (CD_3OD, 176 MHz) of compound 2 20
Figure S20. HSQC spectrum of compound 2 21
Figure S21. HMBC spectrum of compound 2 21
Figure S22. ^1H–^1H COSY spectrum of compound 2 22
Figure S23. NOESY spectrum of compound 2 22
Figure S24. UV spectrum of compound 2 23
Figure S25. IR spectrum of compound 2 23
Figure S26. CD spectrum of compound 2a 24
Figure S27. HRESIMS spectrum of compound 3 24
Figure S28. ^1H NMR spectrum (CD_3OD, 700 MHz) of compound 3 25
Figure S29. ^{13}C NMR and DEPT spectra (CD_3OD, 176 MHz) of compound 3 26
Figure S30. HSQC spectrum of compound 3 27
Figure S31. HMBC spectrum of compound 3 27
Figure S32. ^1H–^1H COSY spectrum of compound 3 28
Figure S33. NOESY spectrum of compound 3 28
Figure S34. UV spectrum of compound 3 29
Figure S35. IR spectrum of compound 3 29
Figure S36. CD spectrum of compound 3a 30
Figure S37. CD spectrum of compound 3b 30
Figure S38. HRESIMS spectrum of compound 4 31
Figure S39. ^1H NMR spectrum (CD_3OD, 500 MHz) of compound 4 31
Figure S40. ^{13}C NMR and DEPT spectra (CD_3OD, 126 MHz) of compound 4 33
Figure S41. HSQC spectrum of compound 4 33
Figure S42. HMBC spectrum of compound 4 34
Figure S43 ^1H–^1H COSY spectrum of compound 4 34

Figure S44. NOESY spectrum of compound 4 35
Figure S45. UV spectrum of compound 4 35
Figure S46. IR spectrum of compound 4 36
Figure S47. ESIMS spectrum of compound 5 36
Figure S48. ^{1}H NMR spectrum ($CDCl_3$, 500 MHz) of compound 5 37
Figure S49. ^{13}C NMR and DEPT spectra ($CDCl_3$, 126 MHz) of compound 5 38
Figure S50. CD spectrum of compound 5 39
Figure S51. ESIMS spectrum of compound 6 39
Figure S52. ^{1}H NMR spectrum (CD_3OD, 700 MHz) of compound 6 40
Figure S53. ^{13}C NMR and DEPT spectra (CD_3OD, 176 MHz) of compound 6 41
Figure S54. ESIMS spectrum of compound 7 42
Figure S55. ^{1}H NMR spectrum (CD_3OD, 700 MHz) of compound 7 42
Figure S56. ^{13}C NMR and DEPT spectra (CD_3OD, 176 MHz) of compound 7 44
Calculated the specific rotations of 2a and 2b 44

General experimental procedure

Column chromatography (CC): Claricep Flash C-18 Column (20–40 μm, Agela Technologies, Tianjin, China), Sephadex LH-20 (100–200 μm, Pharmacia, Uppsala, Sweden). HRESIMS was measured on a Bruker maXis quadrupole-time-of-flight mass spectrometer (Bruker, Billerica, USA). UV spectra were run on a UV-2600 spectrophotometer (Shimadzu, Kyoto, Japan). IR Spectra were performed on an IR Affinity-1 spectrometer (Shimadzu, Kyoto, Japan). 1D and 2D NMR spectra were recorded on Bruker AV500 or Bruker AVANCE III HD 700 MHz digital NMR spectrometer (Bruker Switzerland AG, Billerica, MA, USA) using TMS as the internal standard. MPLC (CHEETAH MP 200, Agela Technologies, Tianjin, China) was equipped with Flash C-18 Column (Agela Technologies, Tianjin, China). HPLC (Agilent 1260) was equipped with YMC-Pack ODS-A (250 × 4.6 mm or 250 × 10.0 mm, 5 μm, YMC, Ishikawa-ken, Japan) column, YMC-Pack Ph (250 × 4.6 mm or 250 × 10.0 mm, 5 μm, YMC, Ishikawa-ken, Japan) column. Fractions were monitored by thin layer chromatography, and spots were visualized by UV light (254 nm and 365 nm) and by smoking silica gel plates with iodine. All solvents used in CC and HPLC were of analytical grade (Tianjin Damao Chemical Plant, Tianjin, China) and chromatographic grade (MREDA), respectively.

Calculated method

The conformational search was implemented in xtb software package using molecular dynamic simulations with the method of 100 ps/400 K/GNF0.[1] The obtained 2000 conformers were sequentially optimized on with semi-empirical level of GNF0-xTB

and GNF2-xTB, and the conformers were sorted by Molclus 1.9.9.9 program.[2] The conformers within an energy window of 5 kcal/mol were subjected to further re-optimization and frequency calculations using the DFT method at B3LYP-D3(BJ)/6-31G* (IEFPCM, MeOH) level of theory. The specific rotation was calculated at 589 nm using B3LYP/6-311++G** (IEFPCM, MeOH) level of theory. The contribution of each conformer was weighted using Boltzmann averaging. All calculations were performed using Gaussian 16 program.

Reference

1. Grimme, S. Exploration of chemical compound, conformer, and reaction space with meta-dynamics simulations based on tight-binding quantum chemical calculations. *J. Chem. Theory Comput.* **2019**, 15 (5), 2847-2862.

2. Lu, T. Molclus program, Version 1.9.9.9, http://www.keinsci.com/research/molclus.html (accessed Mar-6, **2022**).

Figure S1. Chiral HPLC analysis profiles of compound **1** at 240nm

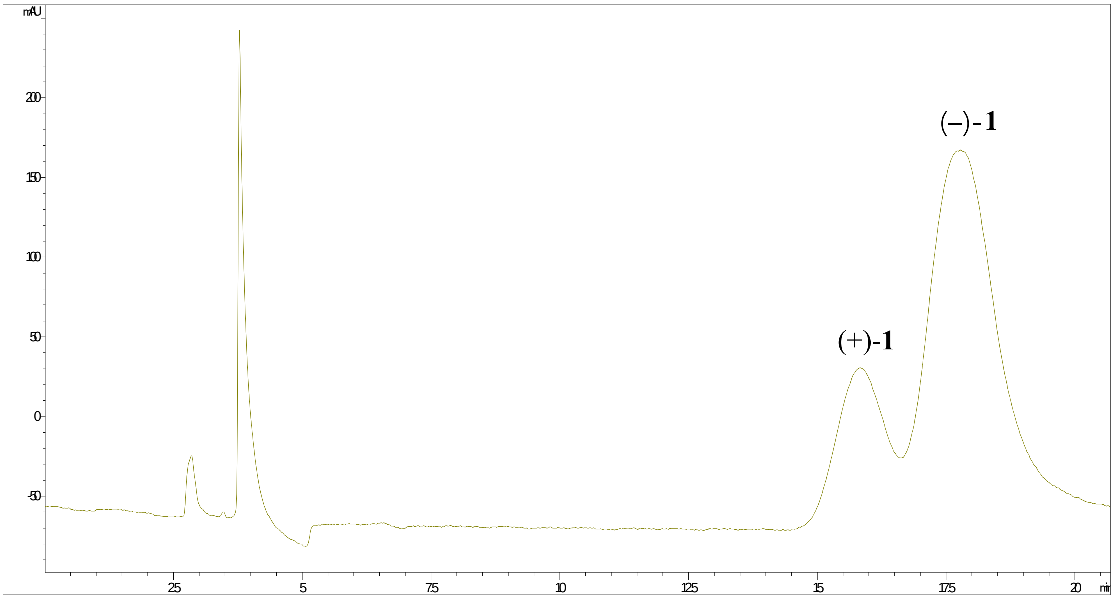

Note: PHENOMENEX chiral column eluted with *n*-hexane:isopropanol=81:19 containing 1% formic acid.

Figure S2. Chiral HPLC analysis profiles of compound **2** at 230nm

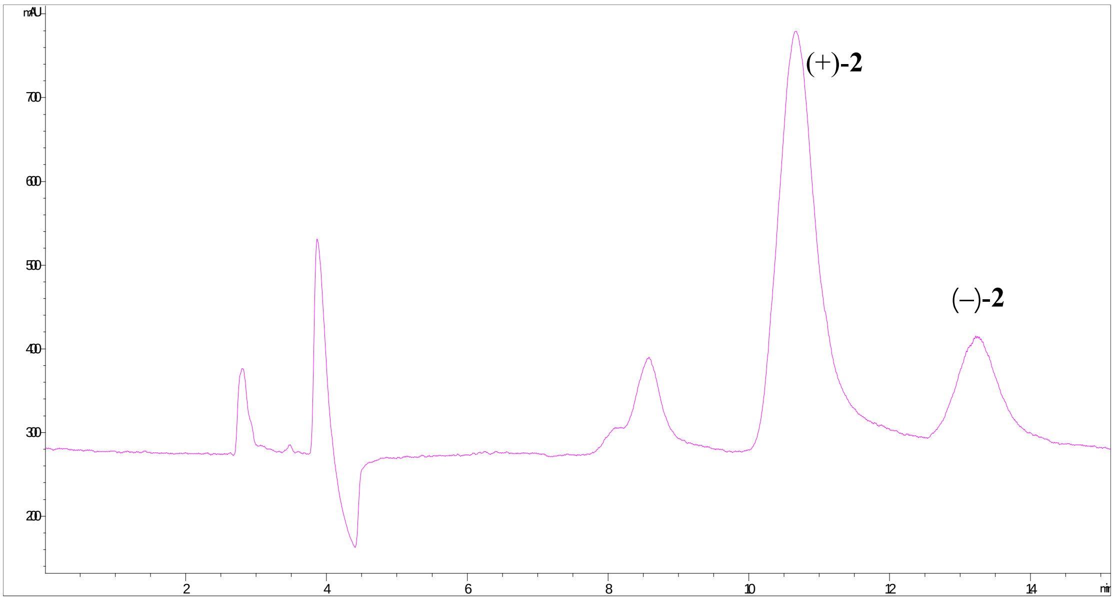

Note: PHENOMENEX chiral column eluted with *n*-hexane:isopropanol=85:15 containing 1% formic acid.

Figure S3. Chiral HPLC analysis profiles of compound **3** at 230nm

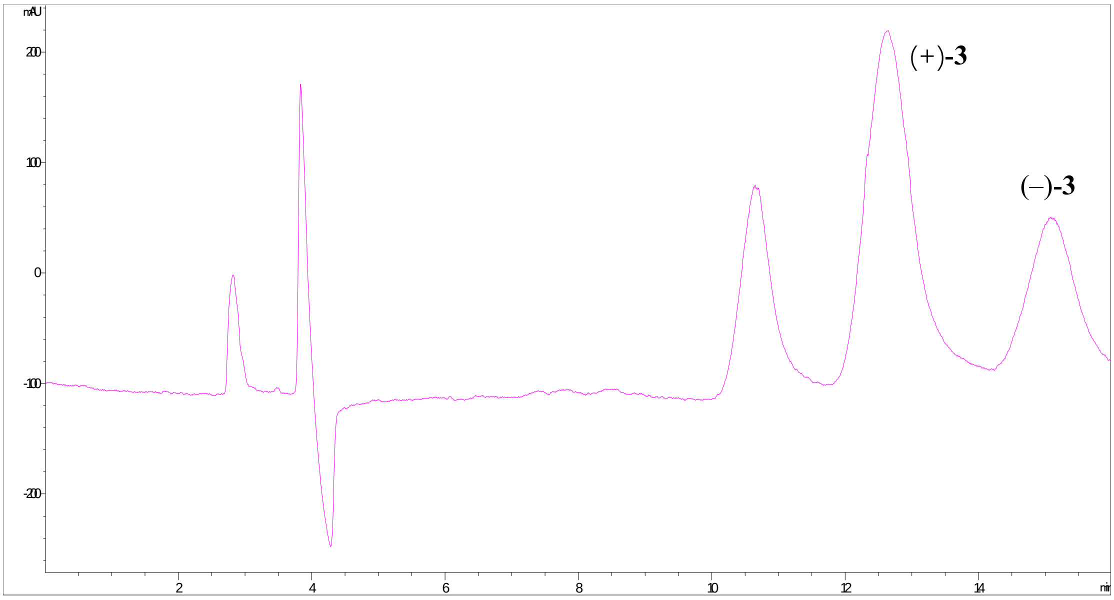

Note: PHENOMENEX chiral column eluted with *n*-hexane:isopropanol=85:15 containing 1% formic acid.

Table S1. Predicted 57 biosynthetic gene clusters using antiSMASH with a "loose" detection strictness setting

Region	Type	From	To	Most similar known cluster	Similarity
Region 1	betalactone	178212	205992	divergolide A/divergolide B/divergolide C/divergolide D	6%
Region 2	saccharide	212614	233384	murayaquinone	10%
Region 3	fatty_acid	268209	290590	metatricycloene	6%
Region 4	Saccharide, butyrolactone, ectoine	302298	364308	showdomycin	52%
Region 5	saccharide	427230	448195		
Region 6	NRPS	723200	777692	phosphonoglycans	3%
Region 7	saccharide	829632	860069	avilamycin A/avilamycin C	5%
Region 8	saccharide	965020	986000		
Region 9	RRE-containing, thiopeptide,LAP	1005575	1040402		
Region 10	NRPS-like	1277951	1321919	bottromycin A2	39%
Region 11	NI-siderophore	1390629	1420407	desferrioxamin B	100%
Region 12	fatty_acid	1471632	1496174	undecylprodigiosin/metacycloprodigiosin	8%
Region 13	lanthipeptide-class-iii, lanthipeptide-class-ii	1506504	1538114		
Region 14	saccharide	1683206	1722466	phosphonoglycans	10%
Region 15	saccharide	1775460	1799098	desulfoclethramycin/clethramycin	4%
Region 16	fatty_acid	1870427	1891458	tetrachlorizine	9%
Region 17	saccharide	2091402	2121764	acarviostatin I03/acarviostatin II03/ acarviostatin III03/acarviostatin IV03	33%
Region 18	saccharide	2242247	2267229		
Region 19	ectoine	2519550	2529948	ectoine	100%
Region 20	terpene	2982568	3003662	steffimycin D	19%
Region 21	halogenated	3418102	3439622	deoxyhangtaimycin	5%
Region 22	T3PKS, fatty_acid	3549573	3601750	undecylprodigiosin	9%
Region 23	T3PKS, NRPS, saccharide	3661610	3742004	diisonitrile antibiotic SF2768	22%
Region 24	terpene	3807192	3828202		
Region 25	NRP-metallophore, NRPS, melanin	3865798	3924518	coelichelin	81%
Region 26	NRP-metallophore, NRPS, transAT-PKS, T1PKS, PKS-like	3939564	4055936	griseobactin	100%
Region 27	terpene	4071873	4094086	geosmin	100%
Region 28	butyrolactone	4121314	4132183	coelimycin P1	16%
Region 29	arylpolyene, NRPS-like	4141349	4184539	o-dialkylbenzene 1/o-dialkylbenzene 2	12%
Region 30	T1PKS, NRPS, RRE-containing,	4197341	4250231	lactazole	33%

	thiopeptide,LAP				
Region 31	terpene	4262735	4288320	isorenieratene	100%
Region 32	NRPS, T3PKS	4397588	4514765	alkylresorcinol	100%
Region 33	saccharide, melanin,RiPP-like, NRPS, fatty_acid	4519368	4641892	valinomycin/montanastatin	91%
Region 34	NRPS	4697180	4764404	crochelin A	16%
Region 35	RiPP-like, T1PKS, NRPS	4807159	4859201	10-epi-HSAF/10-epi-3-deOH-HSAF/10-epi-maltophilin/10-epi-xanthobaccin C/10-epi-hydroxymaltophilin/10-epi-FI-2	100%
Region 36	T1PKS	4896322	4995821	bafilomycin B1	100%
Region 37	fatty_acid, thioamide-NRP	4996595	5061348	cadaside A/cadaside B	19%
Region 38	terpene, saccharide	5094369	5129744	hopene	69%
Region 39	fatty_acid	5287226	5308185		
Region 40	fatty_acid	5377036	5405230	nonactin/monactin/dinactin/trinactin/tetranactin	100%
Region 41	NRPS, NRPS-like, fatty_acid	5540515	5602480	asukamycin	11%
Region 42	saccharide	5662307	5693400	conglobatin	15%
Region 43	RiPP-like	5726575	5737981		
Region 44	saccharide	5792203	5827170	glycopeptidolipid	5%
Region 45	NRPS, hydrogen-cyanide	5839751	5887069	leucomycin	11%
Region 46	T2PKS, saccharide	5949123	6021614	medermycin	75%
Region 47	NI-siderophore	6120370	6153156	kinamycin	16%
Region 48	terpene, saccharide	6536320	6571261	stambomycin A/stambomycin B/stambomycin C/stambomycin D	16%
Region 49	saccharide	6574163	6602087	lavendiol	6%
Region 50	saccharide	6655576	6678253		
Region 51	saccharide	6844119	6868608		
Region 52	lanthipeptide-class-iii	6896866	6919592	AmfS	100%
Region 53	saccharide	7087152	7115500	notonesomycin A	6%
Region 54	halogenated	7526955	7548238	colibrimycin	17%
Region 55	lanthipeptide-class-i	7674268	7699536		
Region 56	lassopeptide	7830235	7852977	keywimysin	100%
Region 57	T2PKS, halogenated	7893896	7966387	maduralactomycin A/maduralactomycin B/ actinospirol A/actinospirol B	63%

Table S2. Predicted function of the open reading frames

Protein	Amino acids	Putative function	Nearest homologue (enzyme, origin)	Identity /similarity [%]	Accession number
ORF7	179	*O*-acetyl-ADP-ribose deacetylase	O-acetyl-ADP-ribose deacetylase [*Streptomyces* sp. DvalAA-19]	99%/100%	WP_093752860.1

ORF6	483	threonine/serine exporter	threonine/serine exporter family protein [*Streptomyces* sp. CAI-24]	98%/98%	WP_309236270.1
ORF5	472	NAD(P)/FAD-dependent oxidoreductase	NAD(P)/FAD-dependent oxidoreductase [*Streptomyces* sp. YIM 132580]	96%/97%	WP_202418510.1
ORF4	282	inositol monophosphatase	inositol monophosphatase family protein [*Streptomyces* sp. S8]	97%/97%	WP_084991105.1
ORF3	207	transcriptional regulator	response regulator transcription factor [*Streptomyces* sp. B27]	99%/99%	WP_127465383.1
ORF2	292	enoyl-CoA hydratase	enoyl-CoA hydratase/isomerase family protein [*Streptomyces* sp. CFMR 7]	96%/96%	WP_053558551.1
ORF1	319	ABC transporter ATP-binding protein	ABC transporter ATP-binding protein [*Streptomyces* sp. CS090A]	92%/96%	WP_109980241.1
HmnA	269	ABC transporter permease subunit	ABC transporter permease subunit [*Streptomyces* sp. ZL-24]	99%/99%	WP_103419230.1
HmnB	431	beta-ketoacyl synthase	beta-ketoacyl synthase [*Streptomyces* sp. S8]	96%/97%	ARI56317.1
HmnC	582	CoA-transferase	CoA-transferase [*Streptomyces* sp. CAI-24]	99%/99%	WP_175451846.1
HmnD	349	ketoacyl-ACP synthase III	ketoacyl-ACP synthase III [*Streptomyces* sp. SID8374]	97%/98%	WP_161281099.1
HmnE	323	ketoacyl-ACP synthase III	ketoacyl-ACP synthase III [*Streptomyces* sp. S6]	98%/99%	QCW79981.1
HmnF	305	3-ketoacyl-ACP reductase	3-ketoacyl-ACP reductase [*Streptomyces griseus* subsp. *griseus*]	88%/91%	TVP34093.1
HmnG	275	short-chain dehydrogenase	short-chain dehydrogenase [*Streptomyces griseus* subsp. *griseus*]	97%/98%	TVP34092.1
HmnH	453	beta-ketoacyl synthase	NonK [*Streptomyces griseus* subsp. *griseus*]	94%/96%	AAD37450.1
HmnI	427	beta-ketoacyl synthase	3-oxoacyl-ACP synthase [*Streptomyces griseus* subsp. *griseus*]	91%/95%	TVP34090.1
HmnJ	523	SDR family oxidoreductase	putative ketoacyl reductase [*Streptomyces griseus* subsp. *griseus*]	83%/86%	AAD37452.1
HmnK	272	SDR family oxidoreductase	acetoacetyl-CoA reductase [*Streptomyces griseus* subsp. *griseus*]	87%/91%	TVP34088.1
HmnL	567	CocE/NonD family hydrolase	X-Pro dipeptidyl-peptidase [*Streptomyces griseus* subsp. *griseus*]	87%/91%	TVP34087.1
HmnM	562	ATP-dependent acyl-CoA ligase	ATP-dependent acyl-CoA ligase [*Streptomyces* sp. CFMR 7]	99%/99%	WP_053558561.1
HmnN	301	enoyl-CoA hydratase	NonS, nonactate synthase [*Streptomyces griseus* subsp. *griseus*]	90%/92%	AAC26135.1"
HmnO	292	alpha/beta hydrolase	tetranactin resistance protein [*Streptomyces griseus* subsp. *griseus*]	88%/92%	AAD37454.1

HmnP	187	ArsR family transcriptional regulator	ArsR family transcriptional regulator [*Streptomyces* sp. S6]	98%/97%	QCW79977.1

Figure S4. Integrated cluster-node diagram of Molecular Networking

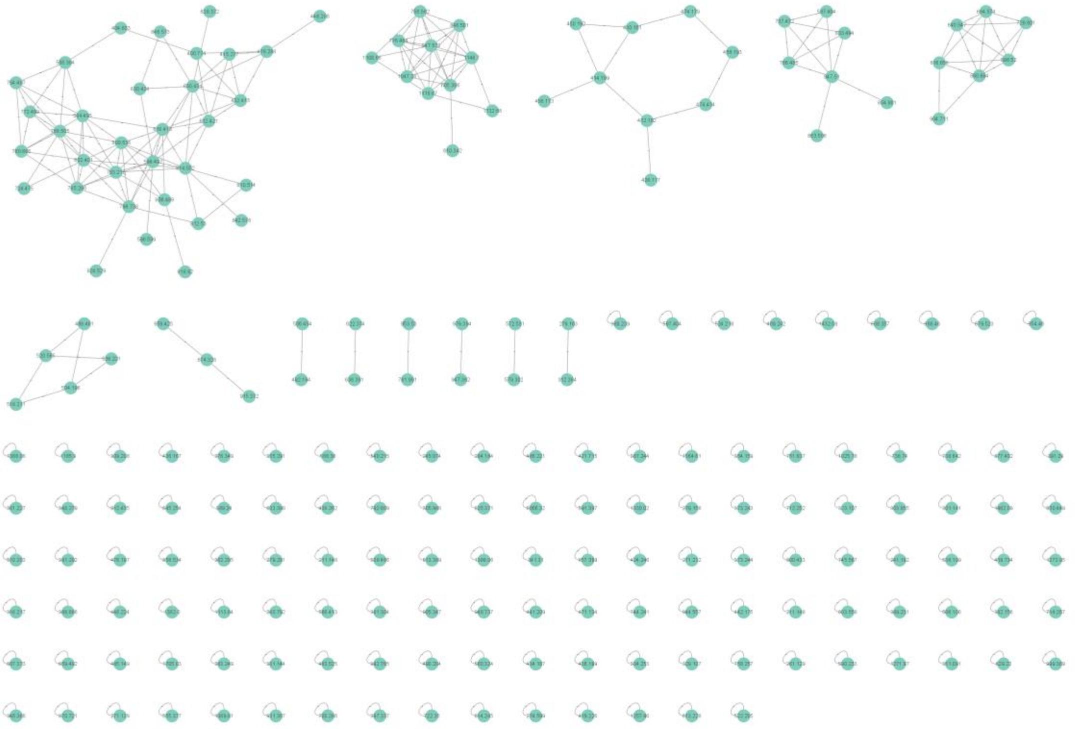

Note: The numbers on nodes represent parent ions.

Table S3. The chemical structures being annotated by GNPS

Parent ion	Compound name	Compound structure
785.286	Compound NP-002862 [a]	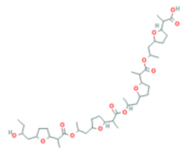
768.505	Monactin	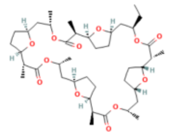
432.415	Compound NP-002857 [a]	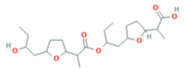
418.288	Bonactin	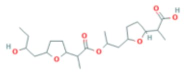

404.833	Compound NP-003108 [a]	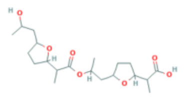
400.774	Bonactin	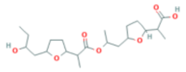

[a] PubChem name

Figure S5. LC-MS/MS profiles at 37.8 min, 39.4 min, and 42.2 min

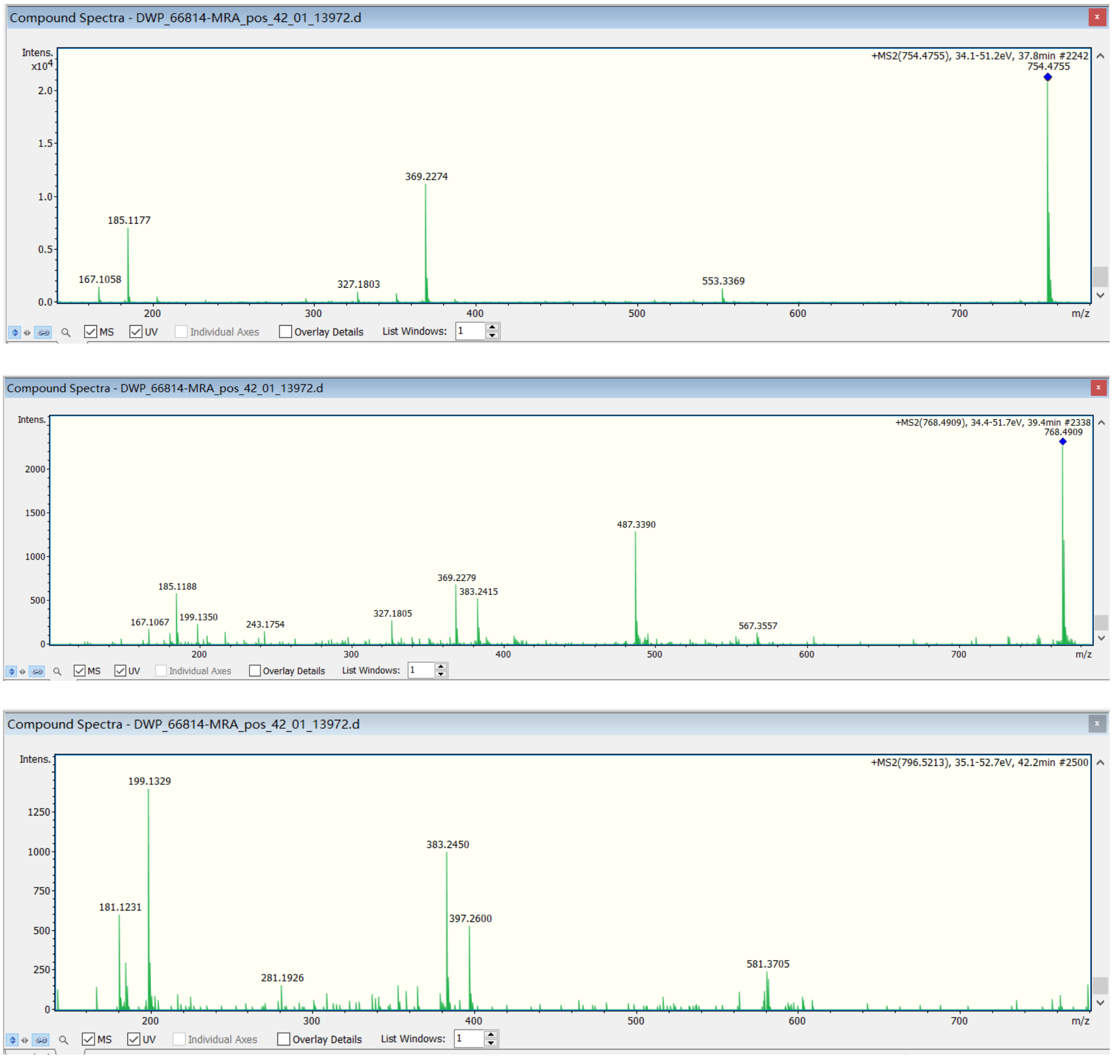

Table S4. The three compounds being confirmed by LC-MS/MS

Number	Compounds	Adduct	Molecular formula	Measure *m/z*	Calculated *m/z*	t_R (min)
1	nonactin	$[M + NH_4]^+$	$C_{40}H_{68}NO_{12}$	754.4755	754.4736	37.8

2	monactin	$[M + NH_4]^+$	$C_{41}H_{70}NO_{12}$	768.4909	768.4893	39.4
3	trinatin	$[M + NH_4]^+$	$C_{43}H_{74}NO_{12}$	796.5213	796.5206	42.2

Figure S6. The MS/MS cleavage fragments from nonactin

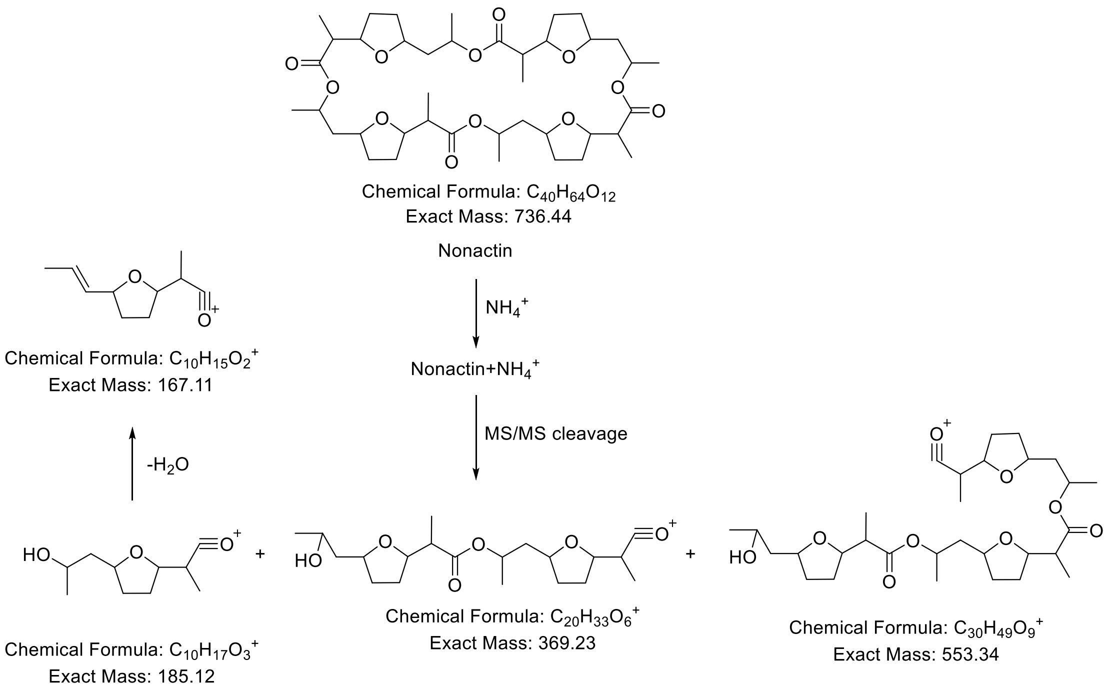

Figure S7. HRESIMS spectrum of compound **1**

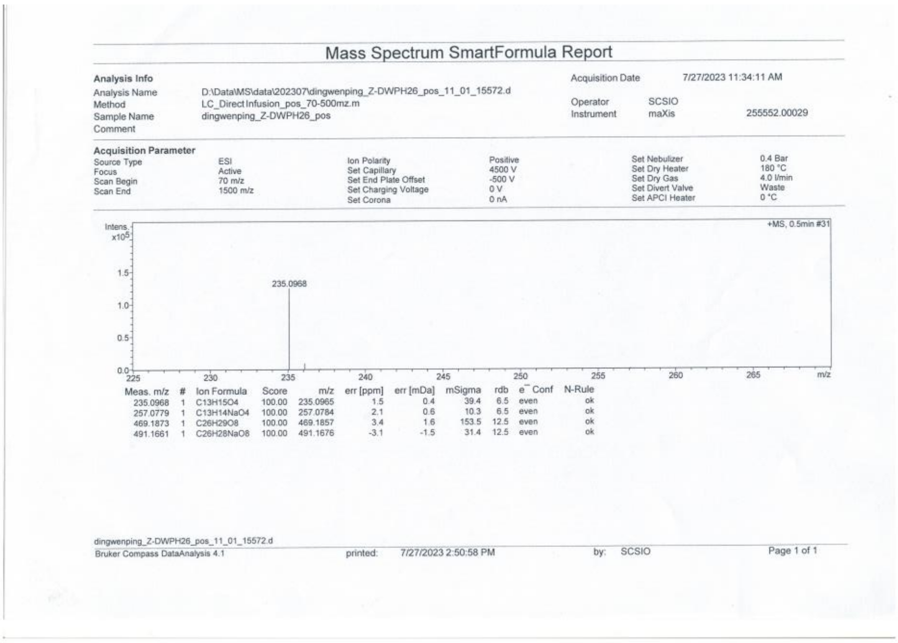

Figure S8. ^{1}H NMR spectrum (CD_3OD, 700 MHz) of compound **1**

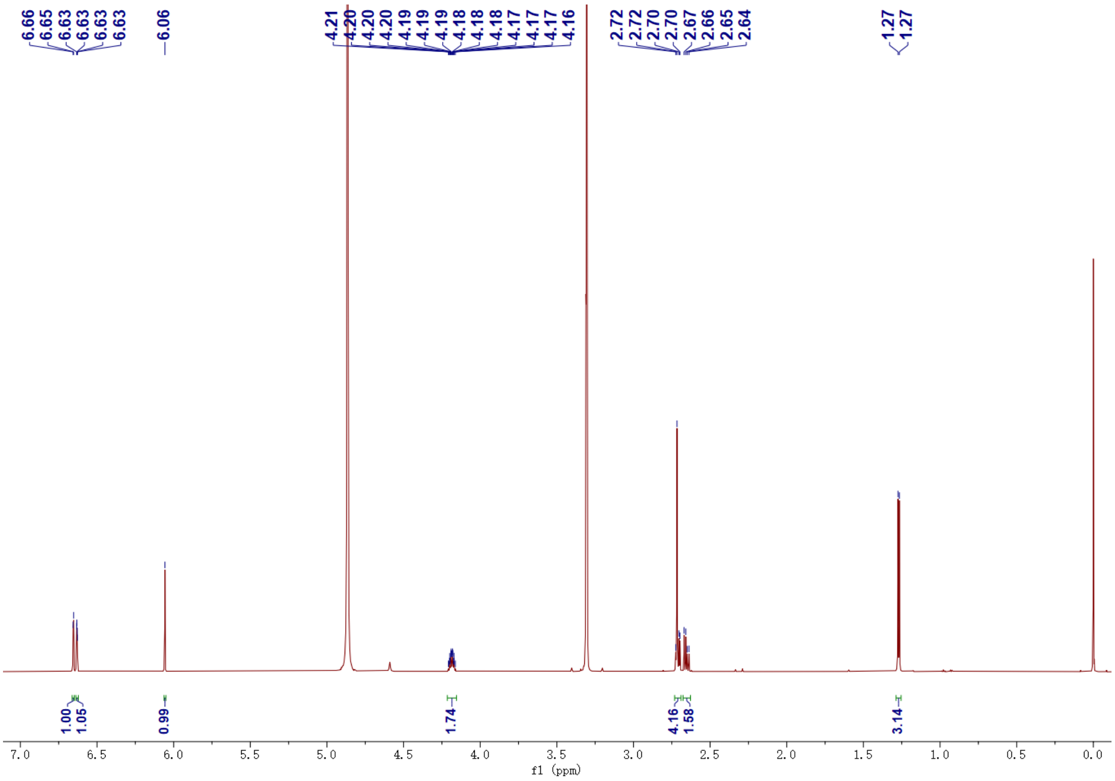

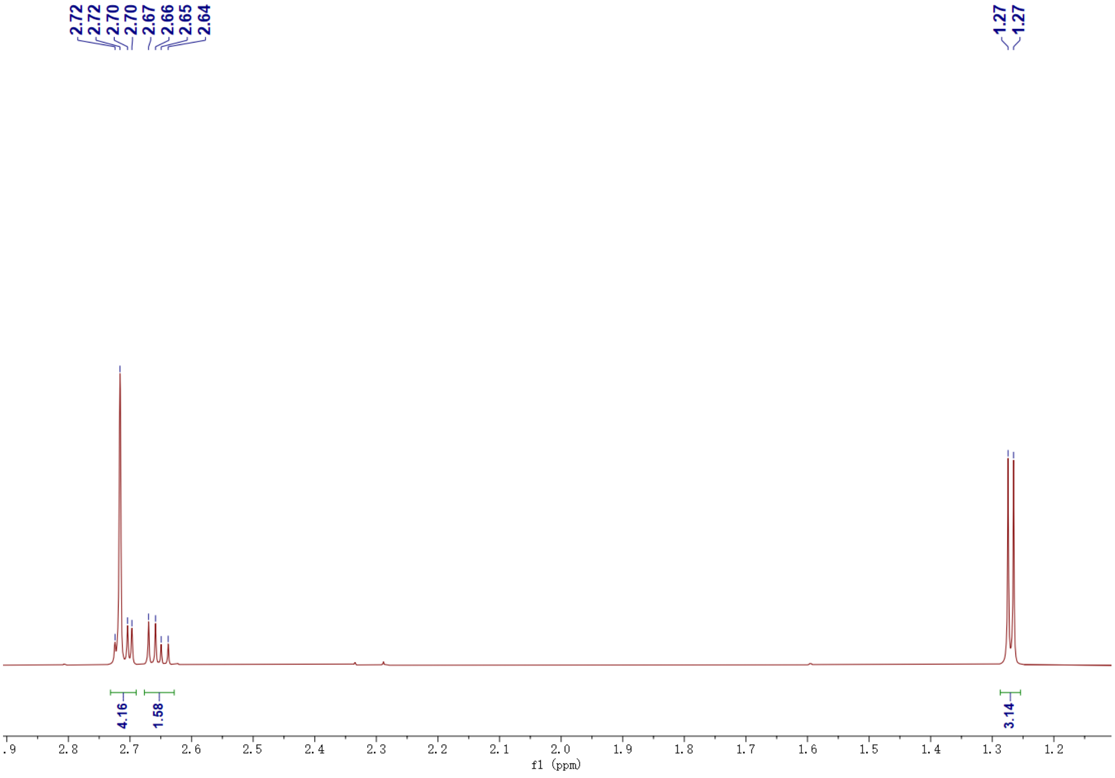

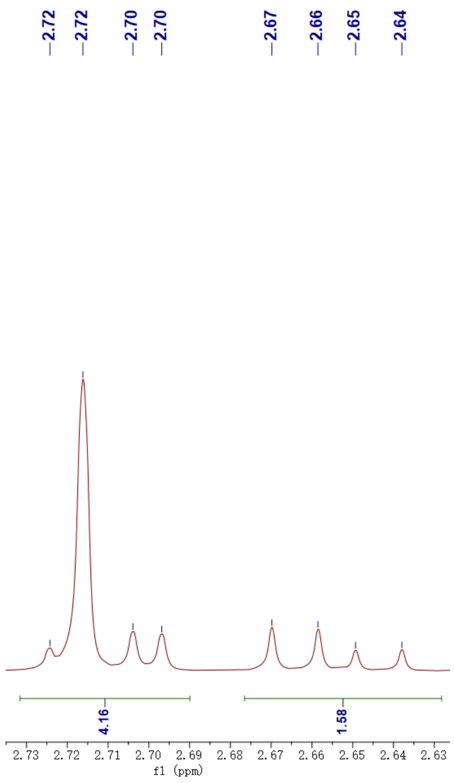
2.72
2.72
2.70
2.70
2.67
2.66
2.65
2.64
4.16
1.58
f1 (ppm)

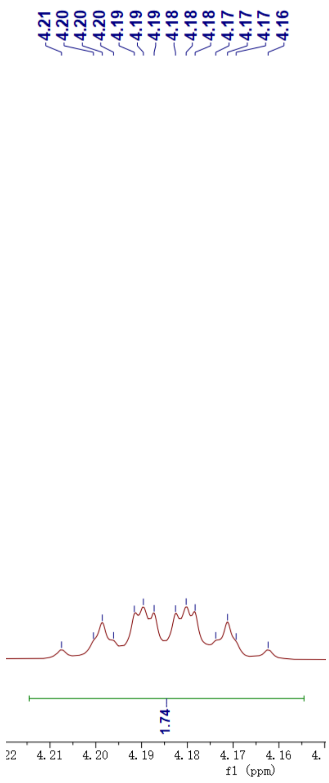
4.21
4.20
4.20
4.20
4.19
4.19
4.19
4.18
4.18
4.18
4.17
4.17
4.17
4.16
1.74
f1 (ppm)

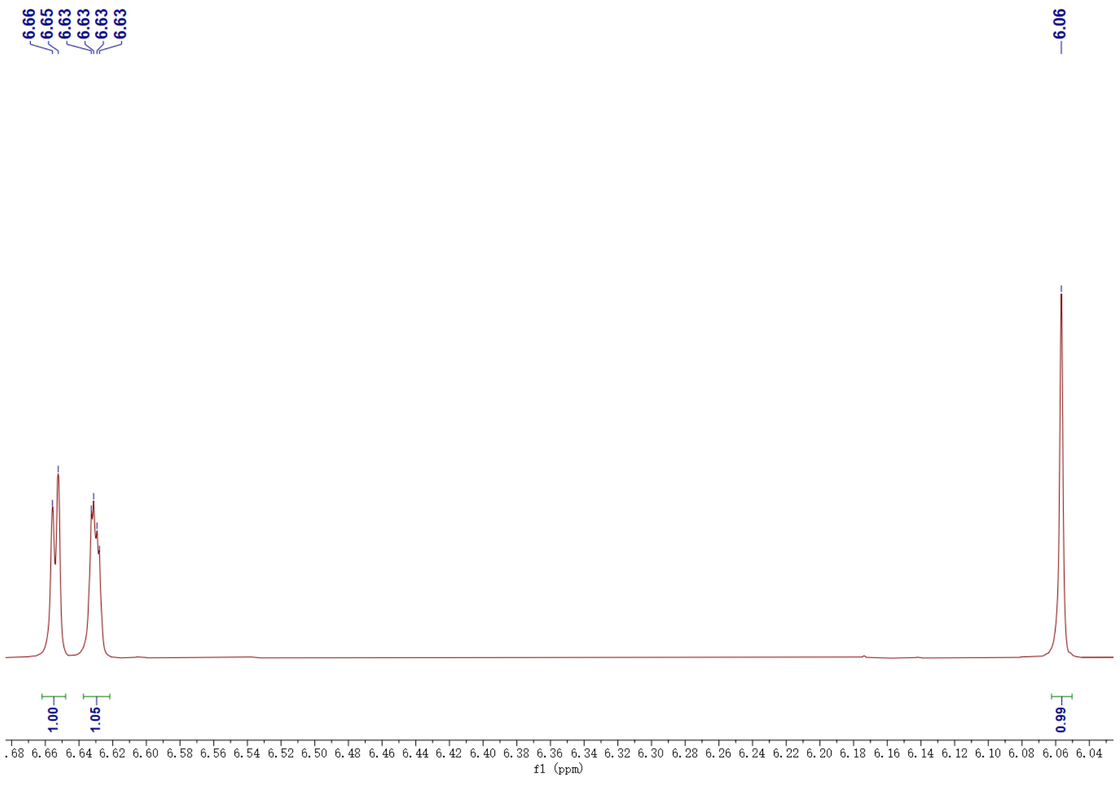
6.66
6.65
6.63
6.63
6.63
6.63
6.06
1.00
1.05
0.99
f1 (ppm)

Figure S9. ^{13}C NMR and DEPT spectra (CD3OD, 176 MHz) of compound **1**

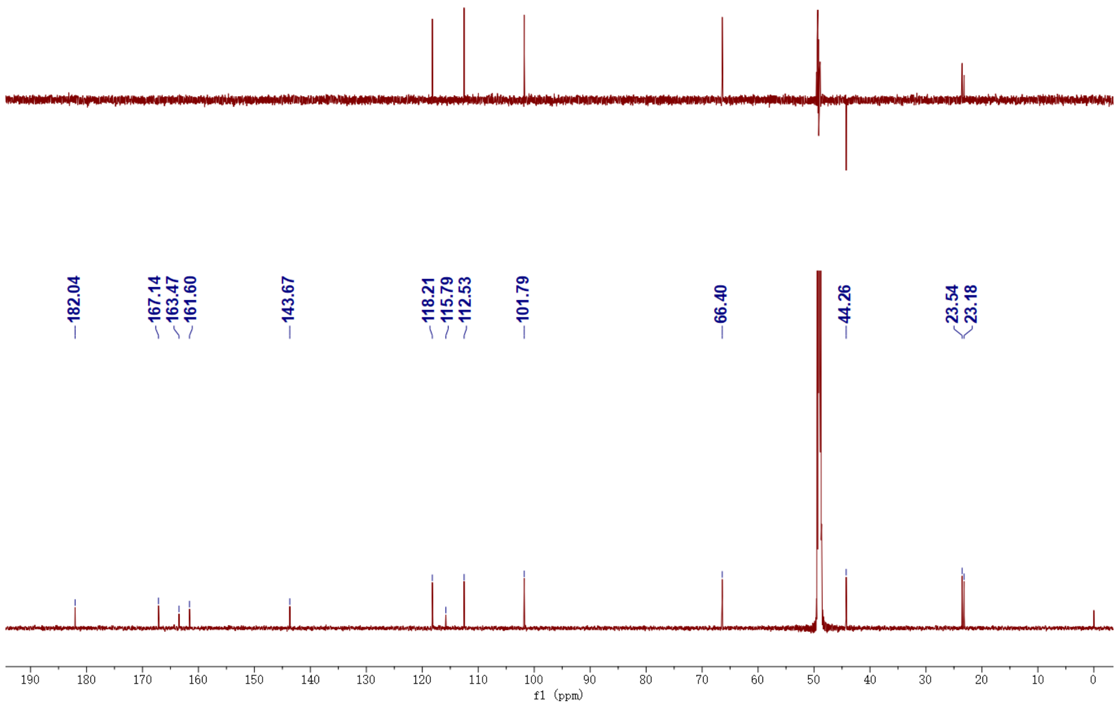

Figure S10. HSQC spectrum of compound **1**

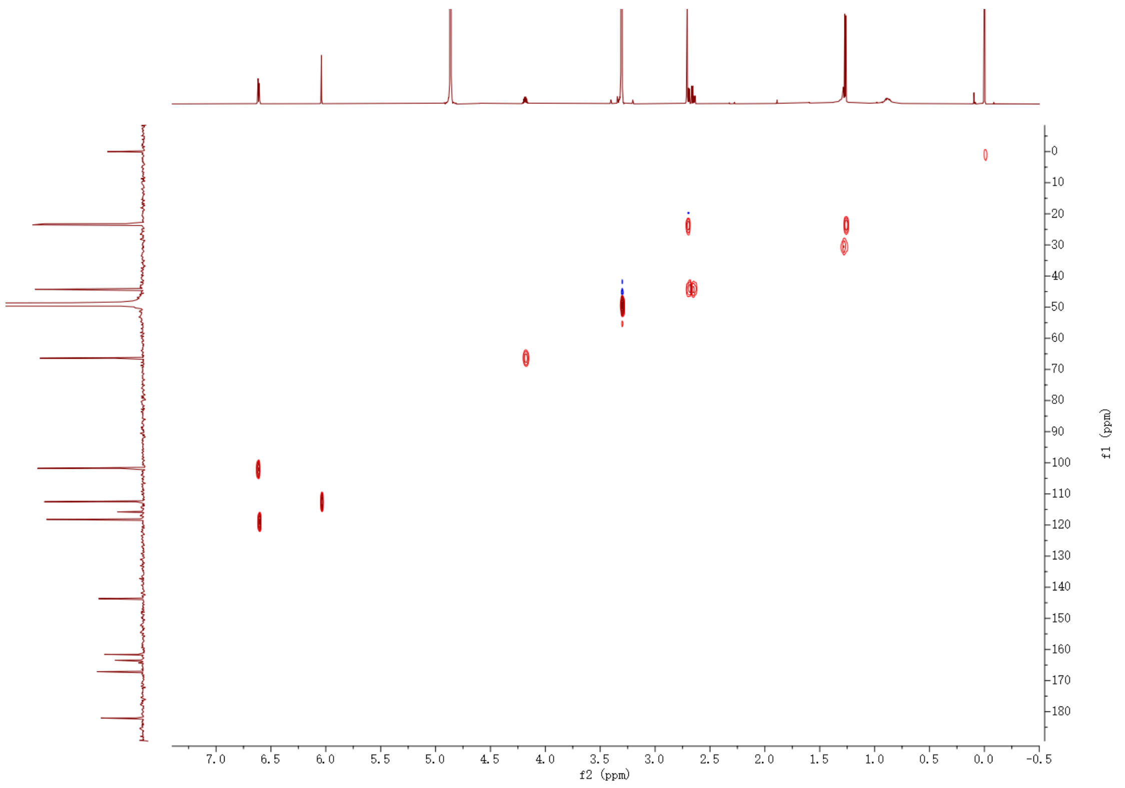

Figure S11. HMBC spectrum of compound **1**

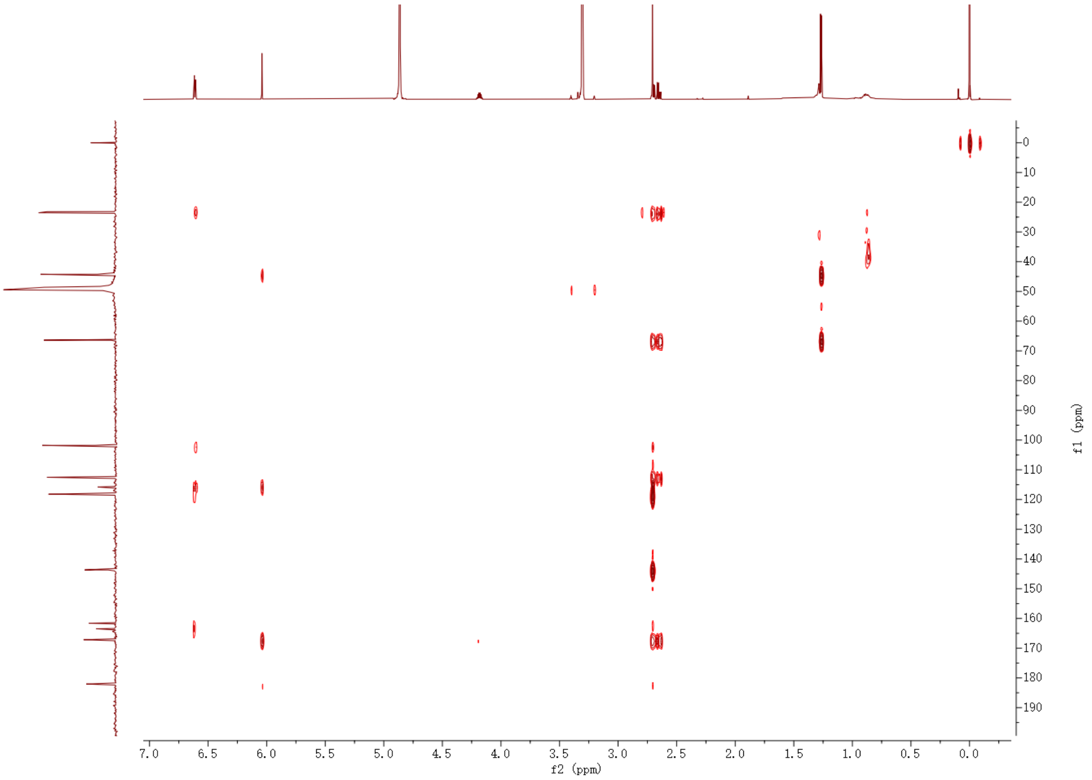

Figure S12. ^{1}H–^{1}H COSY spectrum of compound **1**

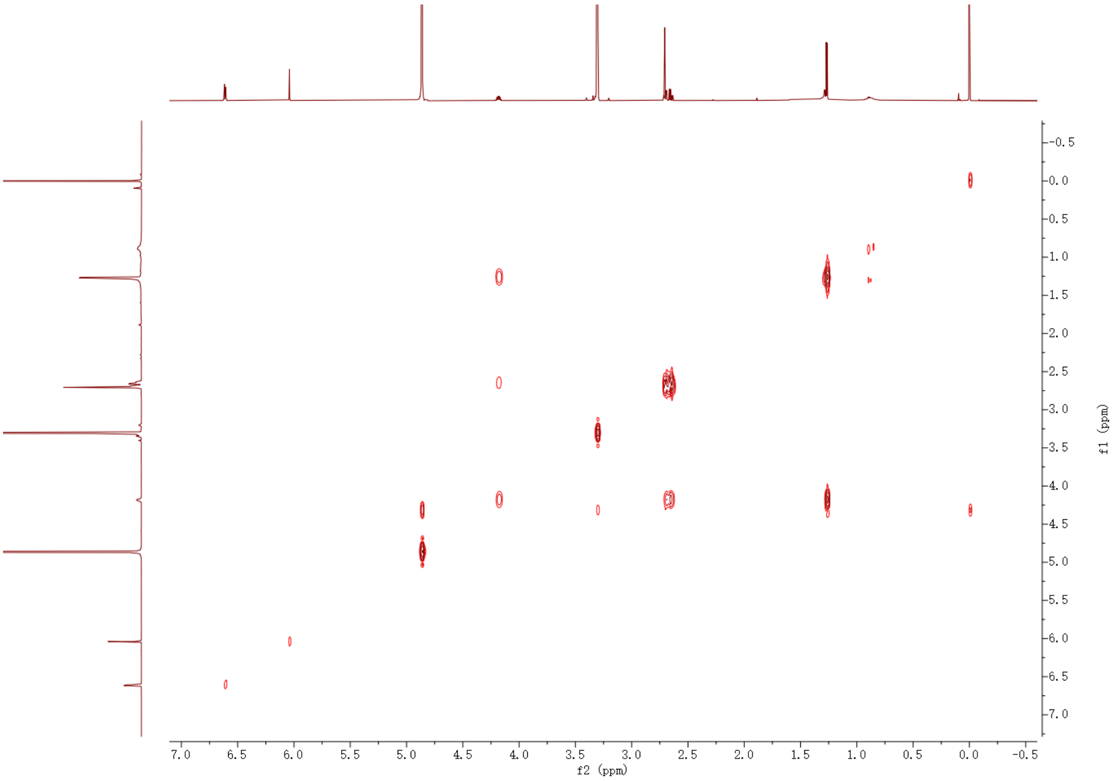

Figure S13. UV spectrum of compound **1**

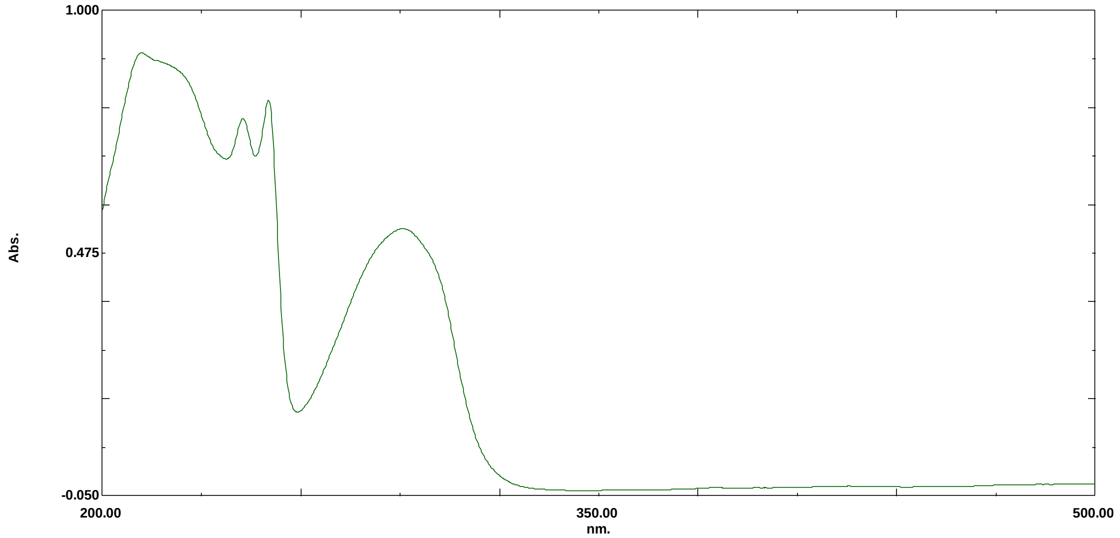

No.	wavelength (nm)	Abs
1	290.80	0.528
2	250.20	0.807
3	242.60	0.766
4	211.80	0.909

Figure S14. IR spectrum of compound **1**

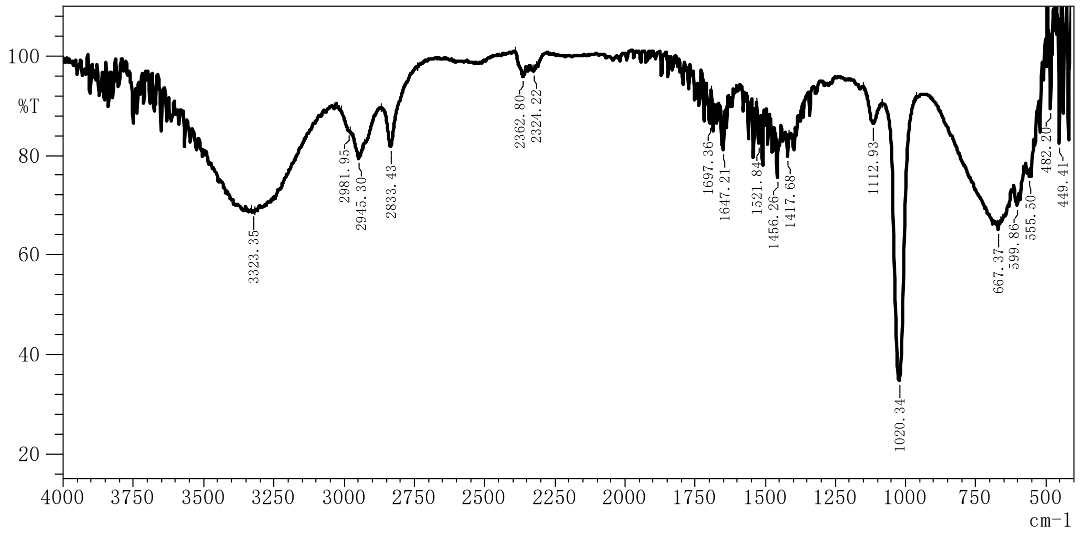

Figure S15. CD spectrum of compound **1a**

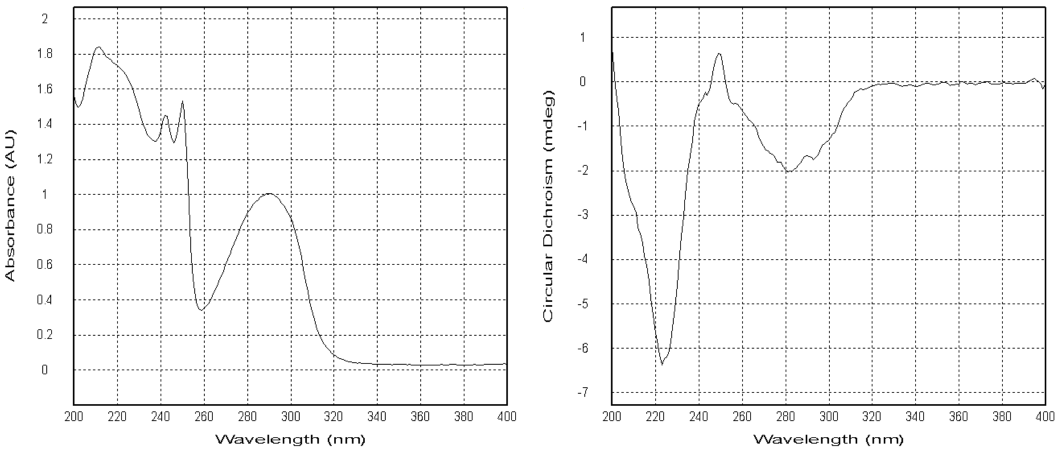

Figure S16. CD spectrum of compound **1b**

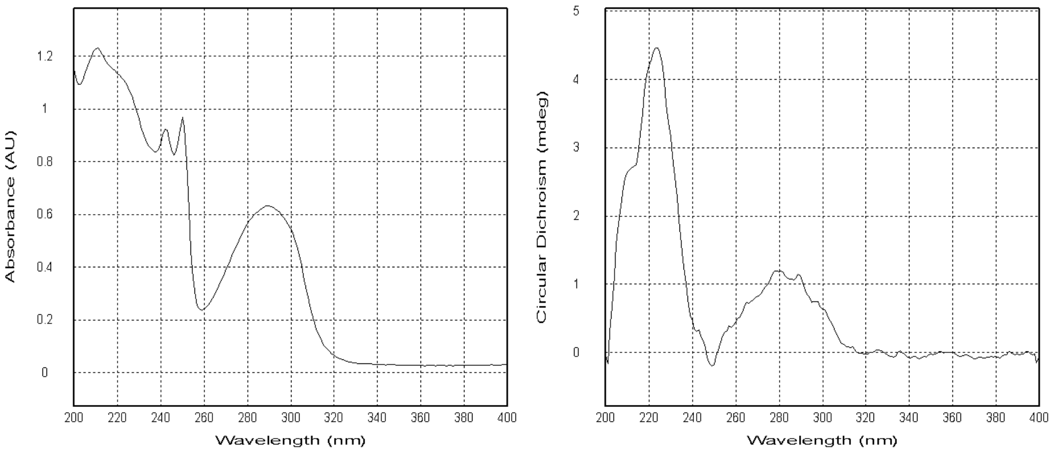

Figure S17. HRESIMS spectrum of compound **2**

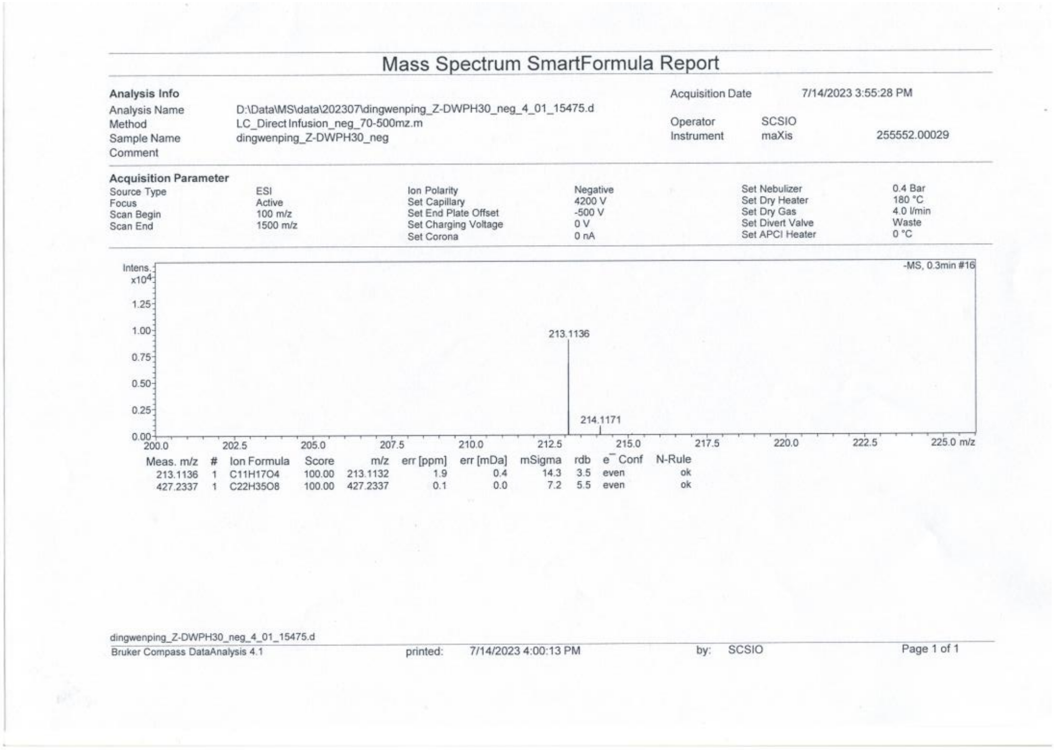

Figure S18. ^{1}H NMR spectrum (CD_3OD, 700 MHz) of compound **2**

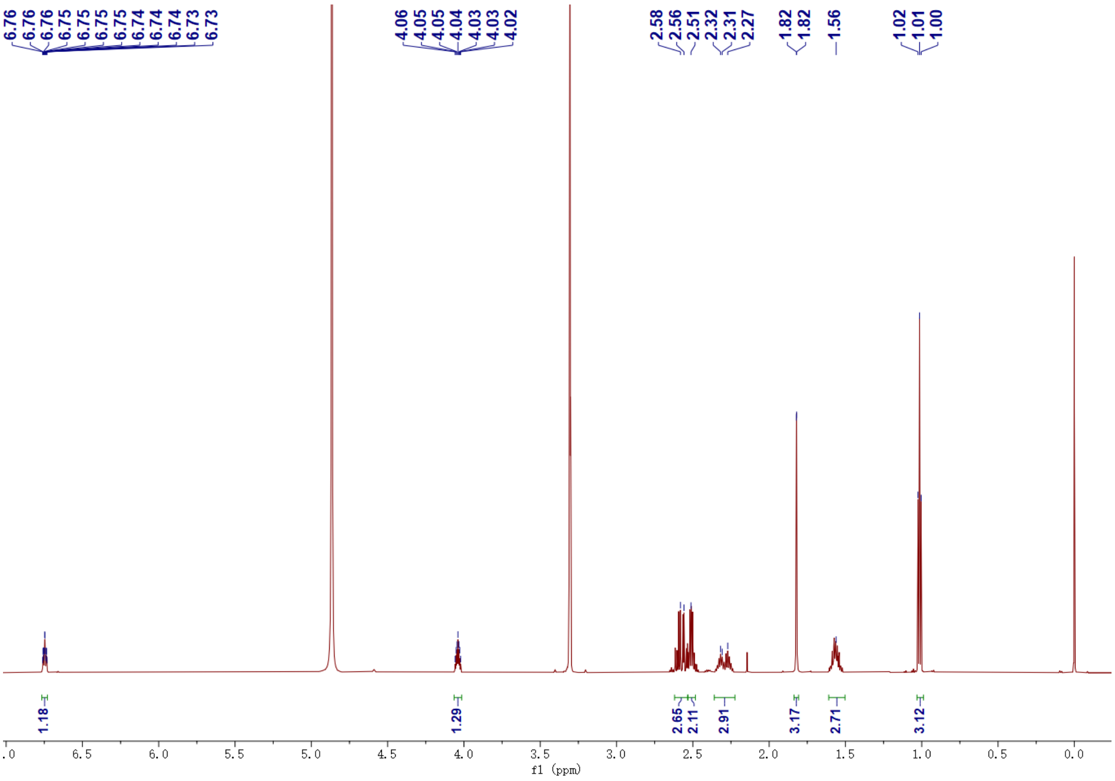

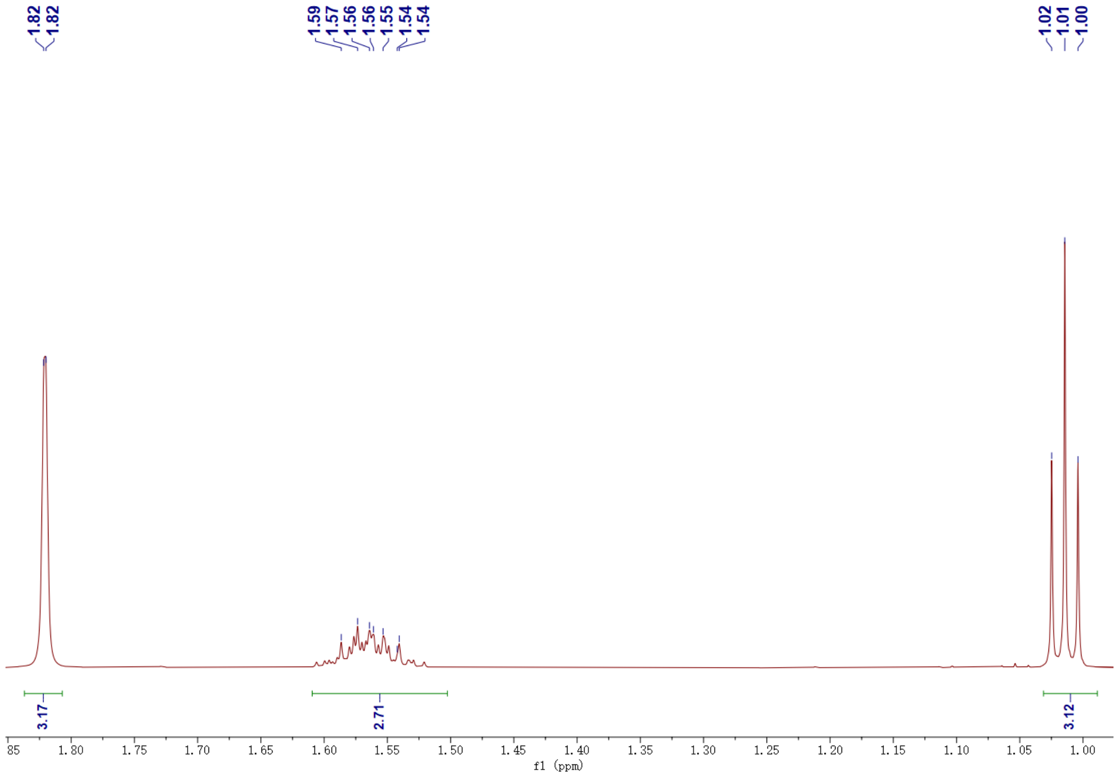
1.82
1.82
1.59
1.57
1.56
1.56
1.55
1.54
1.54
1.02
1.01
1.00
3.17
2.71
3.12
f1 (ppm)

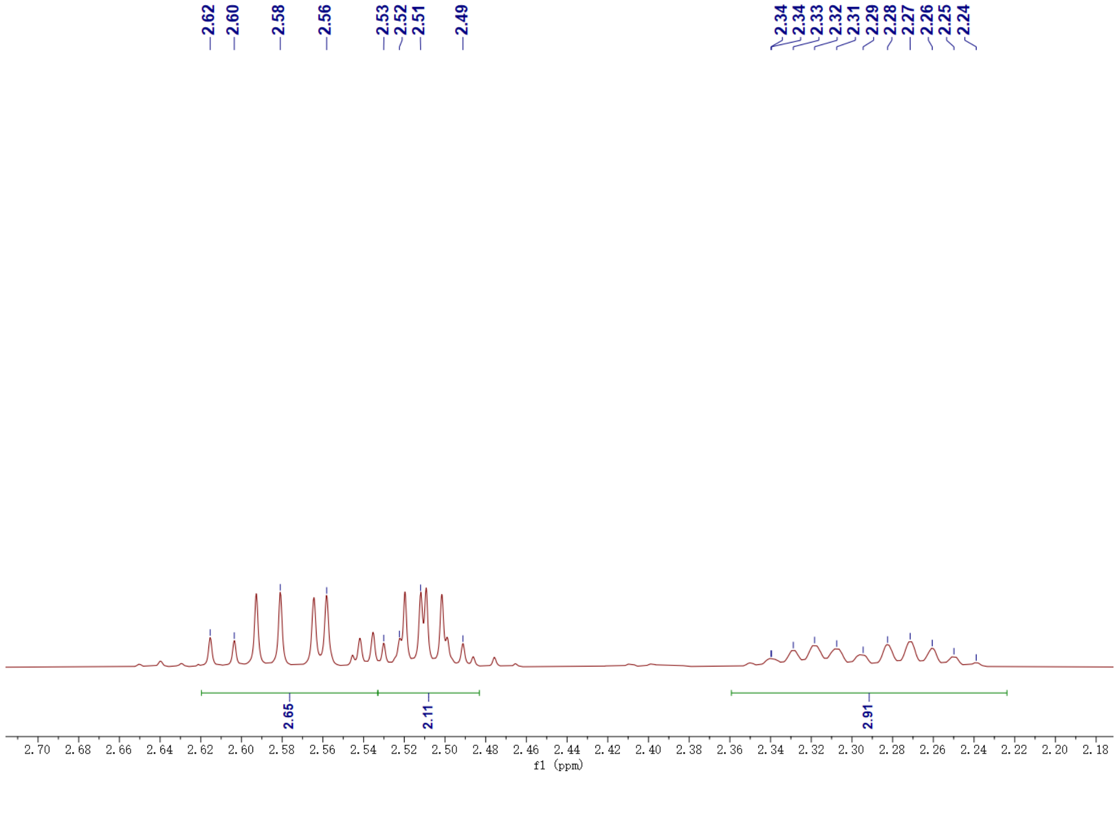
2.62
2.60
2.58
2.56
2.53
2.52
2.51
2.49
2.34
2.34
2.33
2.32
2.31
2.29
2.28
2.27
2.26
2.25
2.24
2.65
2.11
2.91
f1 (ppm)

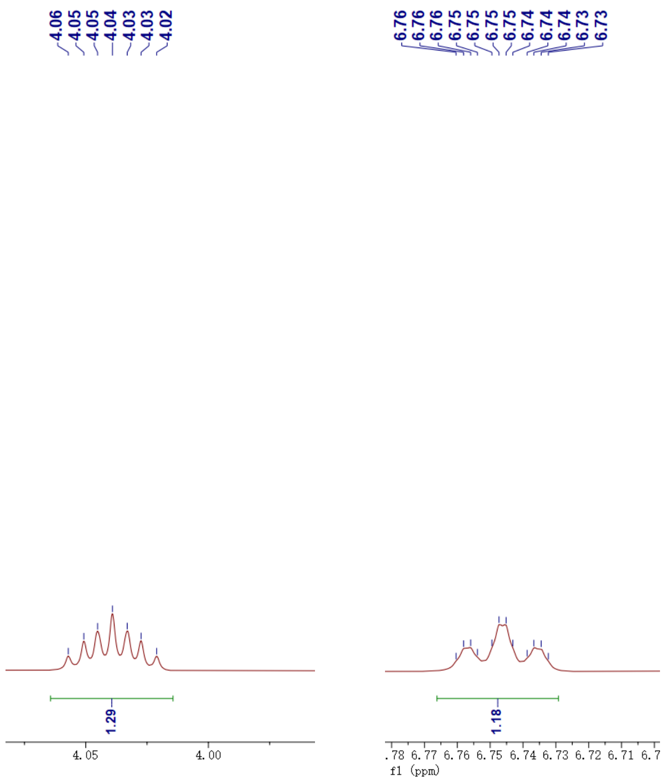

Figure S19. ^{13}C NMR and DEPT spectra (CD_3OD, 176 MHz) of compound **2**

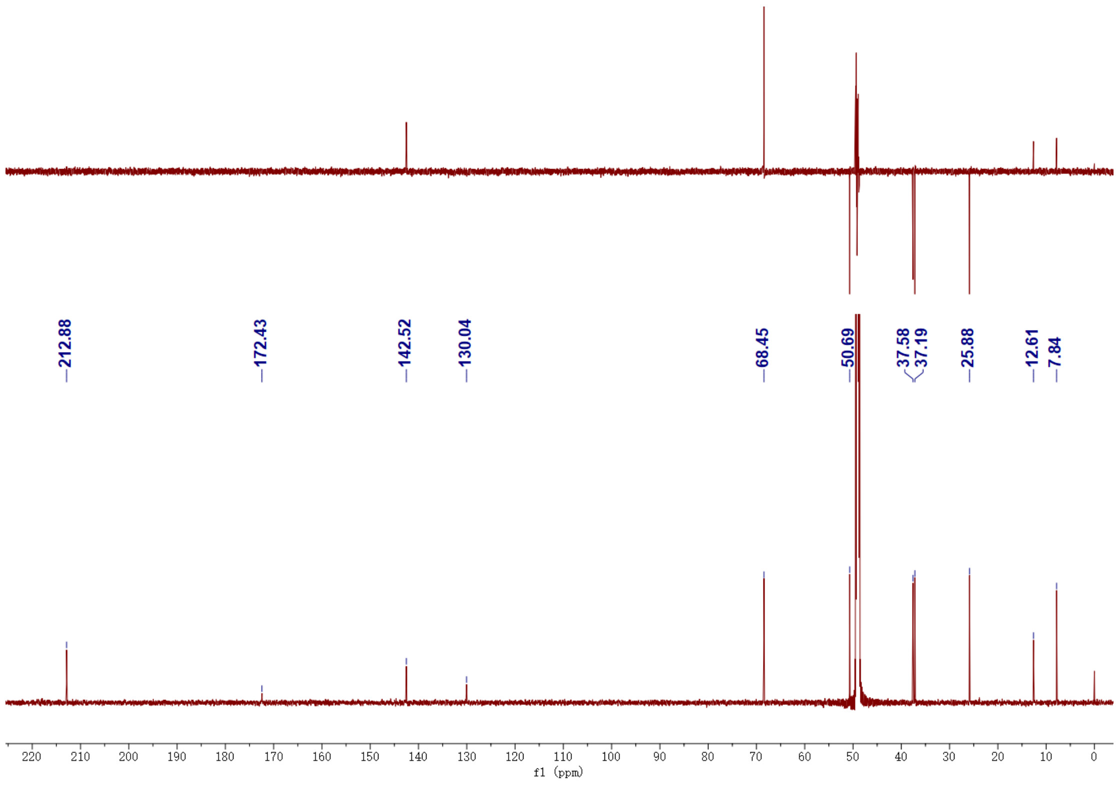

Figure S20. HSQC spectrum of compound **2**

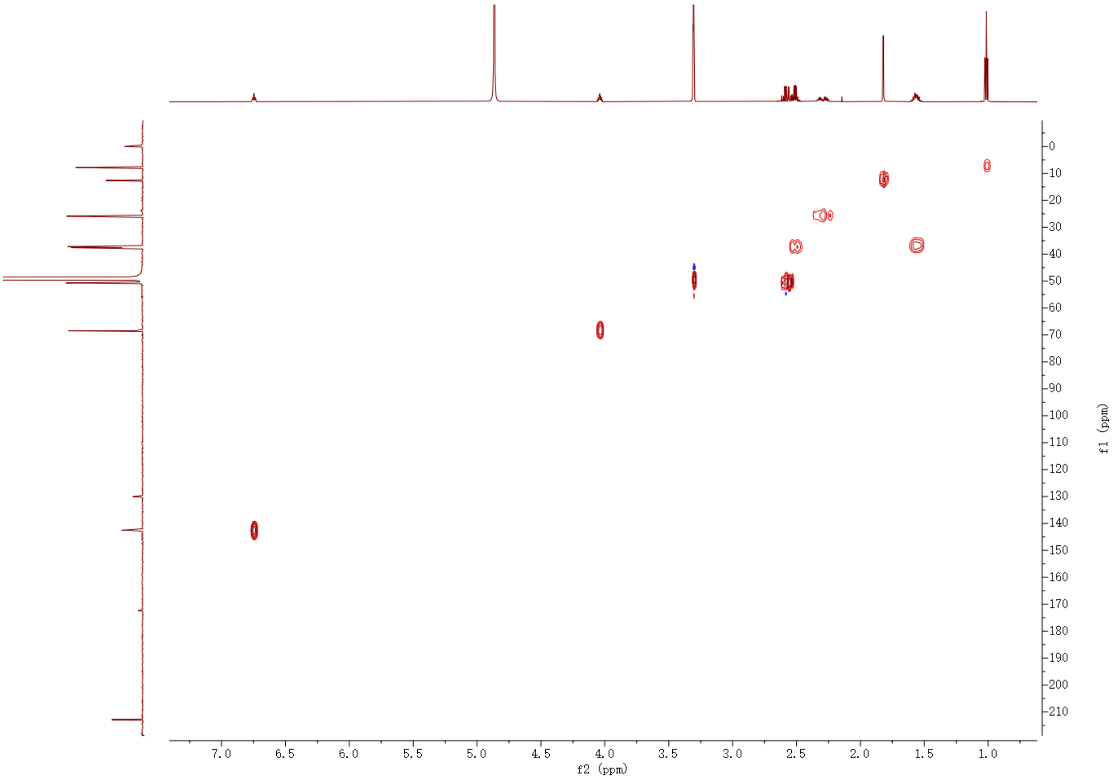

Figure S21. HMBC spectrum of compound **2**

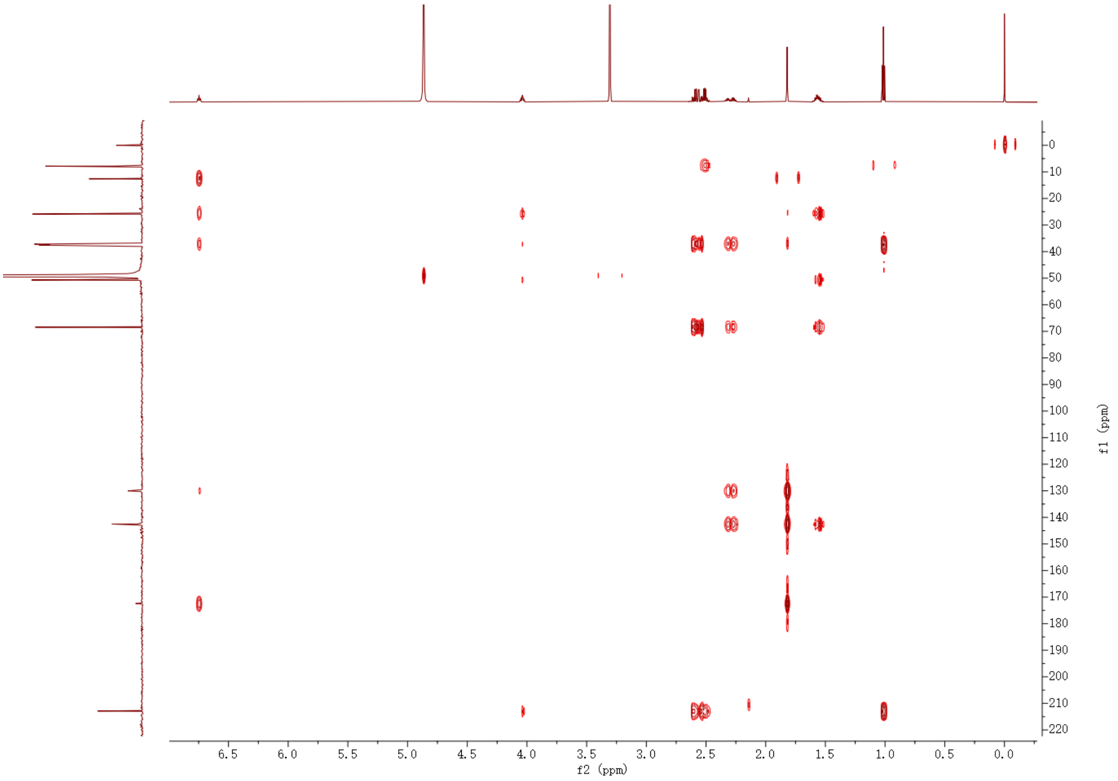

Figure S22. ^{1}H–^{1}H COSY spectrum of compound **2**

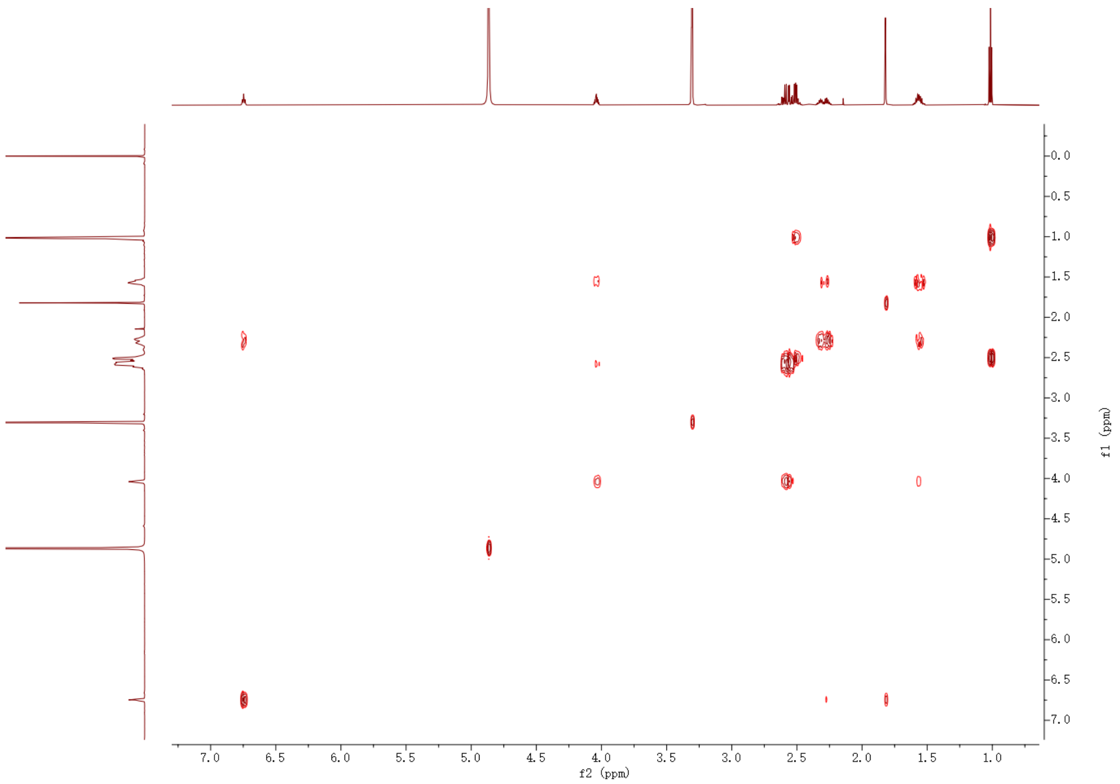

Figure S23. NOESY spectrum of compound **2**

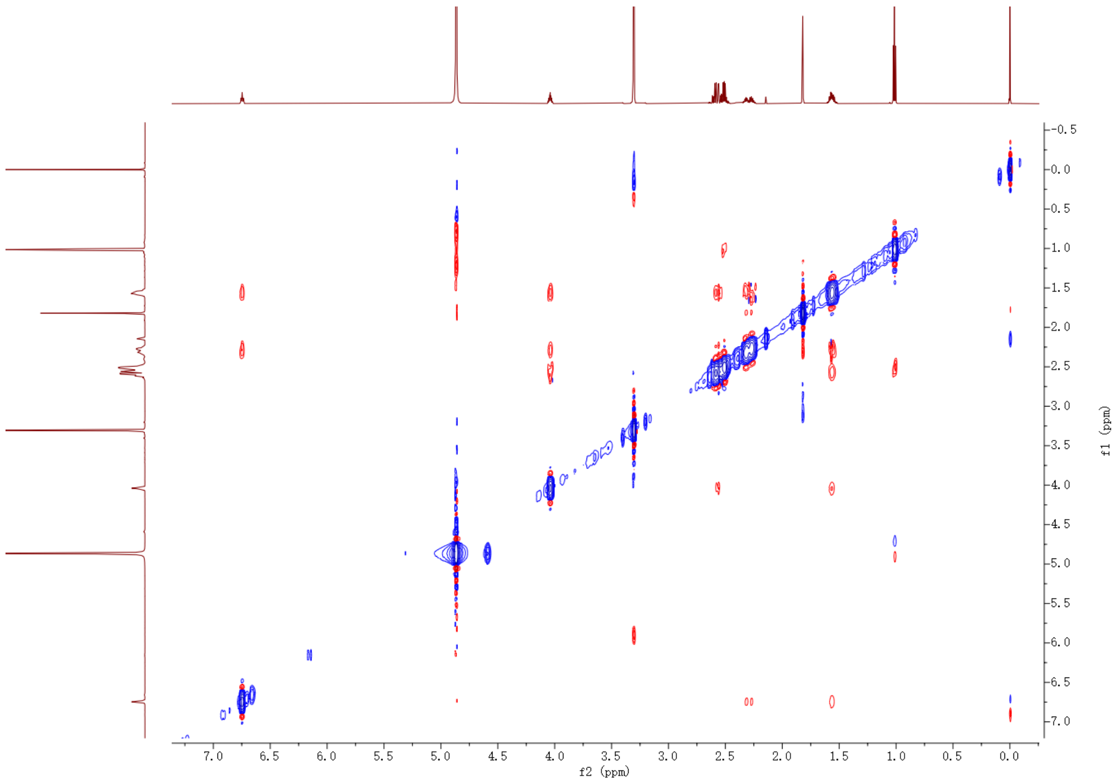

Figure S24. UV spectrum of compound **2**

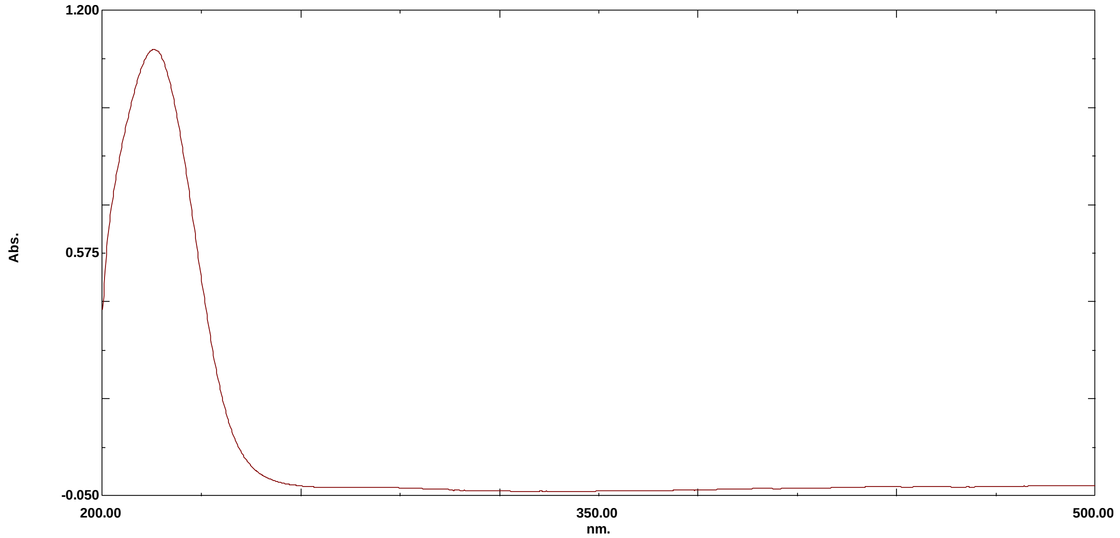

No.	wavelength (nm)	Abs
1	215.40	1.099

Figure S25. IR spectrum of compound **2**

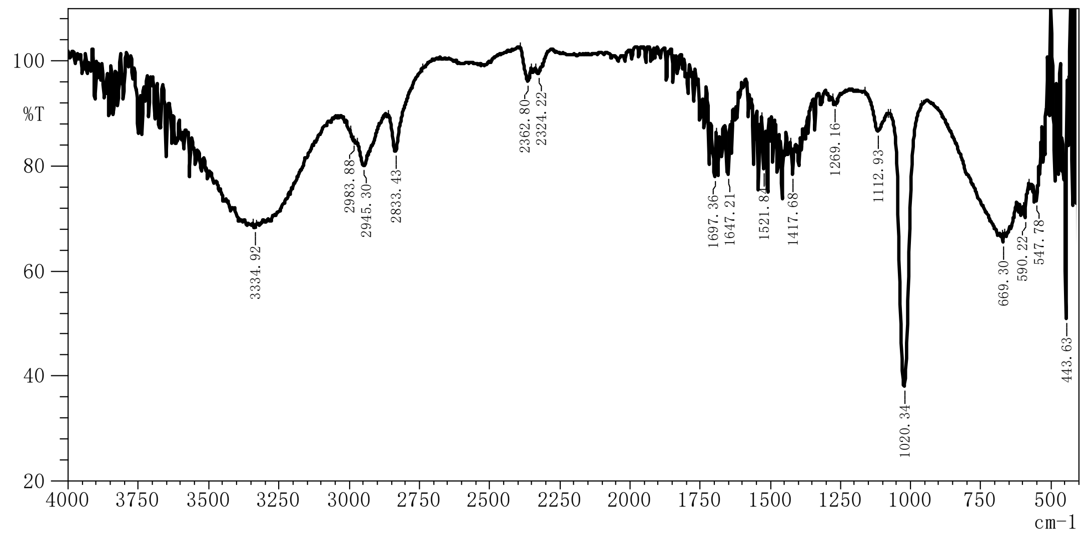

Figure S26. CD spectrum of compound **2a**

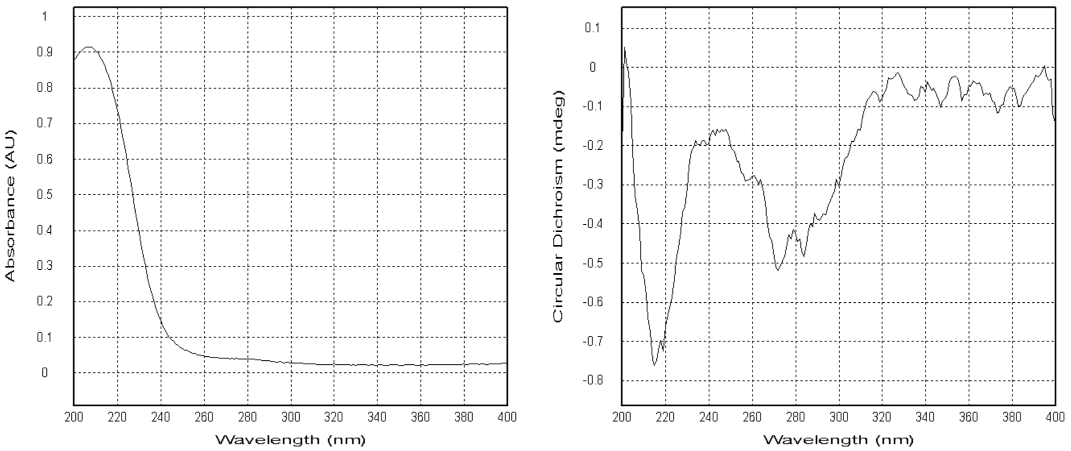

Figure S27. HRESIMS spectrum of compound **3**

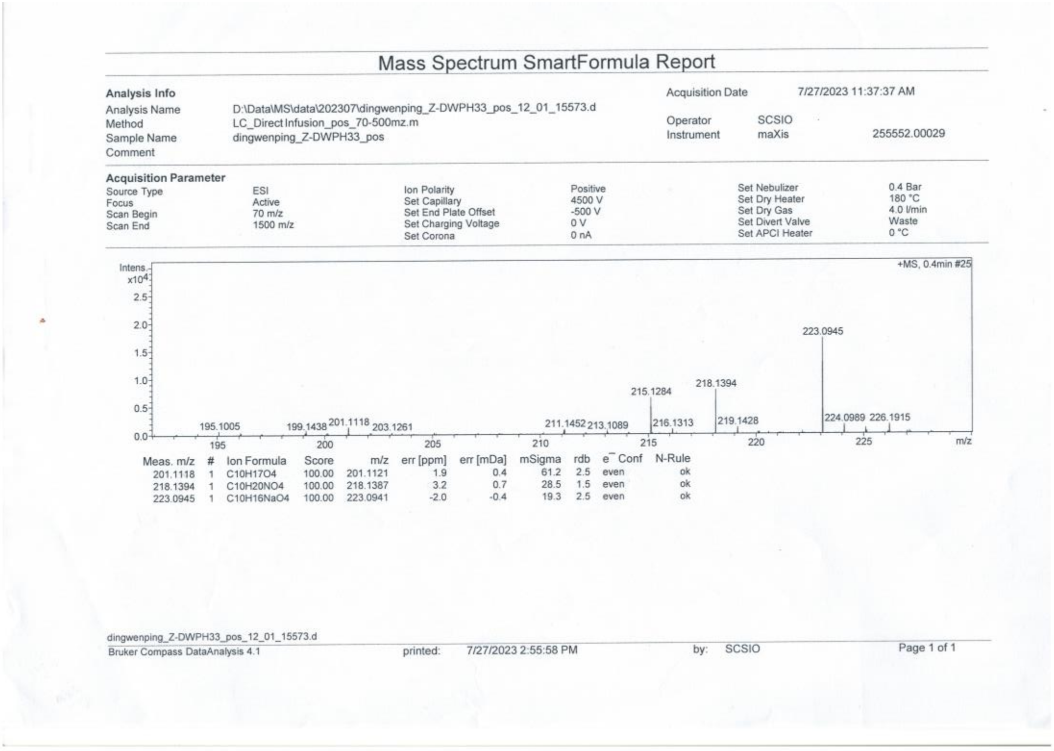

Mass Spectrum SmartFormula Report

**Analysis Info**		Acquisition Date	7/27/2023 11:37:37 AM
Analysis Name	D:\Data\MS\data\202307\dingwenping_Z-DWPH33_pos_12_01_15573.d		
Method	LC_Direct Infusion_pos_70-500mz.m	Operator	SCSIO
Sample Name	dingwenping_Z-DWPH33_pos	Instrument	maXis 255552.00029
Comment			

Acquisition Parameter

Source Type	ESI	Ion Polarity	Positive	Set Nebulizer	0.4 Bar
Focus	Active	Set Capillary	4500 V	Set Dry Heater	180 °C
Scan Begin	70 m/z	Set End Plate Offset	-500 V	Set Dry Gas	4.0 l/min
Scan End	1500 m/z	Set Charging Voltage	0 V	Set Divert Valve	Waste
		Set Corona	0 nA	Set APCI Heater	0 °C

Meas. m/z	#	Ion Formula	Score	m/z	err [ppm]	err [mDa]	mSigma	rdb	e⁻ Conf	N-Rule
201.1118	1	C10H17O4	100.00	201.1121	1.9	0.4	61.2	2.5	even	ok
218.1394	1	C10H20NO4	100.00	218.1387	3.2	0.7	28.5	1.5	even	ok
223.0945	1	C10H16NaO4	100.00	223.0941	-2.0	-0.4	19.3	2.5	even	ok

dingwenping_Z-DWPH33_pos_12_01_15573.d
Bruker Compass DataAnalysis 4.1 printed: 7/27/2023 2:55:58 PM by: SCSIO Page 1 of 1

Figure S28. ^{1}H NMR spectrum (CD_3OD, 700 MHz) of compound **3**

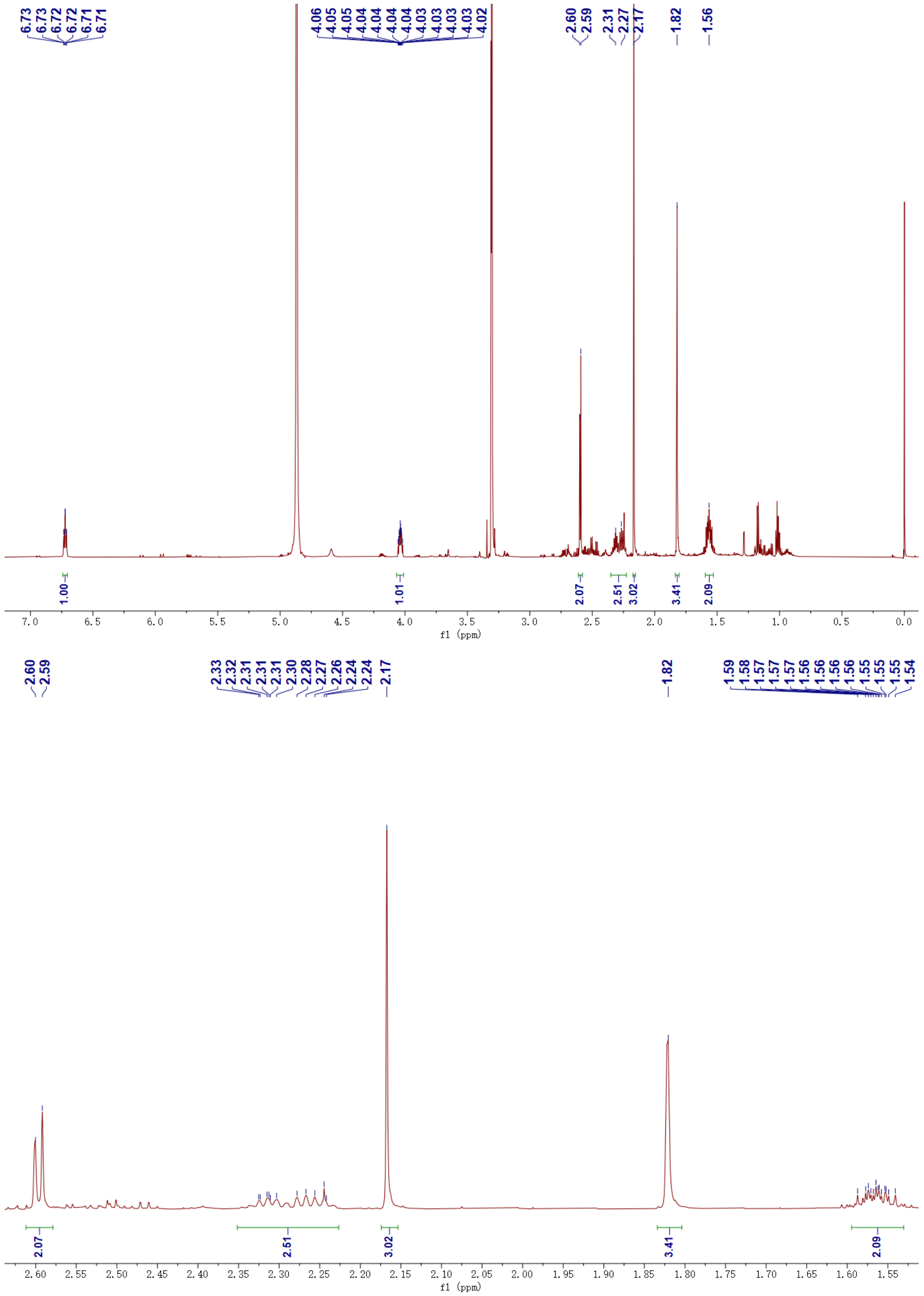

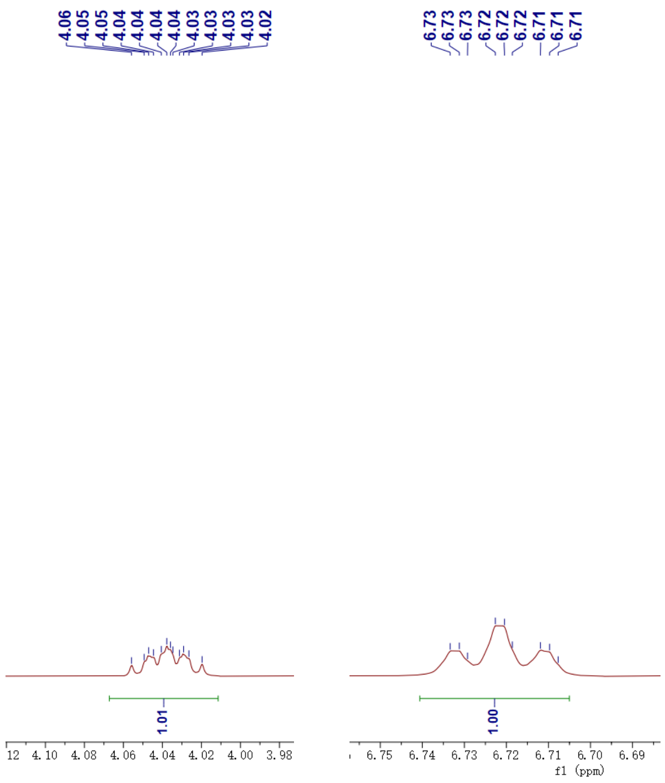

Figure S29. ^{13}C NMR and DEPT spectra (CD3OD, 176 MHz) of compound **3**

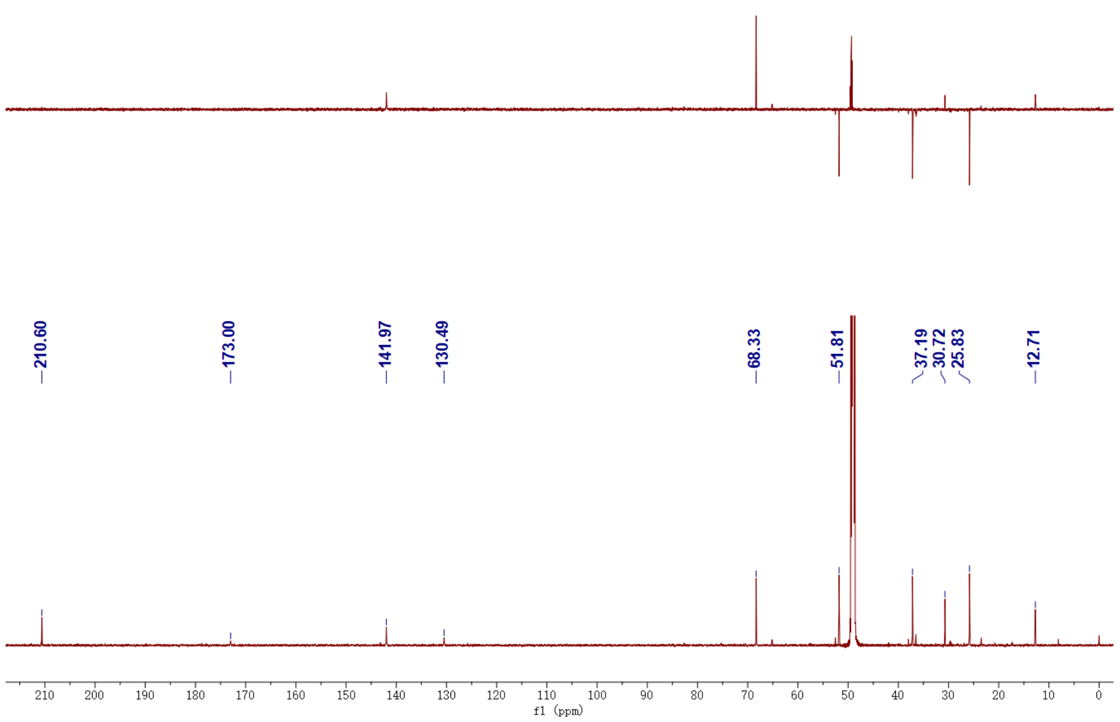

Figure S30. HSQC spectrum of compound **3**

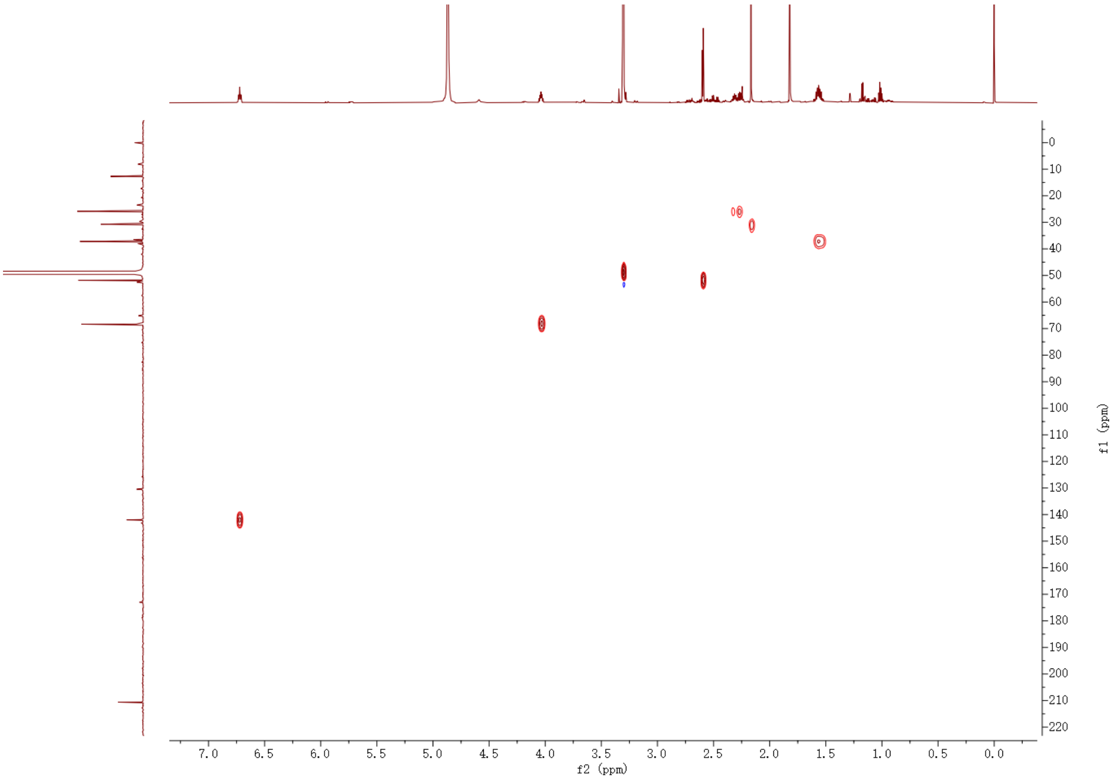

Figure S31. HMBC spectrum of compound **3**

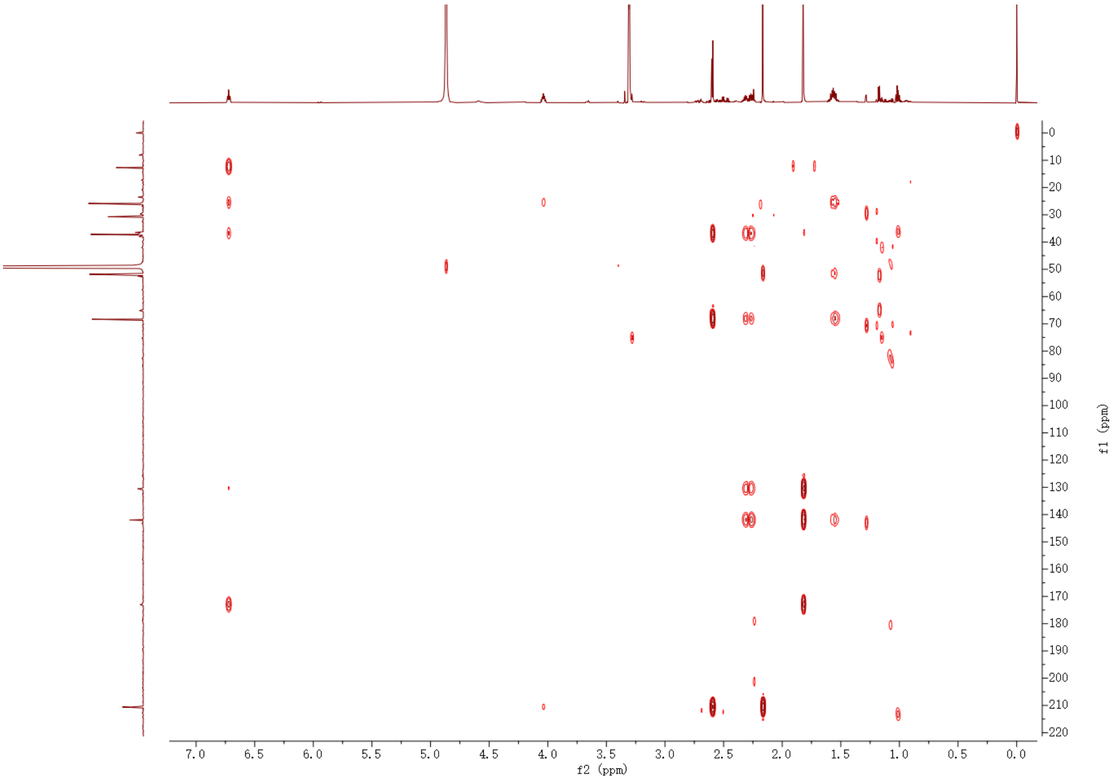

Figure S32. ^1H–^1H COSY spectrum of compound **3**

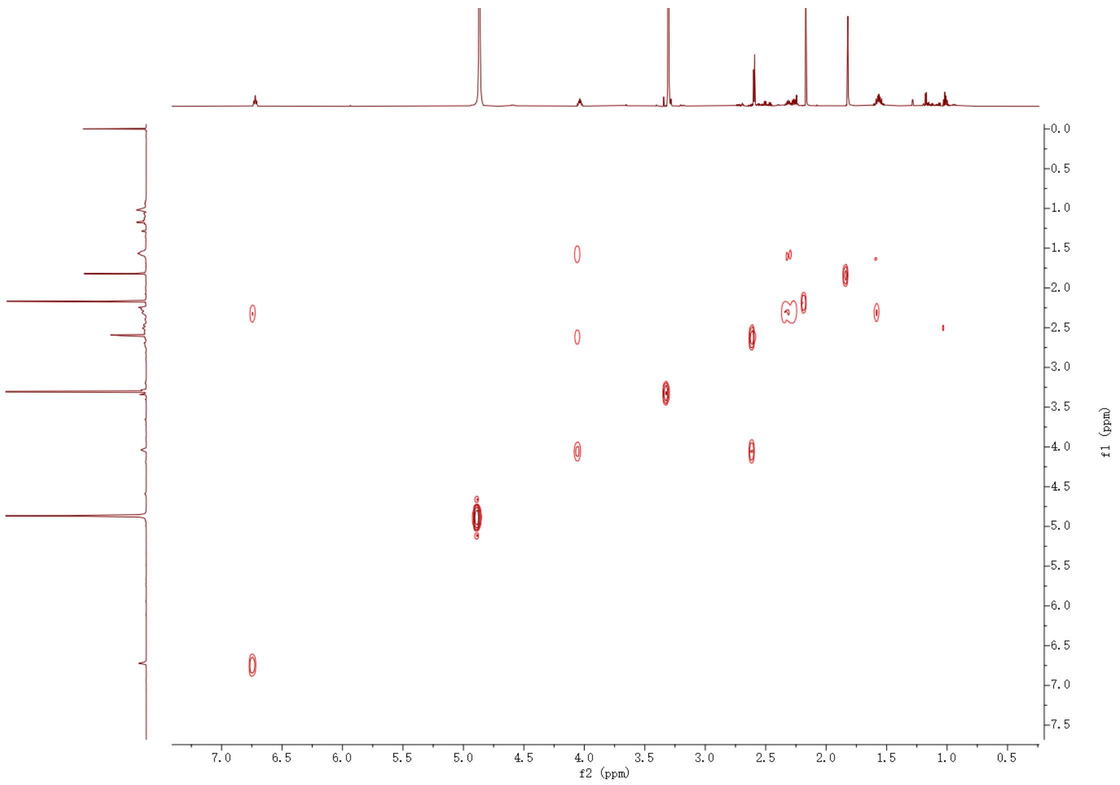

Figure S33. NOESY spectrum of compound **3**

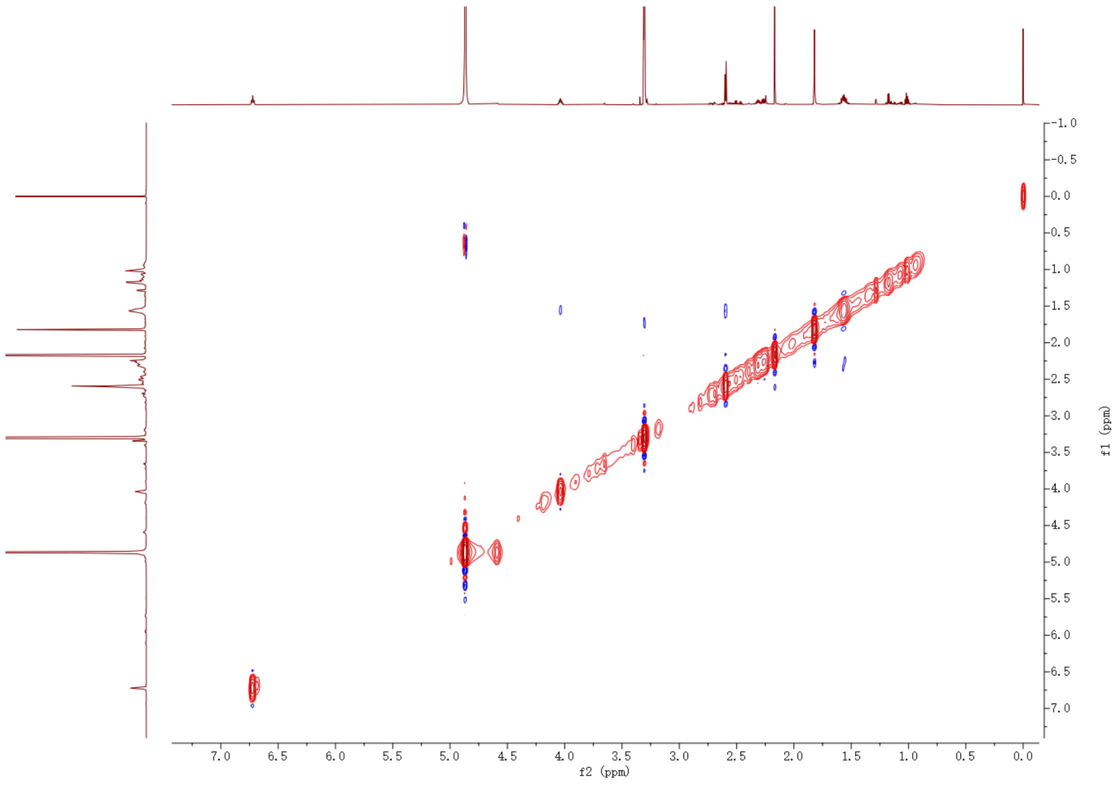

Figure S34. UV spectrum of compound **3**

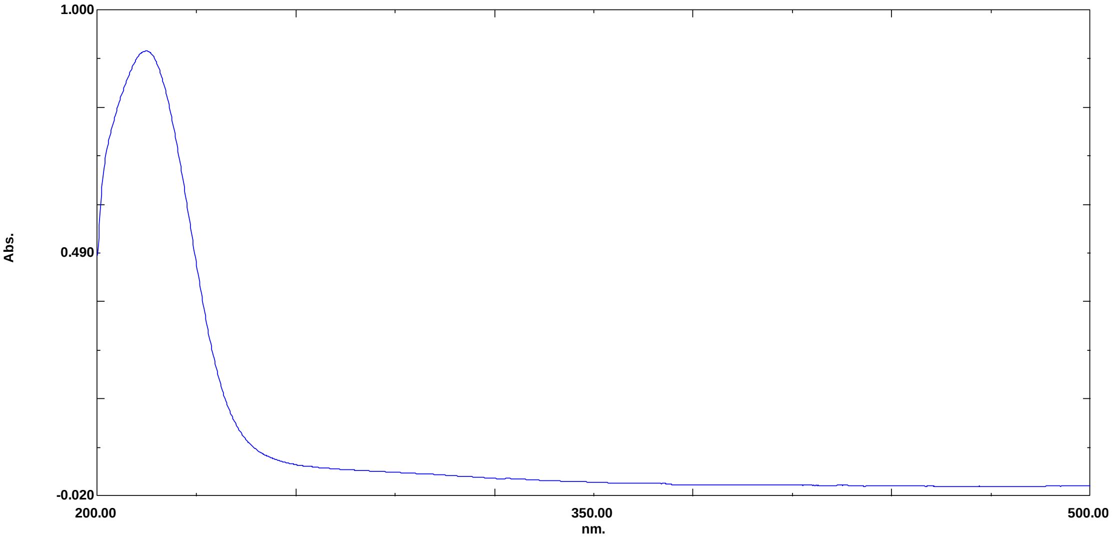

No.	wavelength (nm)	Abs
1	214.80	0.914

Figure S35. IR spectrum of compound **3**

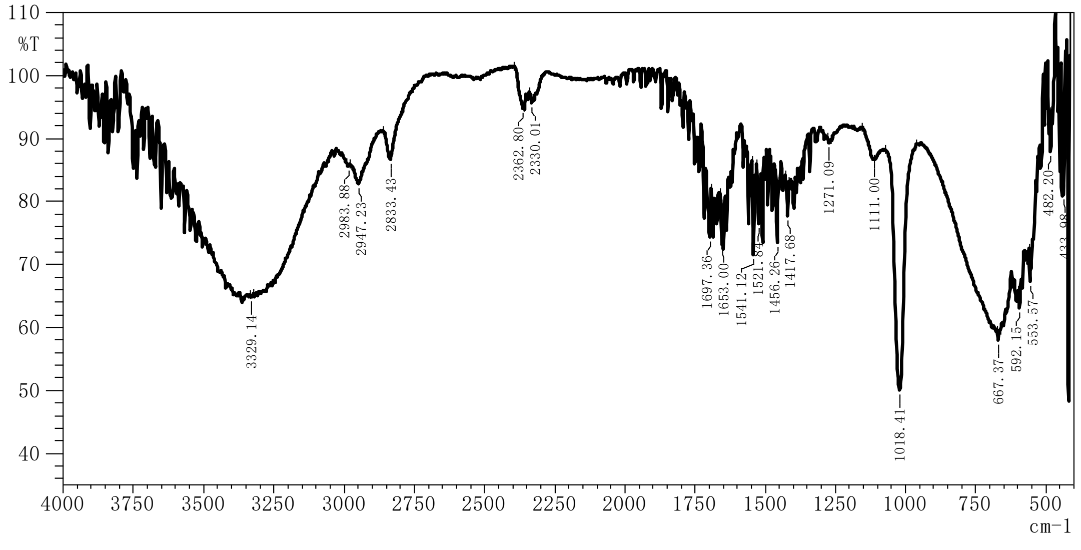

Figure S36. CD spectrum of compound **3a**

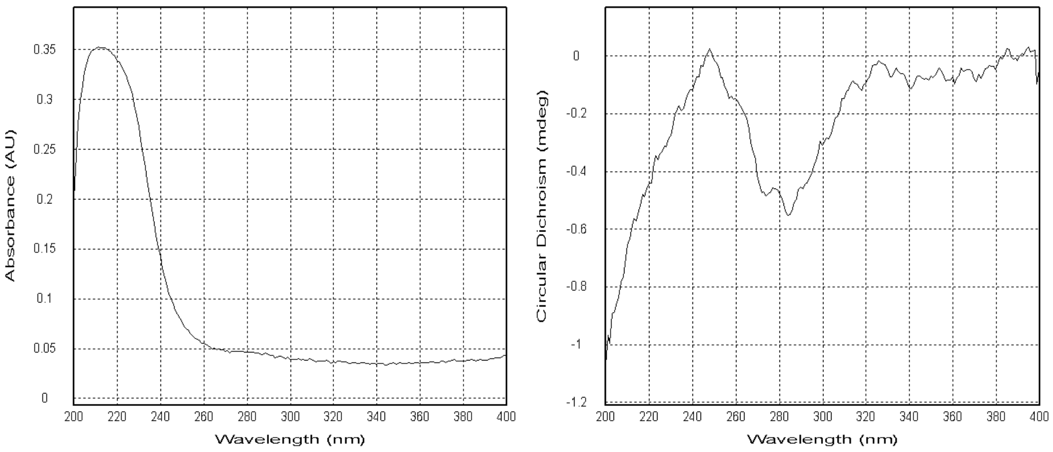

Figure S37. CD spectrum of compound **3b**

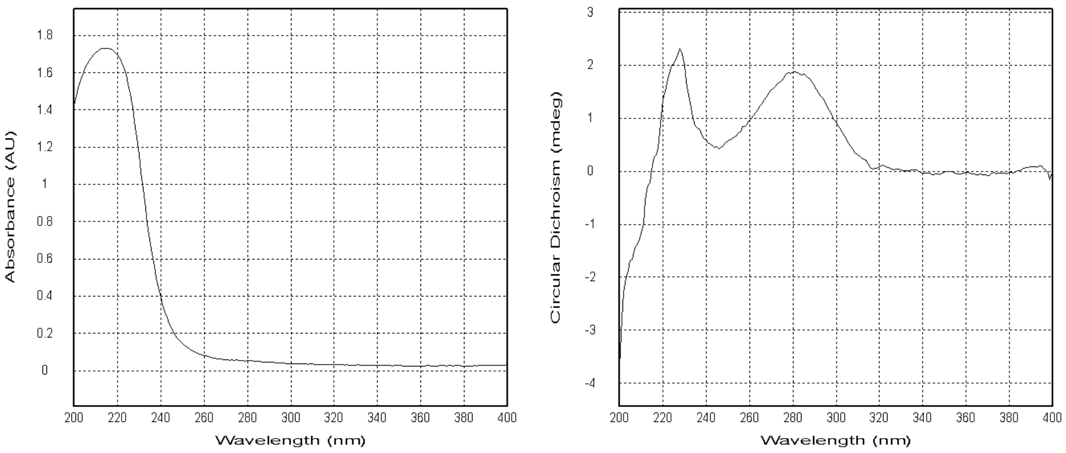

Figure S38. HRESIMS spectrum of compound **4**

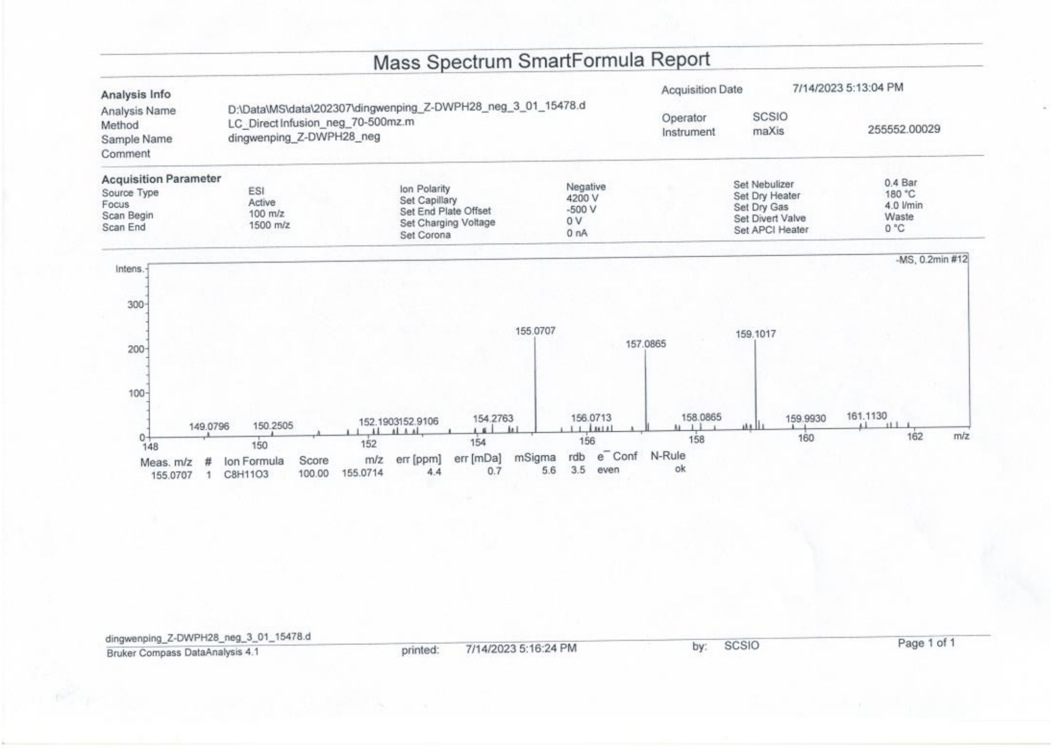

Figure S39. ^{1}H NMR spectrum (CD_3OD, 500 MHz) of compound **4**

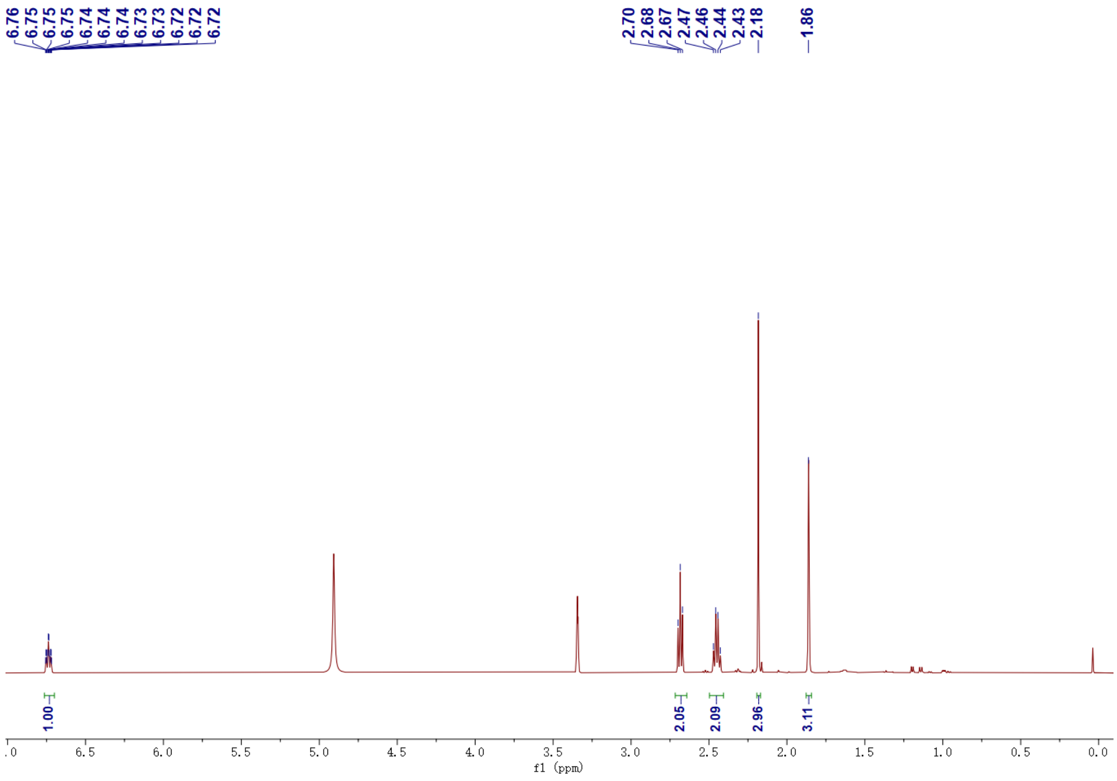

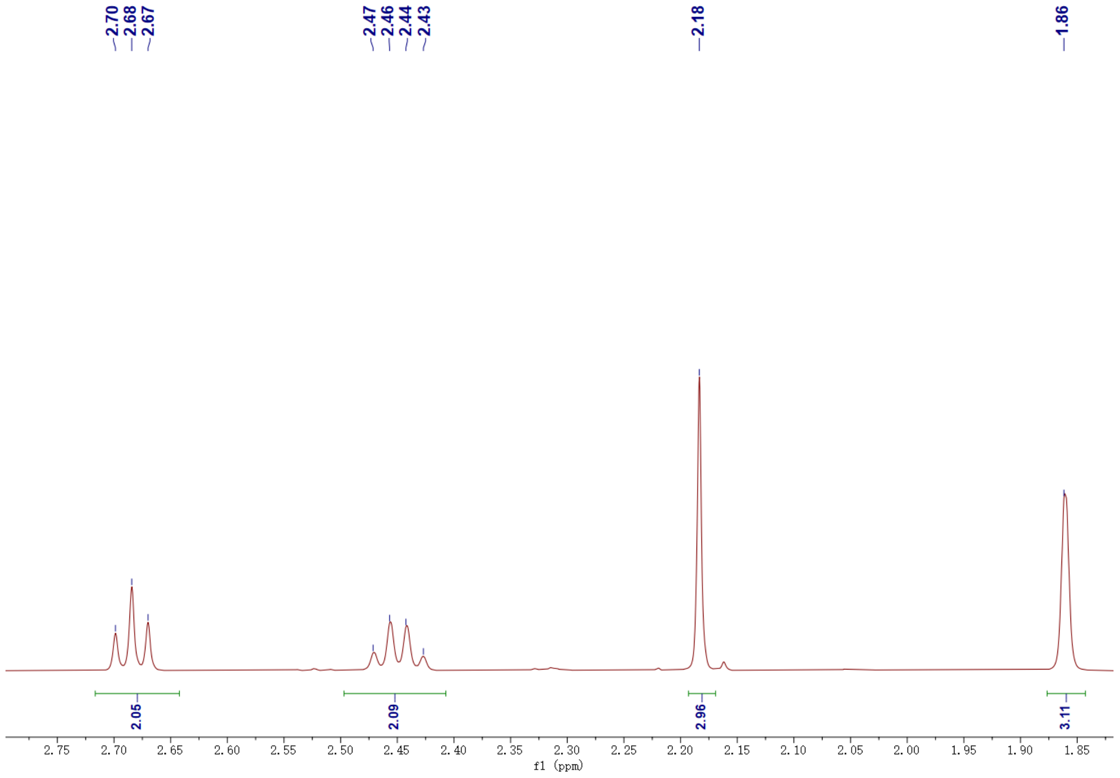
2.70
2.68
2.67
2.47
2.46
2.44
2.43
2.18
1.86
2.05
2.09
2.96
3.11
f1 (ppm)

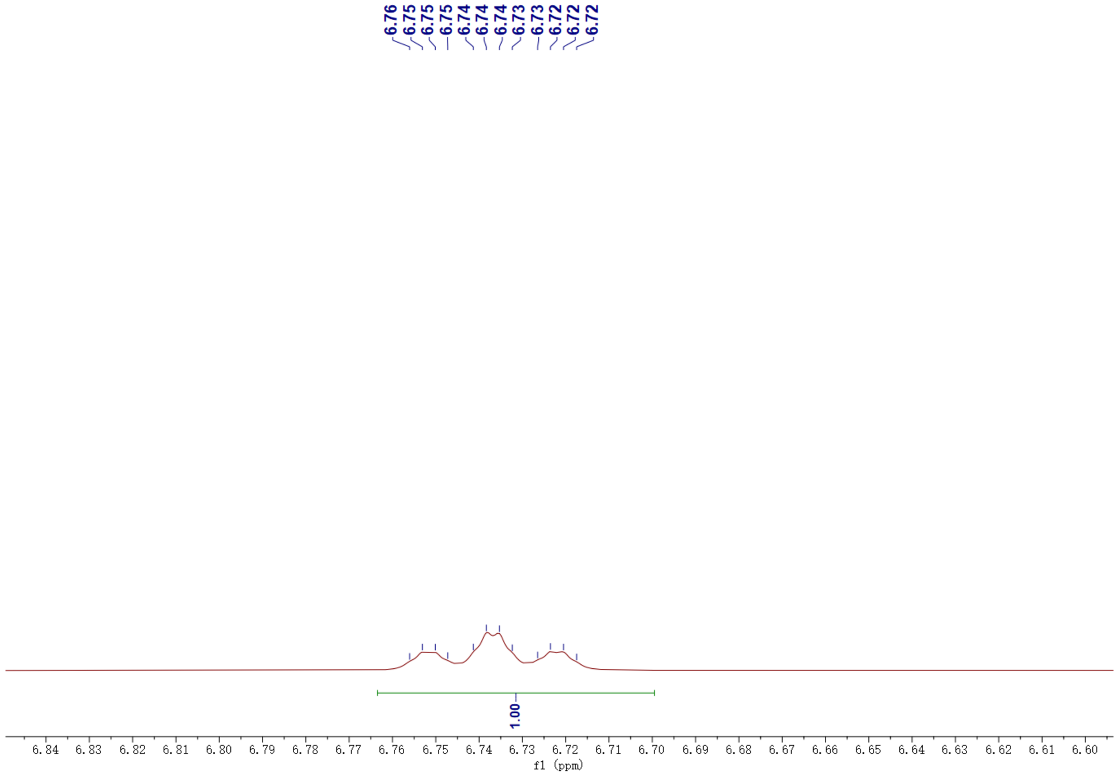
6.76
6.75
6.75
6.75
6.74
6.74
6.74
6.73
6.73
6.72
6.72
6.72
1.00
f1 (ppm)

Figure S40. ^{13}C NMR and DEPT spectra (CD3OD, 126 MHz) of compound **4**

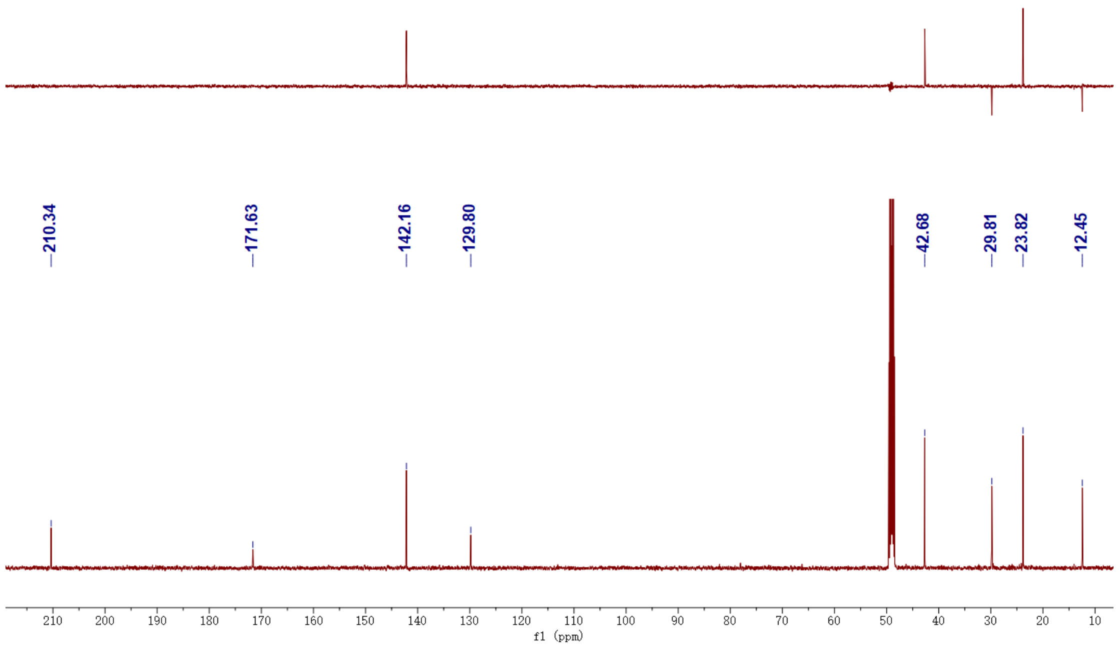

Figure S41. HSQC spectrum of compound **4**

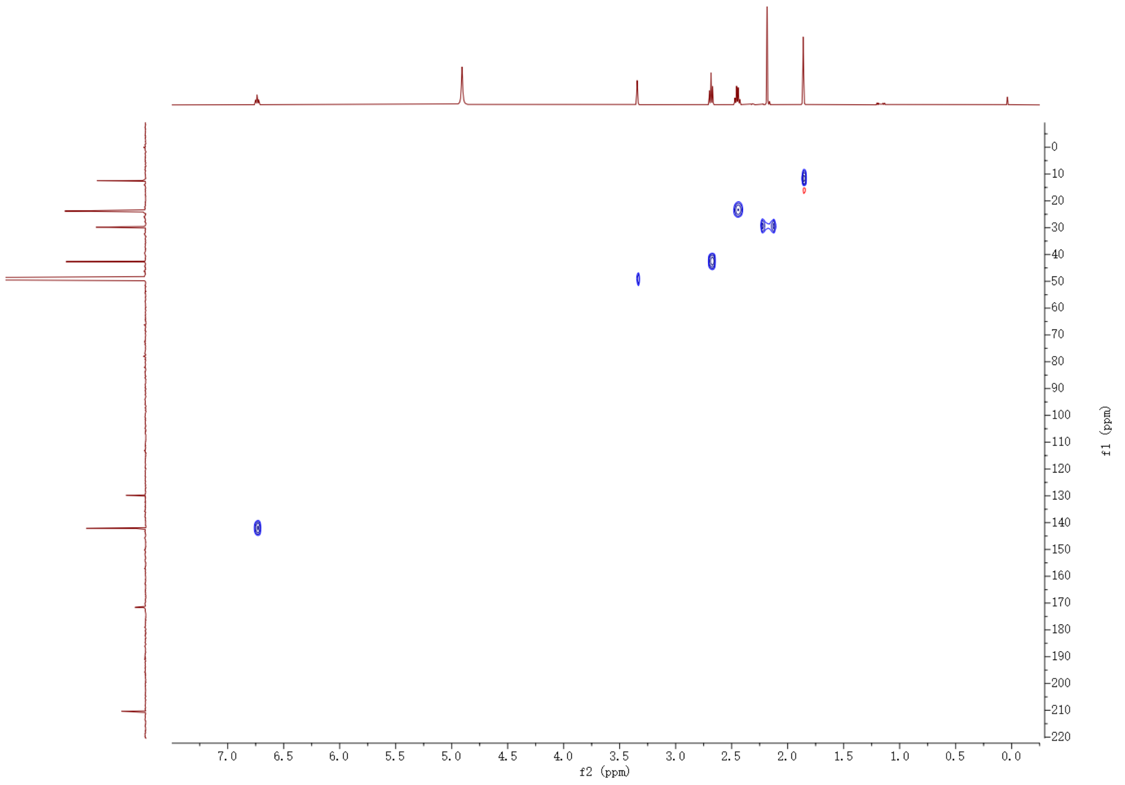

Figure S42. HMBC spectrum of compound **4**

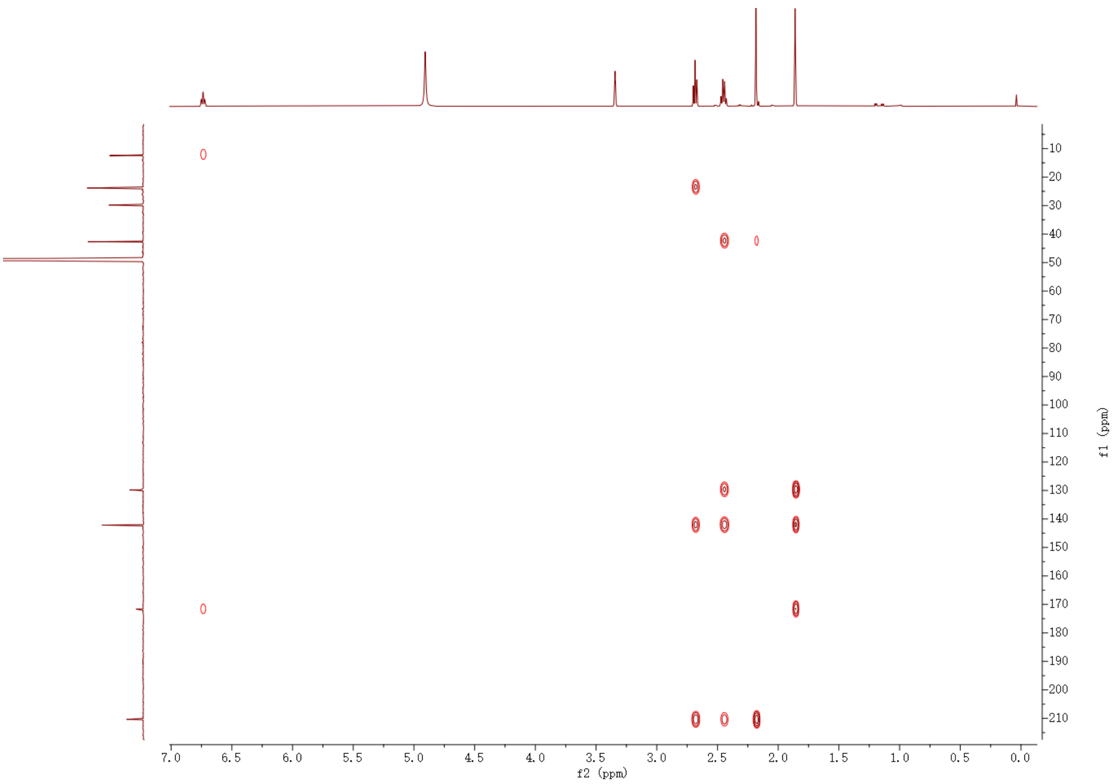

Figure S43 ^{1}H–^{1}H COSY spectrum of compound **4**

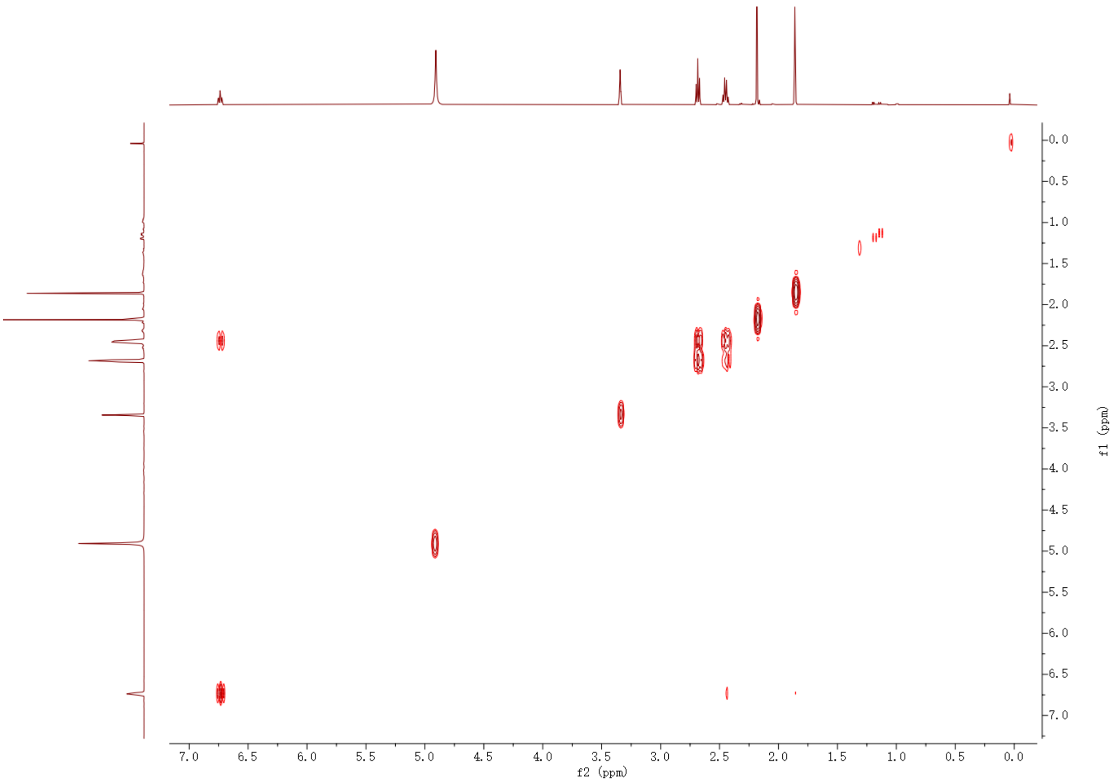

Figure S44. NOESY spectrum of compound **4**

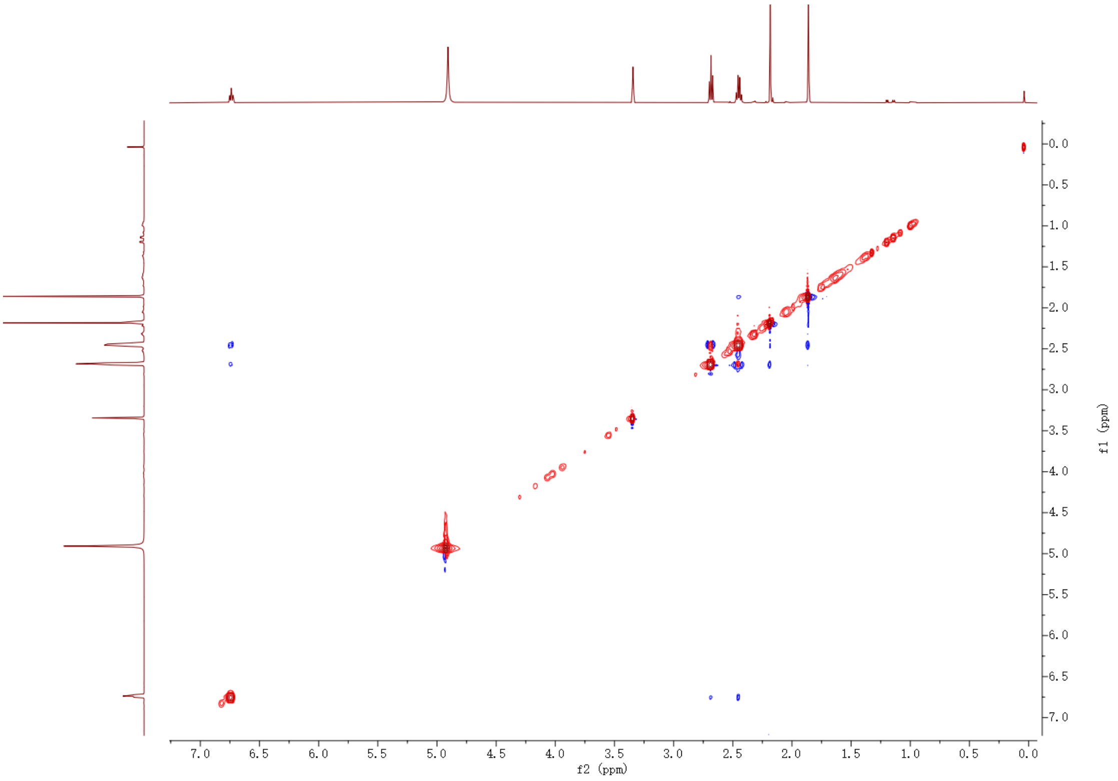

Figure S45. UV spectrum of compound **4**

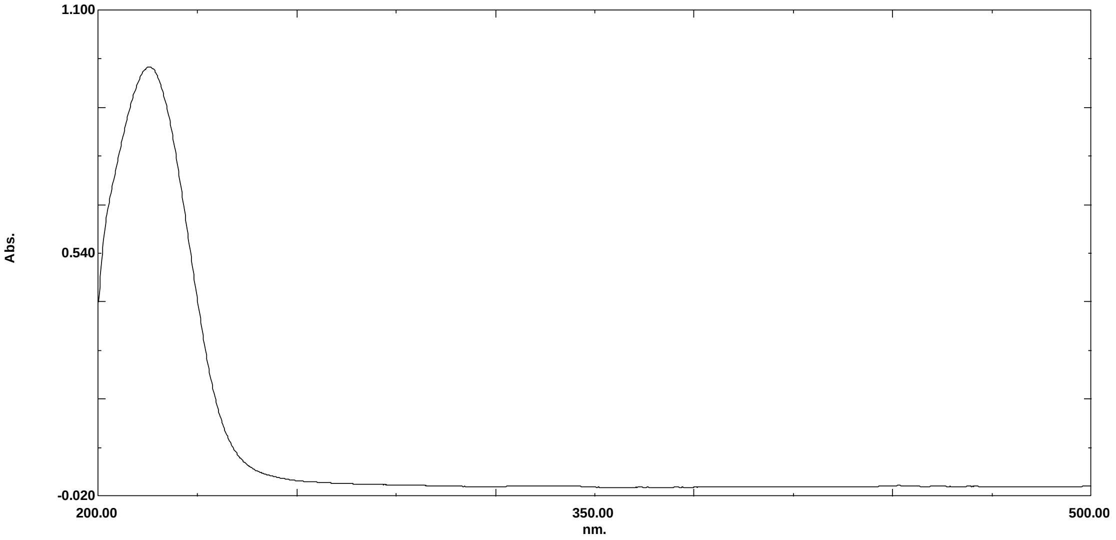

No.	wavelength (nm)	Abs
1	215.20	0.970

Figure S46. IR spectrum of compound **4**

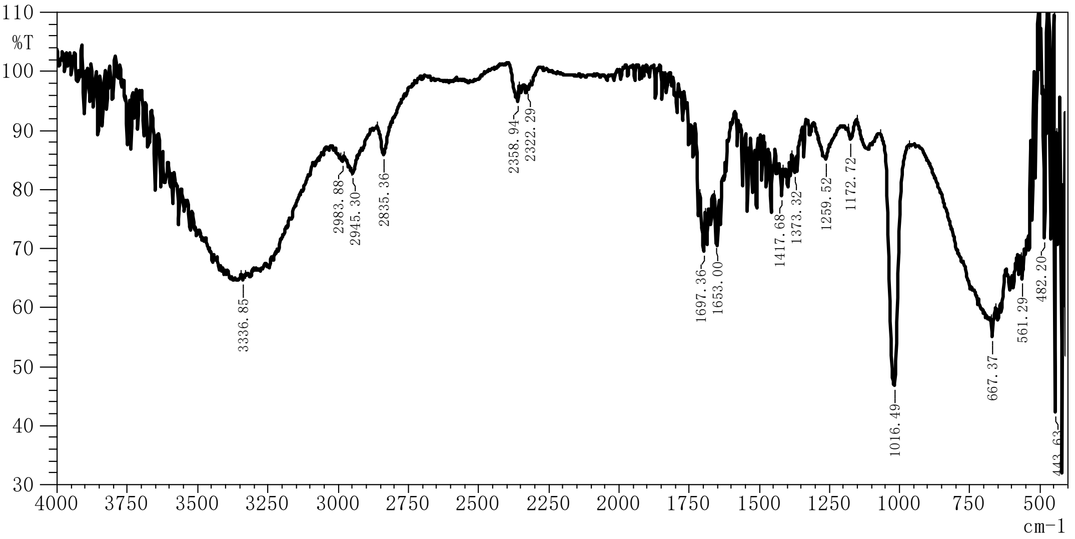

Figure S47. ESIMS spectrum of compound **5**

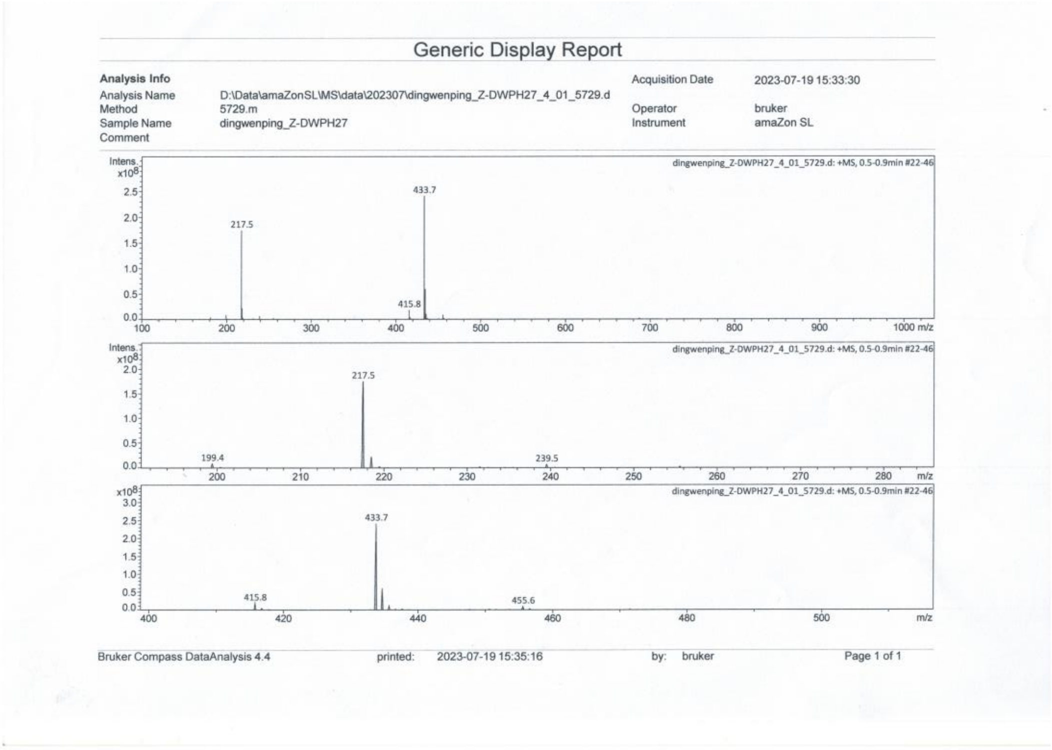

Figure S48. ^{1}H NMR spectrum ($CDCl_3$, 500 MHz) of compound **5**

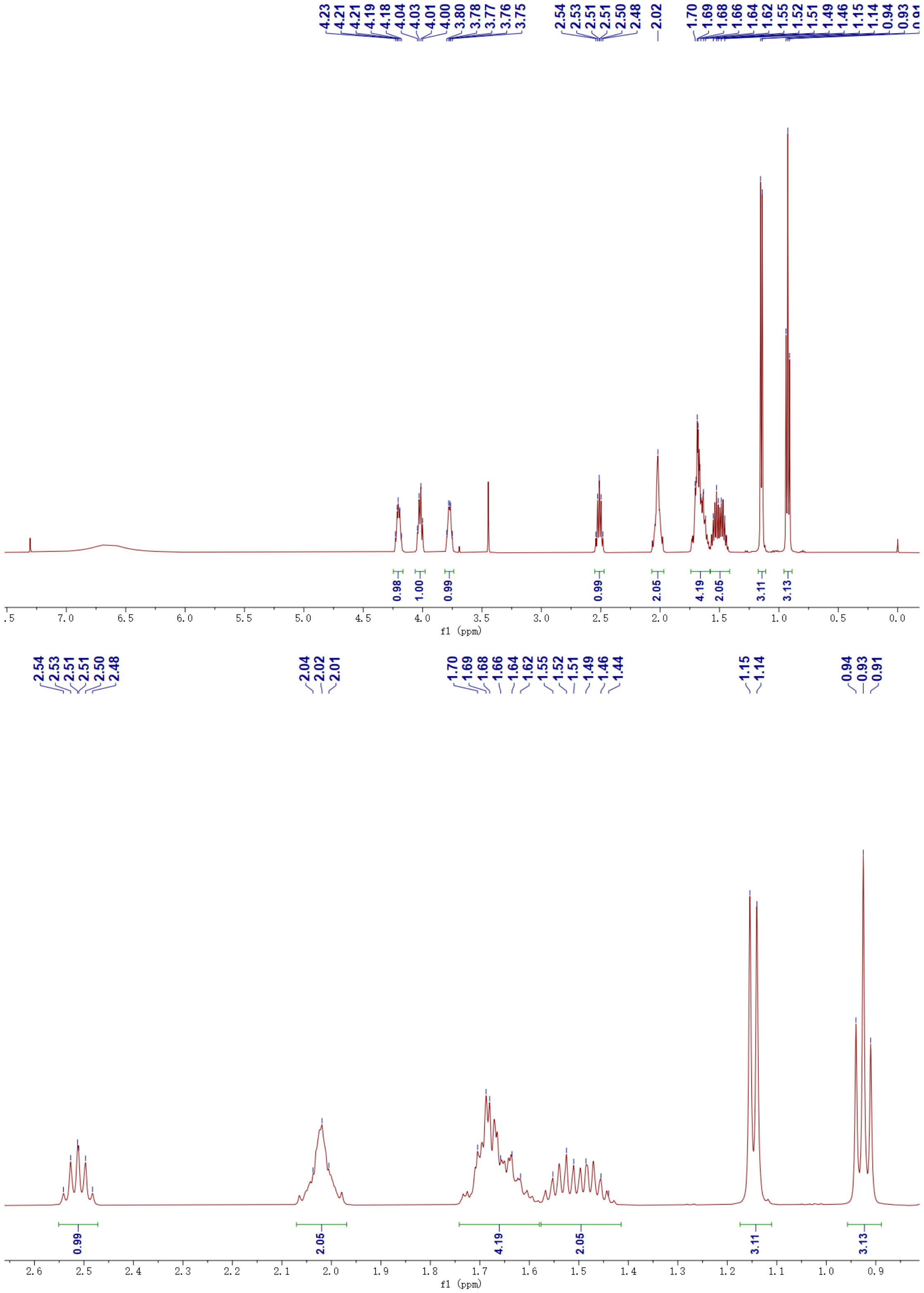

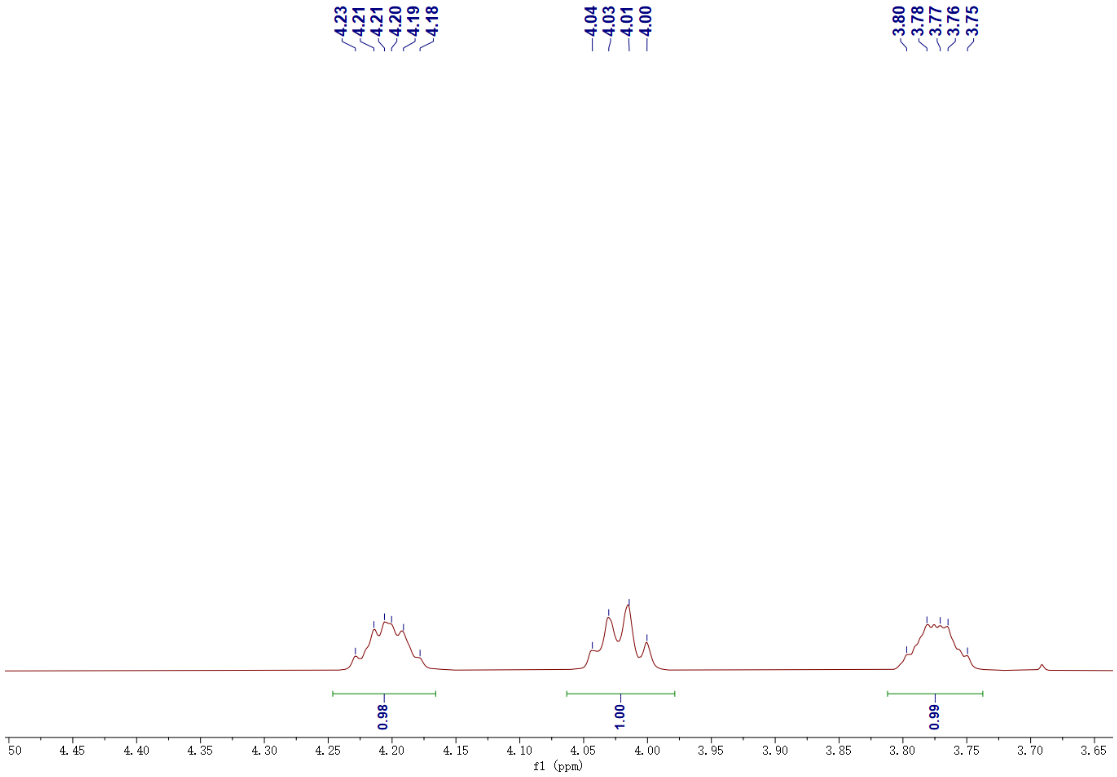

Figure S49. ^{13}C NMR and DEPT spectra ($CDCl_3$, 126 MHz) of compound **5**

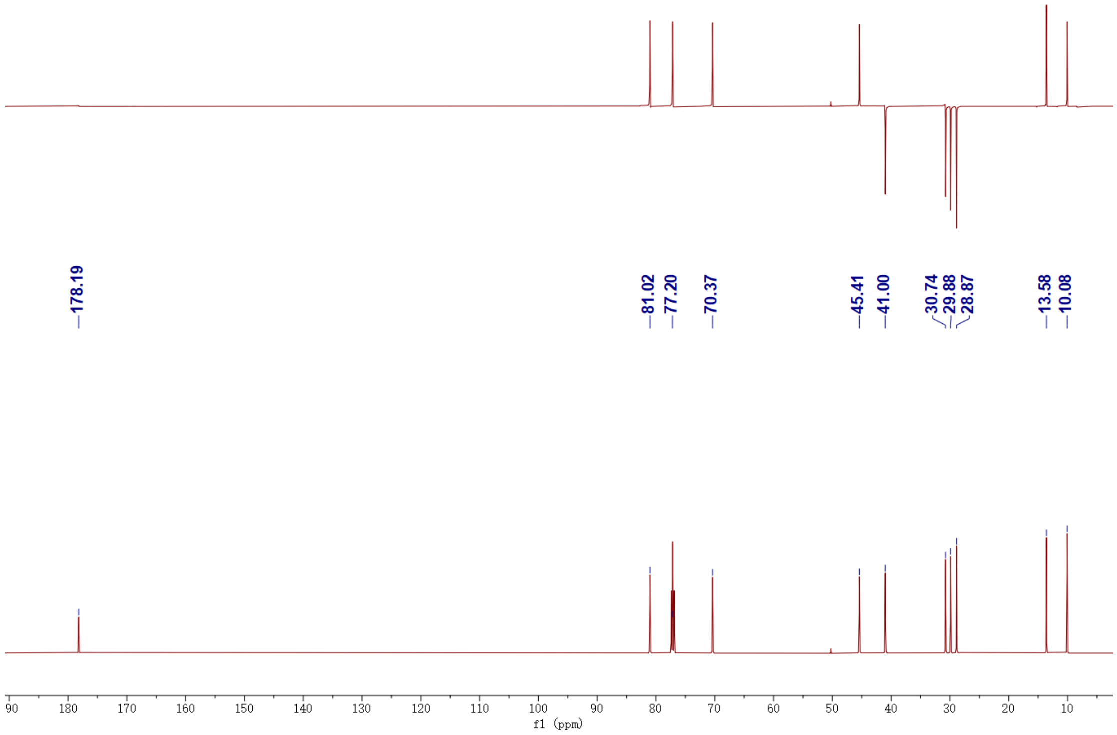

Figure S50. CD spectrum of compound **5**

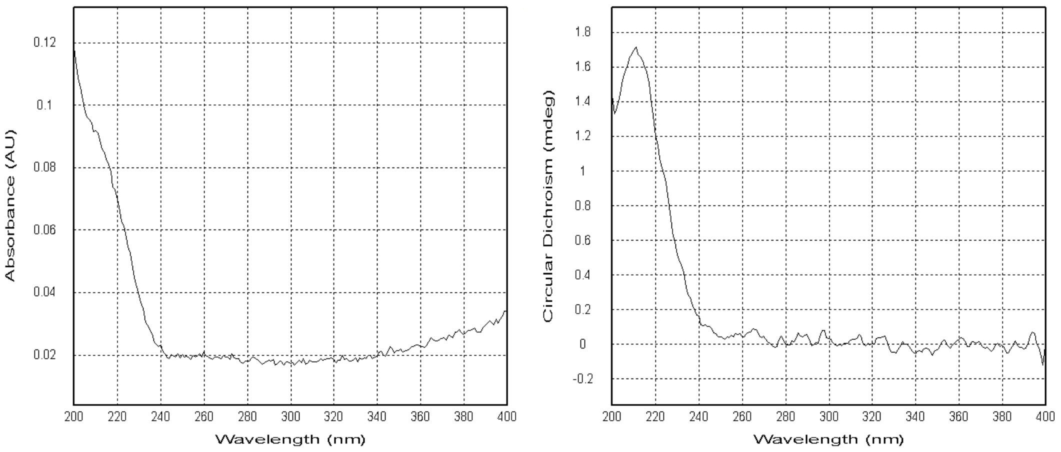

Figure S51. ESIMS spectrum of compound **6**

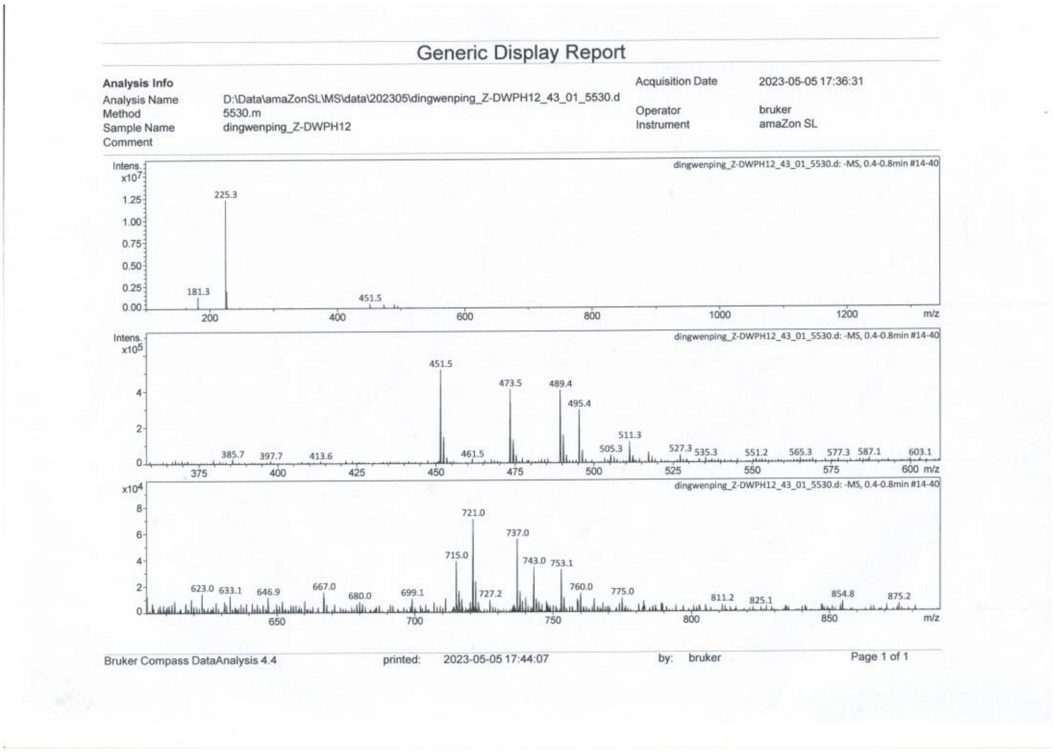

Figure S52. ^{1}H NMR spectrum (CD_3OD, 700 MHz) of compound **6**

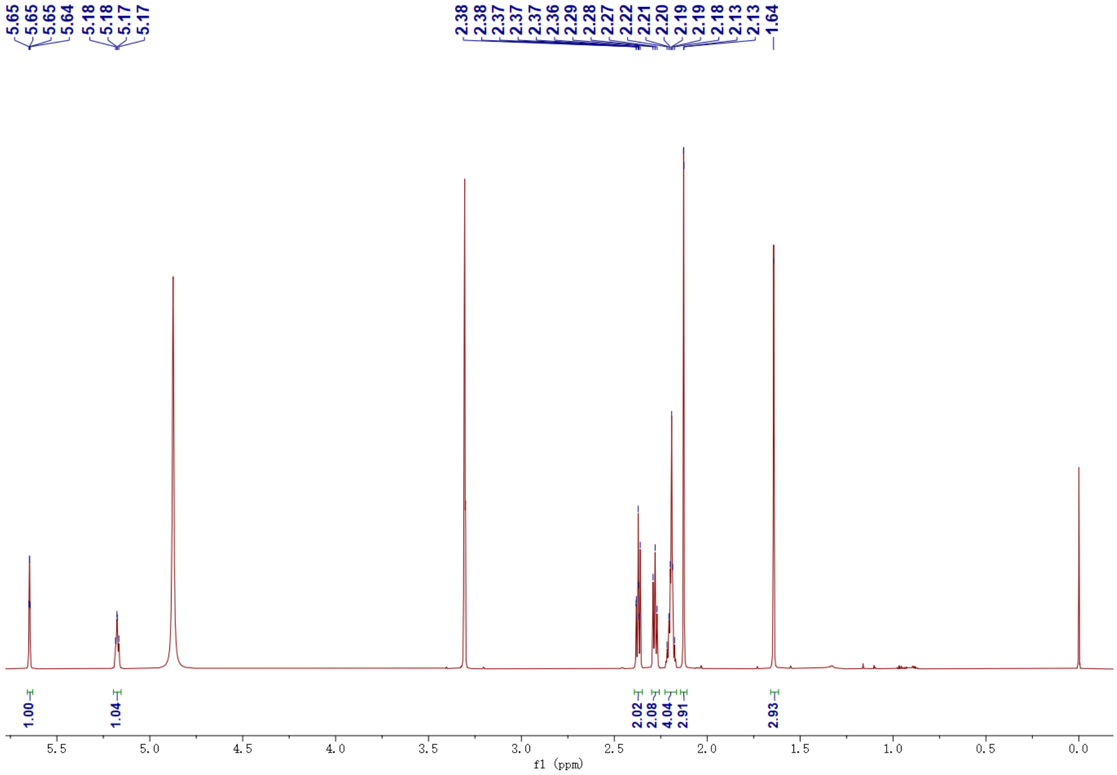

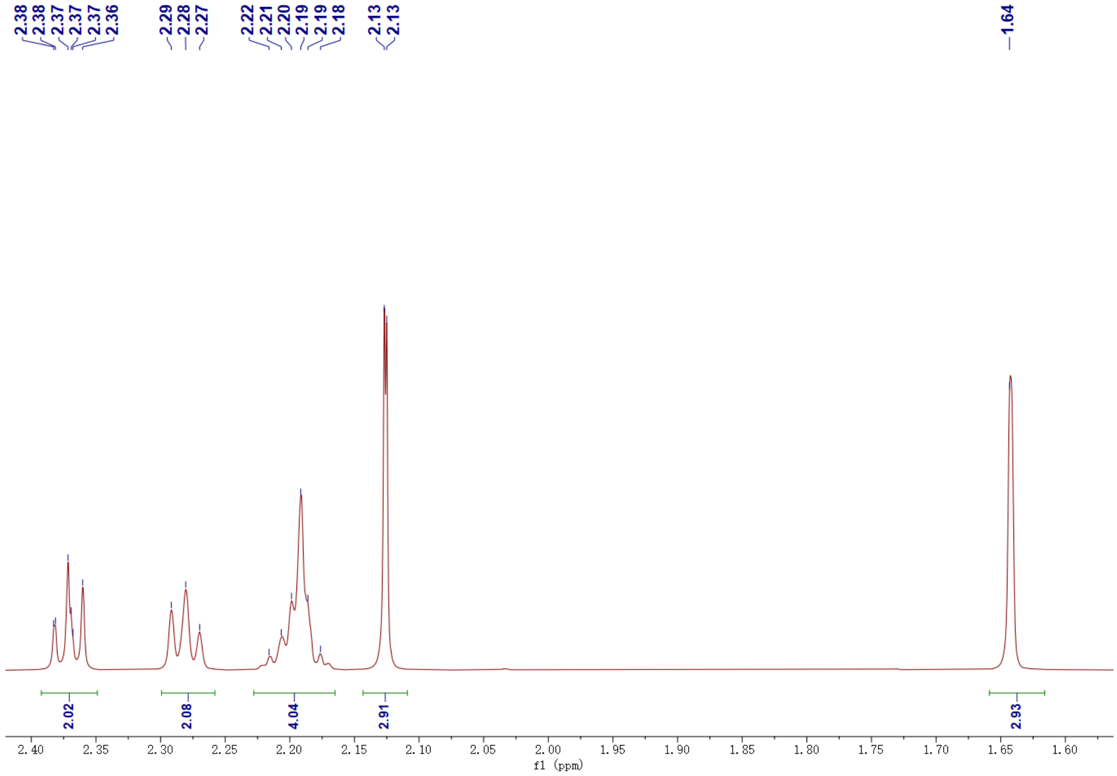

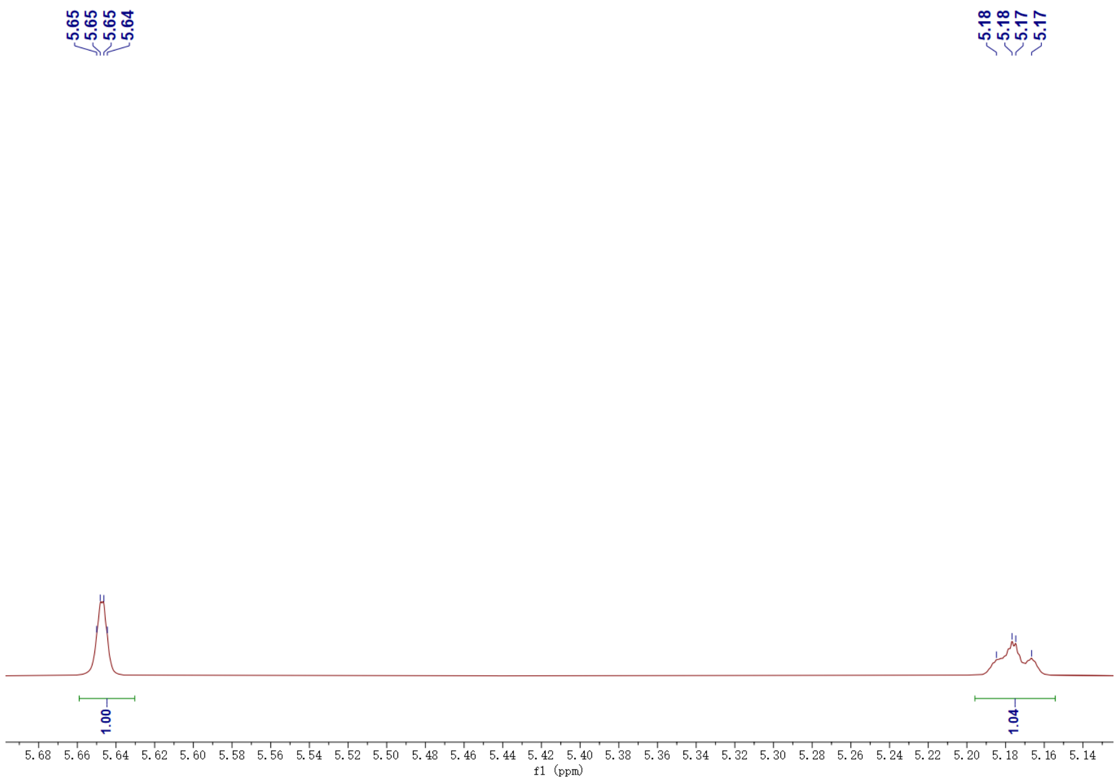

Figure S53. ^{13}C NMR and DEPT spectra (CD_3OD, 176 MHz) of compound **6**

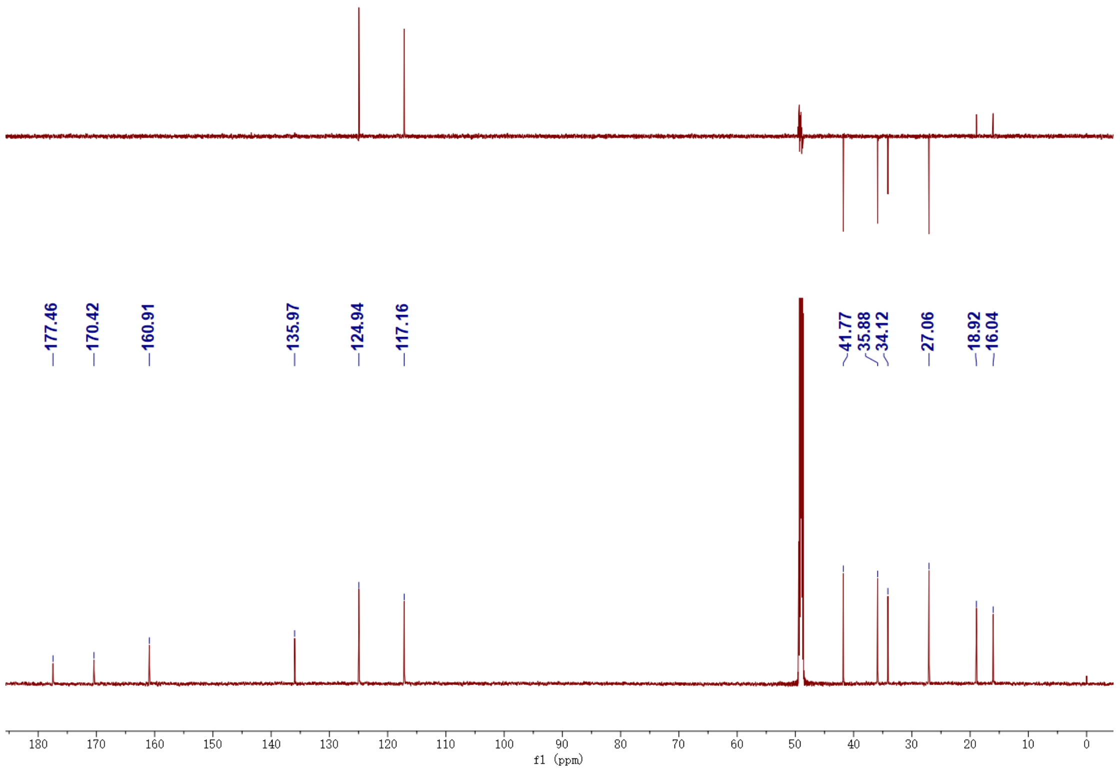

Figure S54. ESIMS spectrum of compound **7**

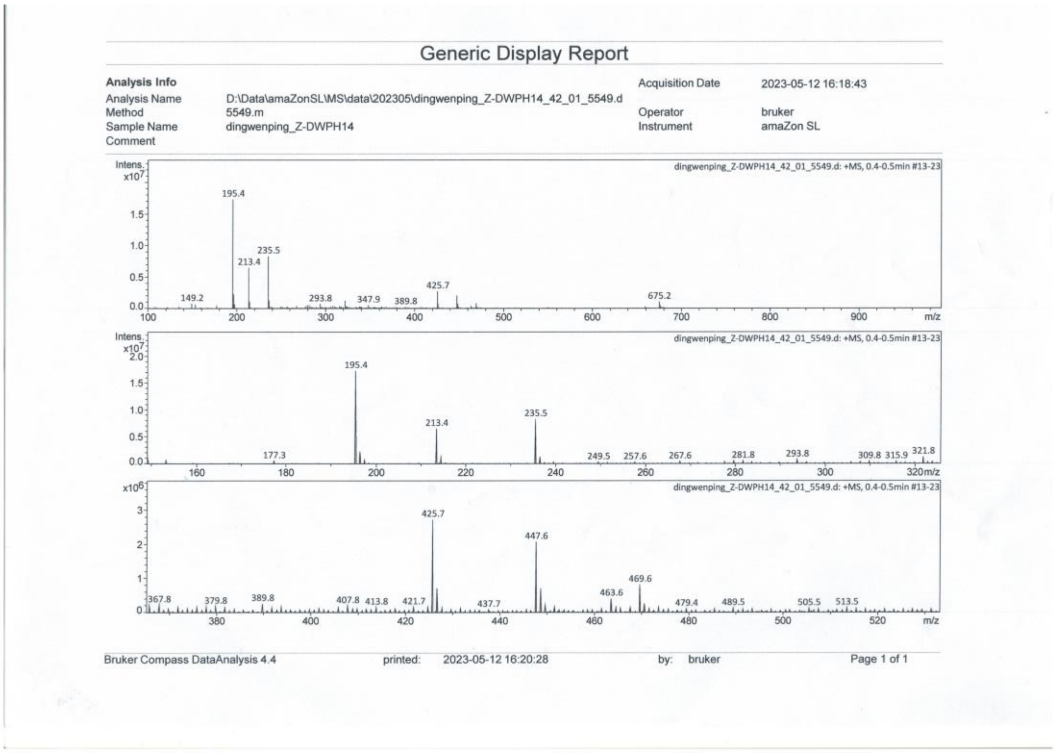

Figure S55. ^{1}H NMR spectrum (CD_3OD, 700 MHz) of compound **7**

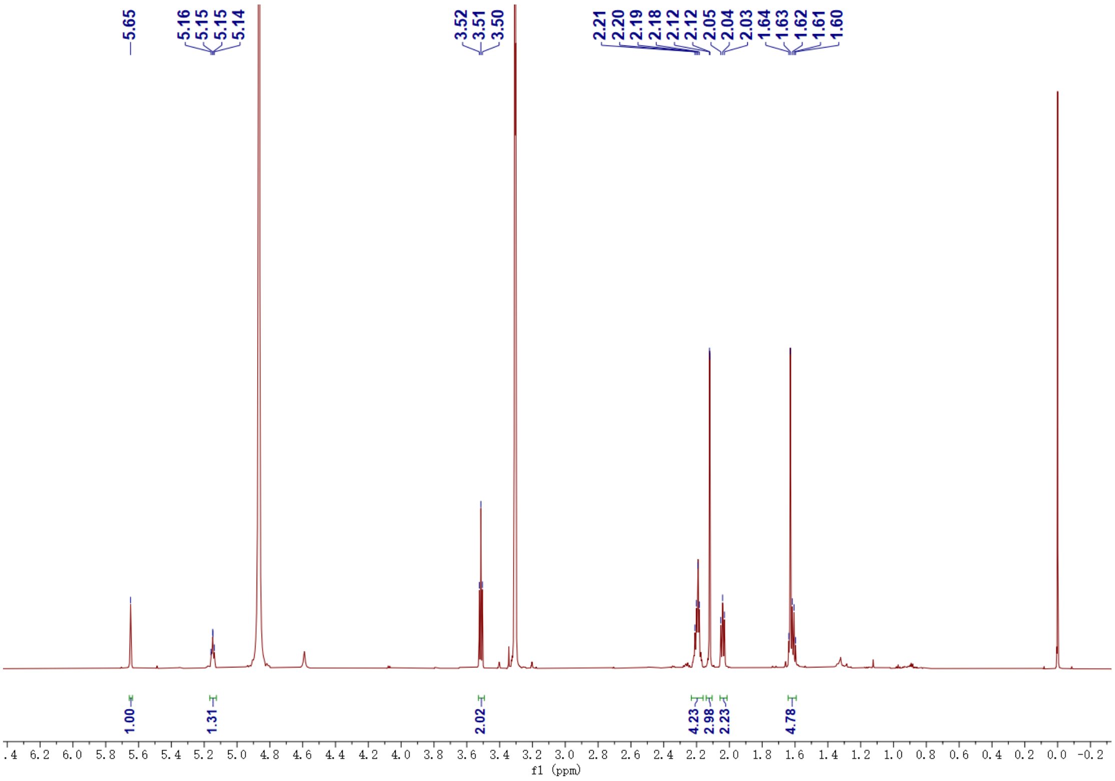

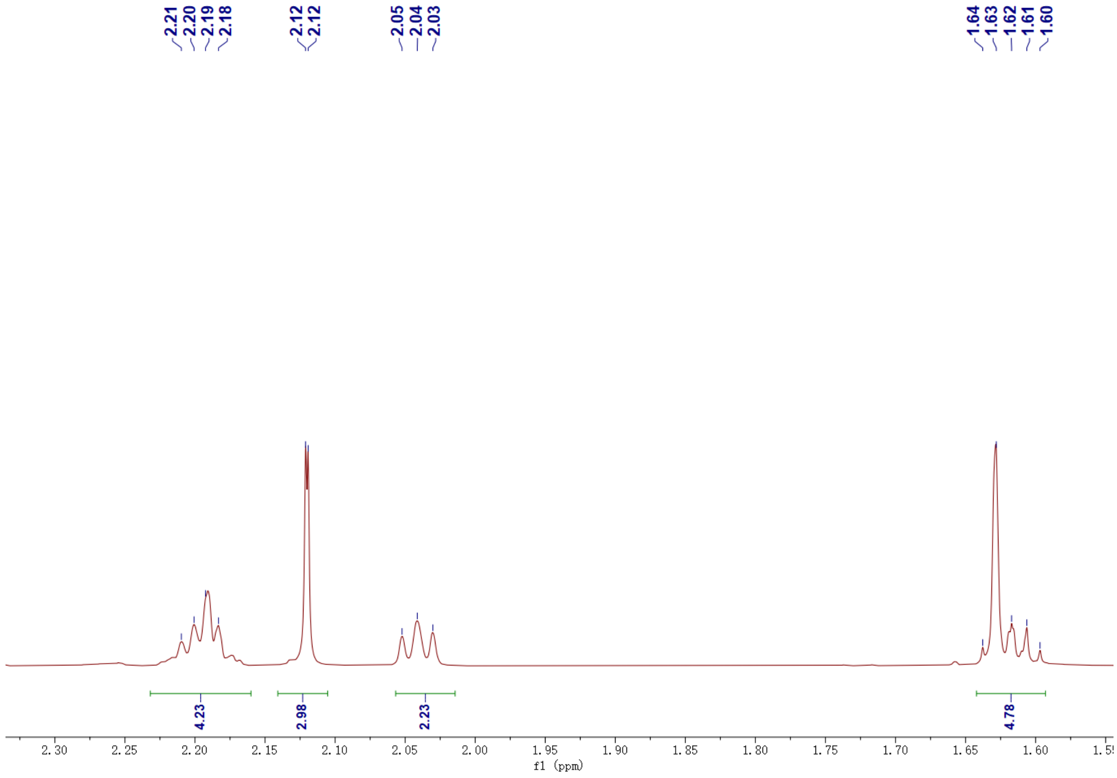

2.21
2.20
2.19
2.18
2.12
2.12
2.05
2.04
2.03
1.64
1.63
1.62
1.61
1.60
4.23
2.98
2.23
4.78
f1 (ppm)

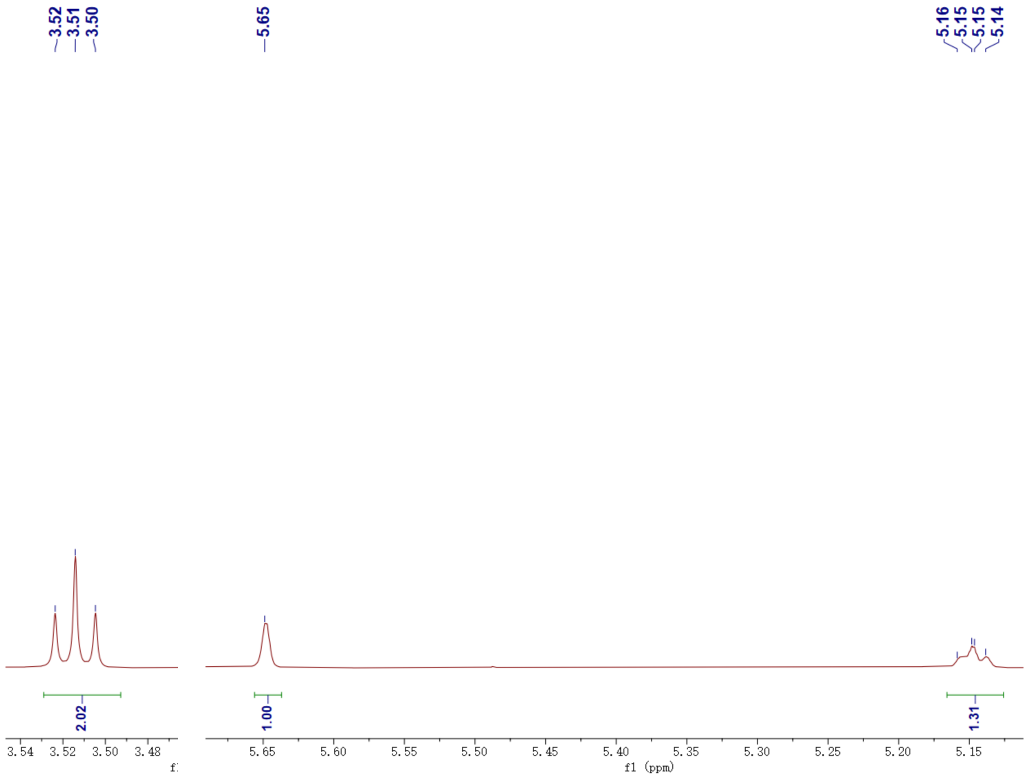

3.52
3.51
3.50
5.65
5.16
5.15
5.15
5.14
2.02
1.00
1.31
f1 (ppm)

Figure S56. ^{13}C NMR and DEPT spectra (CD_3OD, 176 MHz) of compound **7**

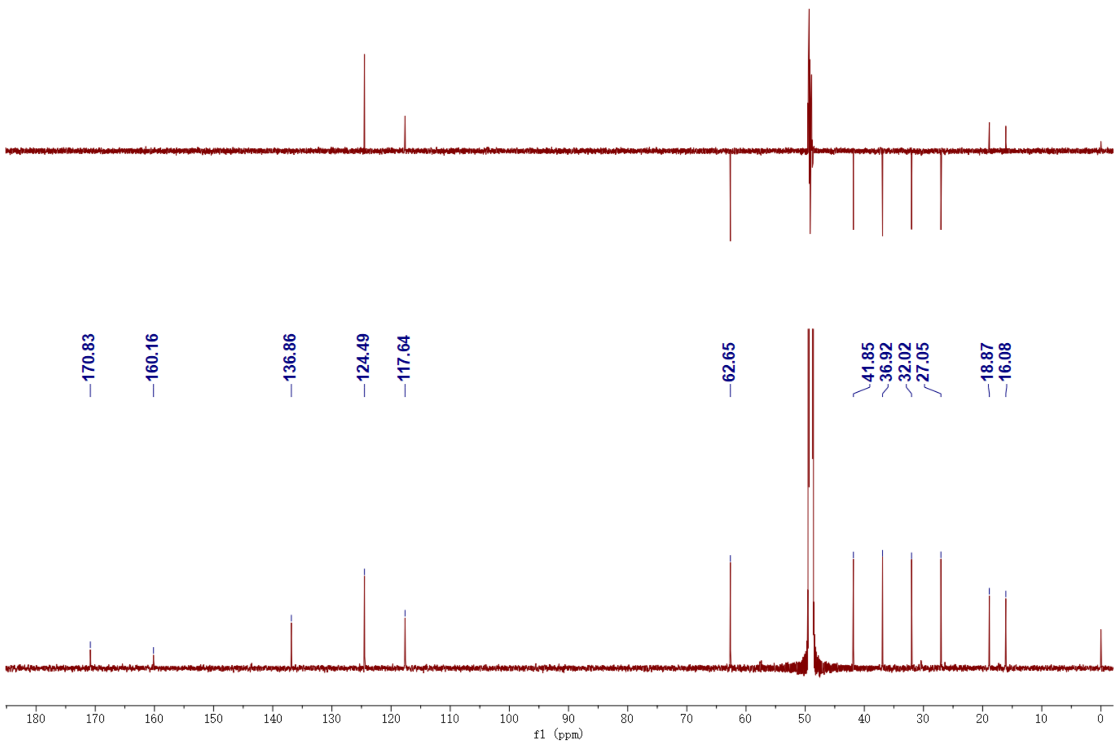

Calculated the specific rotations of 2a and 2b

Figure S57. The computational configurations of **2a** and **2b**

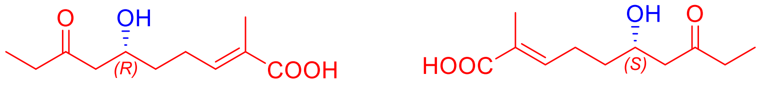

2a **2b**

Figure S58. Optimized geometries of 6 dormant conformers of **2a** (**2a-1**, **2a-2**, **2a-3**, **2a-4**, **2a-6**, and **2a-7**, respectively) at the B3LYP-D3(BJ)/6-31G* level of theory in methanol with the IEFPCM solvent

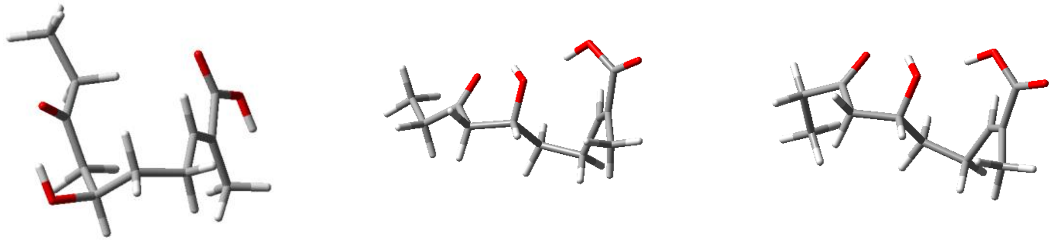

2a-1 **2a-2** **2a-3**

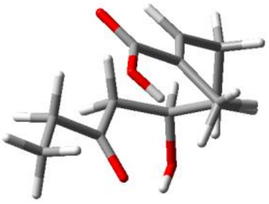

2a-4

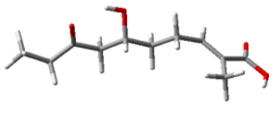

2a-6

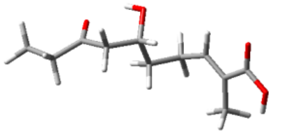

2a-7

Table S5. Conformational analysis of the optimized **2a** at the B3LYP-D3(BJ)/6-31G* of theory in methanol with the IEFPCM solvent

Conformers	*G* (kcal/mol) [a]	*ΔG* (kcal/mol) [b]	Population [c]
**2a-1**	-458543.451338019	1.2211	7.83%
**2a-2**	-458544.672471294	0.0000	61.58%
**2a-3**	-458543.576839897	1.0956	9.68%
**2a-4**	-458542.528899214	2.1436	1.65%
**2a-6**	-458543.851689011	0.8208	15.4%
**2a-7**	-458543.033416765	1.6391	3.86%

[a] The Gibbs free energy; [b] The relative Gibbs free energy; [c] The Boltzmann distribution of each conformer.

Figure S59. Optimized geometries of 6 dormant conformers of **2b** (**2b-1**, **2b-2**, **2b-3**, **2b-5**, **2b-6**, and **2b-7**, respectively) at the B3LYP-D3(BJ)/6-31G* level of theory in methanol with the IEFPCM solvent

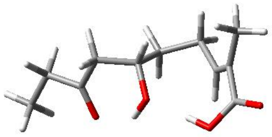

2b-1

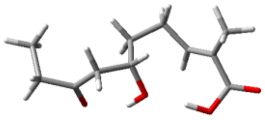

2b-2

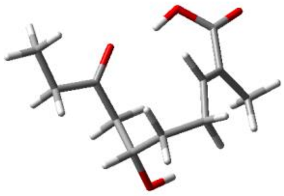

2b-3

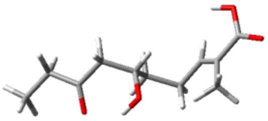

2b-5

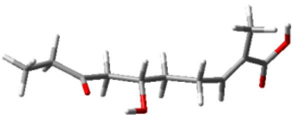

2b-6

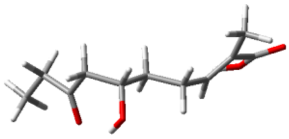

2b-7

Table S6. Conformational analysis of the optimized **2b** at the B3LYP-D3(BJ)/6-31G* of theory in methanol with the IEFPCM solvent

Conformers	*G* (kcal/mol) [a]	*ΔG* (kcal/mol) [b]	Population [c]
**2b-1**	-458544.675608841	0.0000	44.58%
**2b-2**	-458543.576839897	1.0988	6.97%

**2b-3**	-458544.363109164	0.3125	26.3%
**2b-5**	-458543.686654041	0.9890	8.39%
**2b-6**	-458543.849178973	0.8264	11.04%
**2b-7**	-458543.018984049	1.6566	2.72%

[a] The Gibbs free energy; [b] The relative Gibbs free energy; [c] The Boltzmann distribution of each conformer.
